# The Differences in the Proteome Profile of Cannabidiol-Treated Skin Fibroblasts following UVA or UVB Irradiation in 2D and 3D Cell Cultures

**DOI:** 10.3390/cells8090995

**Published:** 2019-08-28

**Authors:** Agnieszka Gęgotek, Sinemyiz Atalay, Pedro Domingues, Elżbieta Skrzydlewska

**Affiliations:** 1 Department of Analytical Chemistry, Medical University of Bialystok, Mickiewicza 2D, 15-222 Bialystok, Poland; 2 Mass Spectrometry Center, Department of Chemistry, University of Aveiro, 3810-193 Aveiro, Portugal

**Keywords:** cannabidiol, skin fibroblasts, UV radiation, proteomics, 2D and 3D cell cultures

## Abstract

Cannabidiol (CBD), as the only phytocannabinoid that has no psychoactive effect, has both antioxidant and anti-inflammatory effects, and thus might be suggested as a cytoprotective compound against UV-induced metabolic changes in skin cells. Therefore, the aim of this study was to investigate the level of protective CBD activity by evaluating the proteomic profile of 2D and 3D cultured skin fibroblasts models following exposure to UVA and UVB radiation. The CBD cytoprotective effect against UV-induced damage in 2D and 3D cultured fibroblasts were different. The main alterations focus on the range of cell reaction and involved different proteins associated with various molecular functions. In the 2D cultured cells, following UV radiation, the major changes were associated with proteins involved in antioxidant response and inflammation, while, in the 3D cultured fibroblasts, CBD action against UV induced changes were mainly associated with the activation of signalling pathways. Therefore, the knowledge of the CBD action in a multilayer skin cells model allowed for the prediction of changes in cell-cell interactions and skin cell metabolism. Knowledge about the lower protective effect of CBD in 3D cultured fibroblasts should be taken into account during the design of UV light protection.

## 1. Introduction

Cannabidiol (CBD) is a major, biologically active, phytocannabinoid that has recently become increasingly used in cytoprotection [1,2]. Since CBD does not show any psychoactive action, there are many efforts focused on the use of the anti-inflammatory and protective properties of CBD in different therapies [3,4]. To date, it has been shown that CBD can act as a reactive oxygen species (ROS) scavenger and as an antioxidant in cells under stress conditions [5]. Moreover, also CBD anti-apoptotic activity has been previously described, showing that CDB has a positive effect on cell survival [6]. CBD may also show protective actions against UV-induced oxidative stress in skin cells exposed to sunlight. The UVA and UVB regions of the spectrum reaching the human skin have damaging effects which influence the cell function by direct formation of ROS and modifying cellular compounds such as proteins, lipids and nucleic acids [7]. As a result, changes in cellular metabolism are observed when skin cells are exposed to UV radiation, leading to impaired skin condition and functioning [8].

The harmful action of UV radiation is strongly dependent on its power to penetrate into the skin layers [9]. This organization of the skin organ is impossible to analyze in the commonly used two-dimensional (2D) culture model. Therefore, there is an indisputable need to use three-dimensional (3D) cell culture system to reduce the knowledge gap between 2D cell cultures and physiological tissues. Moreover, it is generally recognized that the cell phenotype characterized in 3D cell cultures more closely reflects the in vivo conditions in tissues, in forming cell-cell interaction as well as differentiation patterns [10]. In addition, the effect of various chemical agents has been shown to be different in 2D and 3D culture models [11]. It has been also shown that the expression of proteins in 2D-monolayers and in 3D-spheroids is significantly different [12]. This has been associated with both the availability of the culture medium and with the signal transduction between cells in these two types of culture systems. Therefore, in this study, UV-induced changes, as well as cytoprotective CBD effects, on the proteomic profile of 2D cultured skin fibroblasts were compared with changes observed in 3D cultured cells.

## 2. Material and Methods

### 2.1. Cell Culture and Treatment

Human skin fibroblasts, CRL-1474 (obtained from the American Type Culture Collection, Manassa, VA, USA), were cultured as a monolayer according to the protocol for 2D cell system using growth medium Dulbecco’s Modified Eagle Medium (DMEM) with 10% fetal bovine serum (FBS) supplemented with 50 U/mL penicillin and 50 μg/mL streptomycin. All sterile and intended for cell culture reagents were obtained from Gibco (Grand Island, NY, USA).

The 3D culture was carried out in 6-well AlgiMatrix plates activated by AlgiMatrix^®^ Firming Buffer and using the growth medium as above. All reagents for 3D culture were obtained from Life Technologies (Carlsbad, CA, USA). All cells were cultured in a humidified atmosphere of 5% CO_2_ at 37 °C.

When cells reached 90% confluence, they were washed with PBS buffer (37 °C) and irradiated with the following UV doses: UVA-20 J/cm^2^ and UVB-200 mJ/cm^2^ (Bio-Link Crosslinker BLX 312/365; Vilber Lourmat, Eberhardzell, Germany). The cells were irradiated at a distance of 15 cm from the 6 lamp assembly at 6 W each, which corresponds to 4.2 mW/cm^2^ and 4.08 mW/cm^2^ for UVA (365 nm) and UVB (312 nm), respectively. Exposure doses were chosen corresponding to 70% viability of cells cultured in monolayer [13]. To avoid heat stress and medium components oxidation, cells were exposed to UV radiation in PBS (4 °C). To observe the effect of CBD, following irradiation cells were incubated for 24 h in medium containing 4 µM of CBD. Used concentration of CBD did not alter the morphology or proliferation of fibroblasts [14] and cells viability measured by MTT test (data not shown). During this incubation the medium did not contain FBS to avoid the influence on proteomic results. Control cells were cultured in parallel with no treatment.

Following incubation, 2D cultured cells were collected by scraping and 3D cultured cells were collected using AlgiMatrix^®^ Dissolving Buffer. All cells were washed, dissolved in lysis buffer (10 mM Tris-HCl pH 7.4, 1 mM EDTA, 1% Triton X-100, 0.1% SDS), lysed through sonification on ice, and centrifuged (15 min, 12,000× *g*). The supernatant was mixed 1:1 with sample loading buffer (Laemmli buffer containing 5% 2-mercaptoethanol) and heated at 95 °C for 10 min to denature proteins.

### 2.2. Proteomic Analysis

**Figure 1:**
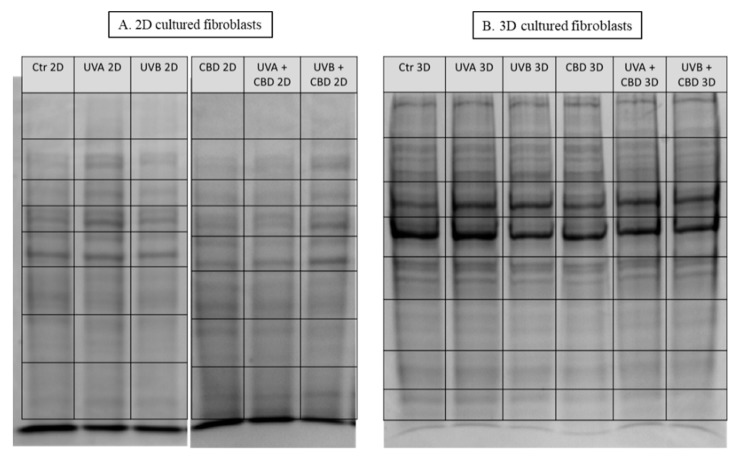
SDS-PAGE separation and staining with Coomassie Brilliant Blue R-250 of proteins from control skin fibroblasts and irradiated with UVA (20 J/cm^2^), UVB (200 mJ/cm^2^) or/and treated with cannabidiol (CBD, 4 µM) in two-dimensional (2D) (**A**) or three-dimensional (3D) culture model (**B**). The grid indicates the borders of the protein migration zones.

Samples were separated on 10% Tris-Glycine SDS-PAGE gels and stained with Coomassie Brilliant Blue R-250. Complete lanes were cut out of the gel, sliced into eight sections (Figure 1) and in-gel digested overnight with trypsin (Promega, Madison, WI, USA). The isolated peptide mixture was extracted from the gel fractions, dried and dissolved in ACN + 0.1% formic acid. The resulting mixtures were separated using an Ultimate 3000 (Dionex, Idstein, Germany) onto a 150 mm × 75 mm PepMap RSLC capillary analytical C18 column with 2 μm particle size (Dionex, LC Packings). The peptides eluted from the column were analyzed using a Q Exactive HF mass spectrometer with an electrospray ionization source (ESI) (Thermo Fisher Scientific, Bremen, Germany). The details of the protein separation and peptide analysis by LC-MS/MS have been reported previously [15].

### 2.3. Protein Identification, Grouping, and Label-Free Quantification

Processing of the raw data generated from LC-MS/MS analysis was carried out using Proteome Discoverer 2.0 (Thermo Fisher Scientific) and Sequest HT (Sequest HT algorithm, license Thermo Scientific, registered trademark of the University of Washington, Seattle, WA, USA). The following search parameters were used for protein identification: peptide mass tolerance set to 10 ppm, MS/MS mass tolerance set to 0.02 Da, up to two missed cleavages allowed, cysteine carbamidomethylation and carboxymethylation, methionine oxidation, MDA-lysine, acrolein-cysteine and 4-HNE-cysteine/lysine/histidine adducts formation set as a dynamic modification [16]. Input data were searched against the UniProtKB-SwissProt database (taxonomy: *Homo sapiens*, release 2018-04). The proteins quantification, including proteins modified by lipid peroxidation products was done based on the corresponding peak area analyzing.

### 2.4. Statistical Analysis

Analysis of each sample were performed in three independent experiments. Results from individual protein label-free quantification were normalized by the sample median and log-transformed. Missing were replaced with half of the threshold value (2 × 10^4^). Data were analyzed using the standard statistical analysis methods, including univariate analysis (one-way ANOVA), and only protein with FDR corrected significant Q-value were taken into account in the discussion. Quality control and biostatistics analysis, including principal component analysis (PCA), volcano plots, heat map and clustering were performed with free available software: MetaboAnalyst 4.0 (http://www.metaboanalyst.ca). The analysis of proteins molecular functions were prepared using the Panther Classification System (http://pantherdb.org). The analyzes were based on protein ID list generated during ANOVA test, PCA or heatmapping. The search was set to a specific organism (Homo sapiens) and functional classification viewed in pie charts was performed to obtain molecular or biological functions.

## 3. Results

**Figure 2:**
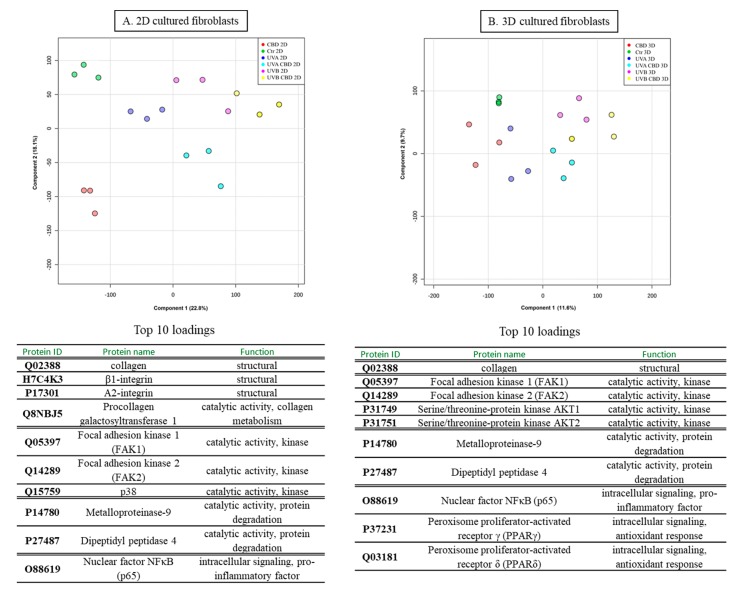
Principal component analysis (PCA) and top 10 loadings for component 1 (grouped according to their functions) of control skin fibroblasts and irradiated with UVA (20 J/cm^2^), UVB (200 mJ/cm^2^) or/and treated with cannabidiol (CBD, 4 µM) in two-dimensional (2D) (**A**) or three-dimensional (3D) culture model (**B**).

**Figure 3:**
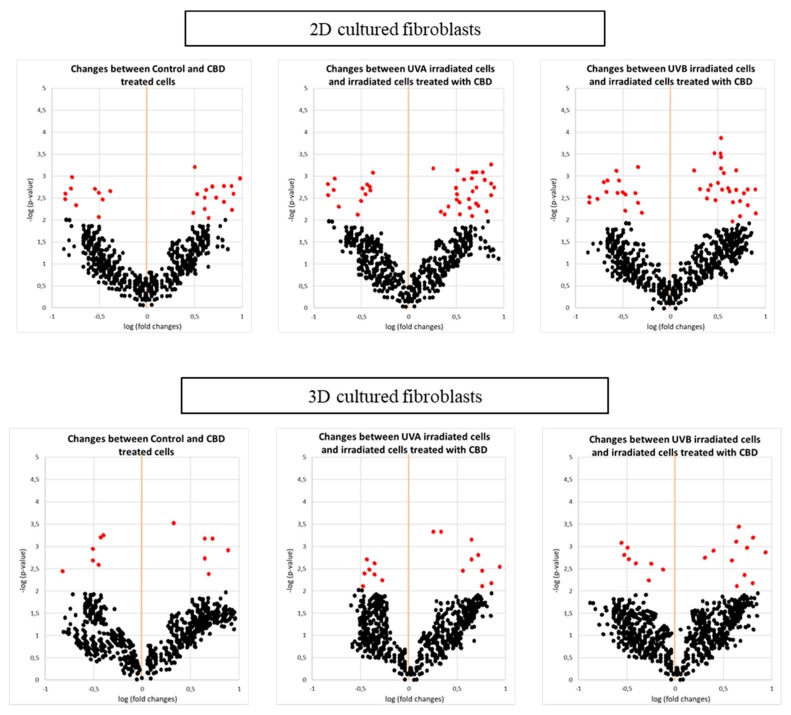
Volcano plots comparing the effect of cannabidiol (CBD, 4 µM) on control fibroblasts and UVA (20 J/cm^2^) or UVB (200 mJ/cm^2^) irradiated cells in two-dimensional (2D) or three-dimensional (3D) culture model. Significant features (in red) had *p* < 0.05.

**Figure 4:**
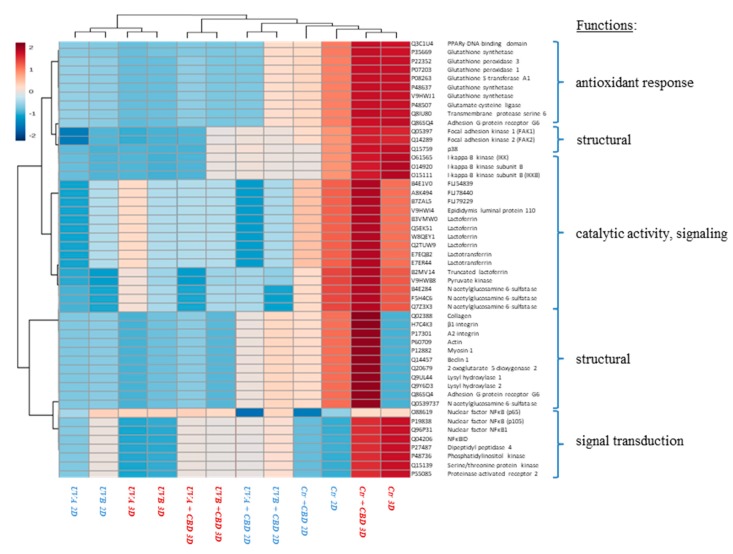
Heat map and clustering for the top 50 proteins (with smallest Q-value) from control skin fibroblasts and irradiated with UVA (20 J/cm^2^), UVB (200 mJ/cm^2^) or/and treated with cannabidiol (CBD, 4 µM) in two-dimensional (2D) or three-dimensional (3D) culture model.

In this study, we report the effect of CBD treatment on changes in the protein expression caused by UVA and UVB radiation in human skin 2D and 3D cultured fibroblasts. For all treatment conditions, 650 proteins with at least two unique peptides were found in all samples and their levels were determined. The list showing the identification of these proteins, as well as their average levels in each group is presented as a supplementary table (Supplementary Table S1). Obtained data tested with the principal component analysis (PCA) showed a good clustering pattern where all treatment conditions were clearly clustered distinct groups, in the case of 2D cultured fibroblasts (Component 1—22.8%; Component 2—18.1%; Figure 2A), while the clustering was not so clear in the case of 3D culture system (Component 1—11.6%; Component 2—9.7%; Figure 2B). In 2D cultured cells, univariate statistical analysis of the individual proteins identified 42 proteins with significantly different expression in UVA/UVB irradiated fibroblasts and 65 proteins with differentiated expression following CBD treatment (29 proteins compared to control, 41 proteins compared to UVA, and 48 proteins compared to UVB). In the case of 3D cultured cells, CBD treatment and UVA/UVB radiation caused significant changes in protein expression (29 differentiated proteins in UVA/UVB irradiated cells, 36 differentiated proteins following CBD treatment). The volcano plots showed significant differences when comparing the effect of CBD on control fibroblasts and UVA/UVB irradiated cells in both 2D and 3D culture systems (Figure 3), although the 3D system showed less significant features. We created a dendrogram with a two-dimensional hierarchical clustering, using the top 50 proteins from control skin fibroblasts (Figure 4). The primary split in the upper hierarchical dendrogram shows that the samples clustered independently into treatments and culture systems. The individual proteins were clustered into antioxidant, structural signalling catalytic, structural and signal transduction activity (Table 1, Figure 4). Moreover, most of these proteins constitute the top 10 loadings from the PCA and grouped according to their functions were shown in Figure 2. 

**Figure 5:**
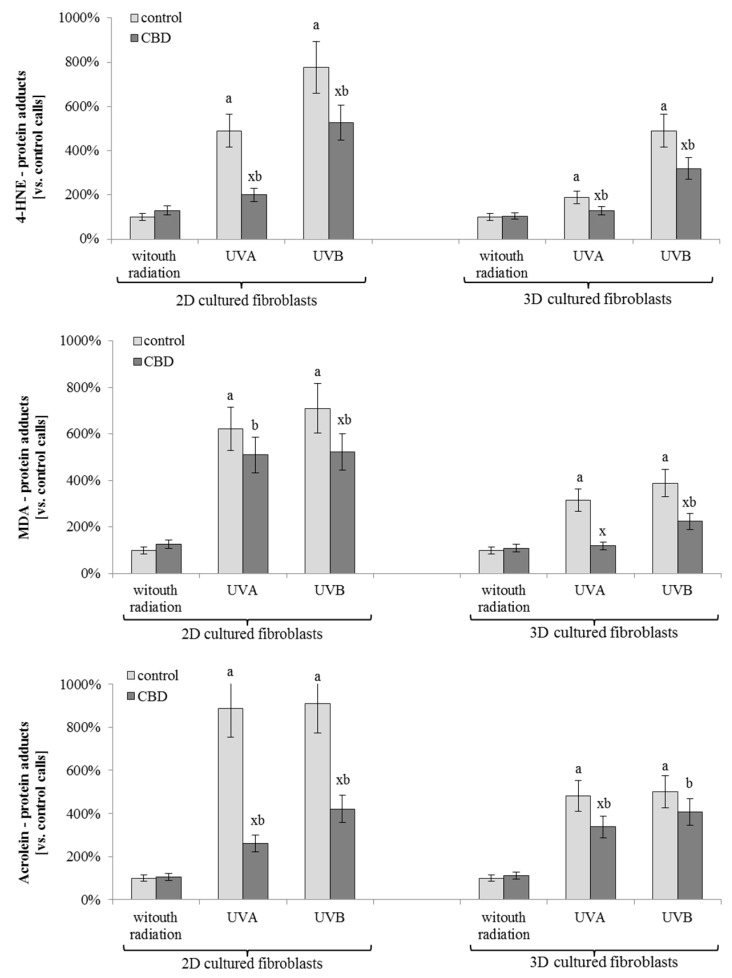
The level of proteins modified by lipid peroxidation products (malondialdehyde (MDA), 4-hydroxynonenal (4-HNE), acrolein) adducts formation in fibroblasts irradiated with UVA (20 J/cm^2^) and UVB (200 mJ/cm^2^) or/and treated with cannabidiol (CBD, 4 µM) in two-dimensional (2D) or three-dimensional (3D) culture model. Mean values ± SD of tree independent experiments are presented. x—statistically significant differences between CBD treated and corresponding non-treated cells, Q < 0.05; a—statistically significant differences vs. control group, Q < 0.05; b—statistically significant differences vs. CBD treated group, Q < 0.05.

**Figure 6:**
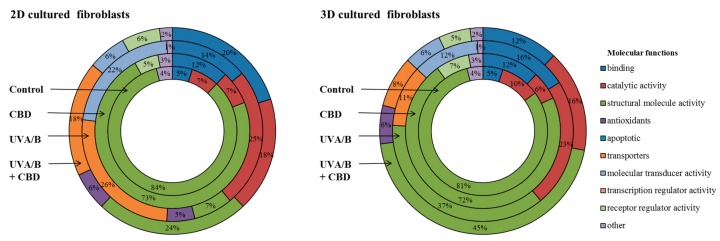
The molecular functions of proteins modified by lipid peroxidation products (malondialdehyde (MDA), 4-hydroxynonenal (4-HNE), acrolein) adducts formation in fibroblasts irradiated with UVA (20 J/cm^2^) and UVB (200 mJ/cm^2^) or/and treated with cannabidiol (CBD, 4 µM) in two-dimensional (2D) or three-dimensional (3D) culture model.

The results of this study showed significant differences in the level of protein-lipid peroxidation products adduct formation in cells treated with CBD, compared with non-treated cells (Figure 5). Moreover, in all cases, the level of adducts following UV radiation was higher in 2D than in 3D cultured fibroblasts. Nevertheless, the CBD treatment resulted in a decrease in the level of 4-HNE and MDA–protein adducts in UVB irradiated cells by approximately 40%, regardless of the used culture model. In the case of UVA irradiation, the CBD treatment decreased the level of 4-HNE and MDA—protein adducts by 15–65%, depending on the culture model and type of reactive aldehyde. The strongest CBD effect was observed in the level of acrolein-protein adducts in 2D cultured fibroblasts following UVA and UVB radiation. In this portion of the study, CBD treatment led to a decrease of around 80% compared to irradiated cells. The proteins that were modified by lipid peroxidation products were identified, quantified and grouped by their function. Due to the fact that the results for UVA and UVB radiation were similar, data were averaged and presented as one group. Therefore, it is possible to compare how the percentage of modified proteins with specified functions changes following UV radiation or CBD treatment (Figure 6). In CBD treated cells, the products of lipid peroxidation adduction were generated mainly on proteins with regulator receptor activity, which was not observed in other cell treatment groups. Alternatively, UV radiation favored adduct formation on proteins involved in transport and molecules transduction. These changes were observed at twice the level in 2D cultured cells, compared to 3D, UV irradiated cells, as well as compared to UV irradiated, CBD treated cells (Figure 6).

## 4. Discussion

Since UV radiation is one of the most common factors which damages human skin cells, there is a constant effort to develop novel cytoprotective compounds. It is extremely important to establish an experimental model as close as possible to physiological conditions to enable improvement in the field. It has been previously shown that cells in 3D culture differ in proteomic profile from 2D cultured cells mainly in terms of proteins that are the part of a functional network including structural proteins, chaperones, as well as oxidative stress-related proteins [17]. Moreover, 3D cultured cells respond more effectively than cells from monolayer to cytotoxic agents, such as hydrogen peroxide or heavy metals [11]. Therefore, to better understand skin cell metabolism, cell-cell interactions, and the reaction of these systems to the protective CBD action, this study examined changes in the proteomic profile of 2D cultured skin fibroblasts and compared these changes to what was observed in the 3D system.

UV radiation penetrates the skin affecting fibroblast metabolism via induction of oxidative stress [7]. In 2D cultures, all fibroblasts are equally exposed to UVA/UVB radiation, while in 3D cultures, cells are exposed to UVA/UVB to different degrees depending on the penetration of the radiation into the cell layers [18]. Consequently, in 2D cultured cells, we found that the collagen expression was decreased, compared to 3D cultured cells. These observations support the previously described UV-induced prolidase activation and increased collagen degradation in 2D in vitro cultured human skin fibroblasts [13]. Collagen remodelling in skin fibroblasts plays a crucial role in organizing tissue structures which are essential to motility during wound repair, development, and regulation of cell growth [19]. Therefore, the CBD-induced stimulation of the enzymes involved in collagen cross-linking, including lysyl hydroxylase that previously has been showed in 2D [20], as well as shown in this study in 3D cultures, prevents collagen degradation following UV radiation. The mechanism of collagen fibre movement in 3D cultured fibroblasts also has been previously correlated with α2,β1-integrin [21], which was also strongly increased following UV radiation and CBD treatment in this study. Further, it has been shown in other cell lines that the 3D culture system enhances the β1-integrin-dependent pathway and provides an increase in cell survival [22].

3D cultured fibroblasts exhibit more elongated and branched shapes with fewer actin stress fibres compared with cells on flat surfaces [23]. Moreover, the migration of these cells is altered, a characteristic attributed to the spatial reorganization of the actin cytoskeleton [22]. As shown in this study, the expression of structural proteins, such as actin or myosin, were most susceptible to UV radiation in both 2D and 3D cell cultures. However, the multilayer structure effectively protects these proteins against modification, allowing 3D cultured fibroblasts to keep the physiological shape and the ability to migrate. Additionally, CBD decreased the UV-induced expression of metalloproteinases, thus preventing damage in the intracellular matrix [24]. CDB also protects structural proteins against UV-induced decreases in their expression. This observation is in agreement with previous data showing that CBD, through the activation of p38 MAPK, induced actin reorganization and cellular motility in microglial cells [25]. CBD also affects other signalling pathways connected with kinase activity. The most visible changes in the protein level are observed in the case of FAK (focal adhesion kinase), AKT (serine/threonine-specific protein kinase) and PI3K (phosphoinositide 3-kinase), where CBD strongly increases expression. This further protects cells against harmful UV effects. The PI3K/AKT pathway plays a predominant role in cell proliferation, survival, and progression [26]. It is known that both AKT and PI3K have several active binding sites specific for CBD [27]. However, it is not clear how the CBD-kinase adduct formation affects the enzymatic activity of these proteins. Our data from Table 1 showed enhanced levels of these kinases, suggesting that CBD may stimulate protein synthesis or prevent degradation. However, recent publications have shown that CBD-kinase adducts formation significantly stimulates the activity of AKT and PI3K in mice spinal cord cells or human ligament stem cells [28,29], but inhibits these molecules in breast cancer cells [28]. In the case of kinase FAK, we observed an increased expression by CBD in both 2D and 3D cultured fibroblasts. Other researchers have shown that in CBD treated cancer cells, such as glioma cells, the activity of this kinase is significantly decreased, providing evidence that this compoung might be useful in anti-cancer therapy [30]. The cell culture model also affects the mentioned kinase expression. 3D cultured fibroblasts had higher levels of FAK, AKT and PI3K than 2D cultured cells, which is related to stronger intercellular signalling. Similar differences have been found previously in 2D and 3D cultured cancer cells [31]. Moreover, CBD influences the viability of 2D cultured cells by decreasing the level of enzymes involved in protein degradation (peptidases, cathepsin S). However, at the same time, CBD increased the level of beclin-1, favouring autophagosome formation, which has previously been shown only in breast cancer cells [32]. This suggests a role for CBD in modulating the cross-talk between apoptosis and autophagy in cells under pathological conditions [33].

Furthermore, CBD disrupts the pro-inflammatory pathway associated with NFκB (nuclear factor κ-light-chain-enhancer of activated B cells). This disruption is due to CBD-induced stimulation of kinase IKK (IκB kinase) which is associated with IκB (inhibitor of κ-light-chain-enhancer of activated B cells) degradation and downstream destruction of the NFκB complex [34]. This effect of CBD, shown in both 2D and 3D cultured fibroblasts, indicated that CBD participates in the anti-inflammatory signalling pathway. Additionally, CBD decreases UV-induced expression of NFκB subunits, in 2D cultured fibroblasts similarly as has been shown in previous data [35,36]. CBD anti-inflammatory action was also visible in 2D cultured and UVB irradiated cells as a decrease in prostaglandin G/H synthase expression was observed. It was shown that CBD leads to decreased prostaglandin generation, including prostaglandins E and D that are involved in pro-inflammatory signalling [37]. However, these anti-inflammatory effects of CBD were not observed in 3D cultured fibroblasts, which may be associated with the lack of UV radiation penetrating the inner cell layers. This postulation is supported by the fact that chemical agents affect multilayer cultured cells to a lesser extent than in classical monolayer culture [11].

Alternatively, in 3D cultured cells, following UV radiation as it is shown in Table 1, CBD causes stronger changes in the expression of proteins involved in signal transduction pathways, including receptors, than in 2D cultured cells. In this mechanism, a PPARγ (peroxisome proliferator-activated receptor γ), a characteristic nuclear membrane protein responsible for anti-inflammatory cell reactions, deserves special attention [38]. Enhanced levels of PPARγ suggest increased capability to dimerize and become active, resulting in the expression of anti-inflammatory proteins [39]. Therefore, it is hypothesized that CBD protects cells against inflammation mainly through PPARγ activation [40,41]. Moreover, CBD may affect PPARγ activity in 2D cultured fibroblasts directly by decreasing the level of NFκB, a known PPARγ inhibitor [42].

The cell culture model chosen for the UV irradiation experiments had a significant influence on the level of proteins with antioxidant activity. In 2D cultured fibroblasts, the expression and activity of antioxidant enzymes (including catalase or superoxide dismutase), was found to be significantly decreased [7]. Such changes have not been widely observed in 3D cultured cells despite the penetrating nature of UV radiation. In our study, in all experiments, CBD significantly enhanced the level of enzymes related to the GSH metabolism (Table 1), such as γ-glutamylocysteine synthetase (γ-GCS GSH), GSH-S-transferase (GST), and glutathione peroxidase (GSH-Px). This cytoprotective action of CBD has been previously described in multiple pathological conditions [43]. Additionally, CBD treatment of 2D cultured cells supports an antioxidant system based on thioredoxin reduction abilities (Table 1), which has previously been implicated as a result of endocannabinoid system activation in the cholangiocytes mice [44].

The UV-induced decrease in antioxidant capacity leads to disturbances in the redox balance, resulting in an increase of the peroxidation processes, including lipid peroxidation [7]. As a result, lipid peroxidation products, including highly reactive aldehydes, are prone to creating adducts with proteins, thereby changing the protein function [45]. As we have shown in this study, smaller changes in the levels of proteins with antioxidant propertieswere observed in the 3D cultured fibroblasts following UV radiation, when compared with 2D cultured fibroblasts. These higher levels of antioxidant protein in 2D cultured cells are associated with the observed lower levels of protein adducts with lipid peroxidation products. 

Moreover, UV radiation favoured adduct formation on proteins involved in transport and molecular transduction. 4-HNE, one of the most common lipid peroxidation products capable of forming adducts [44], has been previously identified as an activator of transporter protein expression and activity stimulation. This is especially relevant in the case of membrane transporters, such as multidrug resistance protein-1 (MRP1), and cytosolic transferases, including glutathione S-transferase-M1 (GSTM1) [46]. Also, in this study, we showed that the most sensitive proteins to modifications by adduct formation with lipid peroxidation products were these transporters (Figure 6). However, the level of transporters modified by lipid peroxidation products was two times lower in UV irradiated cells following CBD treatment, suggesting that CBD significantly protects transporter proteins against modifications via preventing lipid peroxidation or affecting intracellular signalling pathways. Alternatively, CBD treatment leads to the modification of proteins with receptor regulatory activity. Receptor modification by 4-HNE may cause activation of this protein, as it has been previously shown in the case of PPAR β/δ [47]. Therefore, CBD treatment may not only alter the expression of PPAR receptors but also impact the activation of these receptors, contributing to antioxidant and anti-inflammatory cell reaction. In addition, lipid peroxidation products bonded to platelet-derived growth factor receptor (PDGFR) have been shown to be effective antagonists of this protein [48]. PDGFR is responsible for regulating cell growth, proliferation, and differentiation through MAPK and PI3K pathway activation [49]. Overexpression and high activity of MAPK and PI3K may also lead to the development of many diseases including cancer [50], therefore the CBD-induced inhibition of PDGFR activation by adduct formation with lipid peroxidation products may protect against increased expression and uncontrolled activation of PI3K, potentially prevent skin cell carcinogenesis under stress.

## 5. Conclusions

We have shown in this study the capacity of CBD to function as a cytoprotective agent against UV-induced changes in both 2D and 3D cultured fibroblasts. Overall, 2D cultured cells showed a greater number of differentially expressed proteins, but there were significant differences on the molecular functions of the altered proteins. CBD-treated 2D cultured cells exposed to UV-radiation exhibited a more marked effect on the levels of proteins involved with antioxidant response and inflammation. On the other hand, 3D-cultured fibroblasts showed a higher response to CBD action against UV induced changes, which were based on the activation of signalling pathways. Therefore, improved understanding of CBD action in a multilayer skin cell model subjected to UV irradiation has provided a new insight into the nature of fibroblast cell-cell interaction, as well as on the alteration of skin cell metabolism. Knowledge about the lower protective effect of CBD in 3D cultured fibroblasts should be taken into account during the design of UV light protection.

## Figures and Tables

**Table 1:** The list and biological/molecular functions of proteins that expression was significant changed in fibroblasts irradiated with UVA (20 J/cm^2^) and UVB (200 mJ/cm^2^) or/and treated with cannabidiol (CBD, 4 µM) in two-dimensional (2D) or three-dimensional (3D) culture model.

Changes	ID	Protein Name	Biological/Molecular Functions	Fold Change between Control and CBD Treated Cells		Fold Change between UVA Irradiated Cells and Irradiated Cells Treated with CBD		Fold Change between UVB Irradiated Cells and Irradiated Cells Treated with CBD	
				2D Cultures	3D Cultures	2D Cultures	3D Cultures	2D Cultures	3D Cultures
INCREASE	Q02388	collagen	structural	3.7	2.1	4.1	2.3	4.0	2.7
	H7C4K3	β1-integrin	structural	6.1		4.5		4.0	
	P17301	A2-integrin	structural	4.9		6.1		5.4	
	P60709	Actin	structural	3.3		4.3		4.0	
	P12882	Myosin-1	structural	3.6		3.9		3.5	
	Q14457	Beclin-1	structural	6.2		6.1		6.7	
	Q20679	2-oxoglutarate 5-dioxygenase 2	structural, collagen synthesis	5.1					
	Q9UL44	Lysyl hydroxylase 1	structural, collagen synthesis			3.8		2.7	
	Q9Y6D3	Lysyl hydroxylase 2	structural, collagen synthesis			4.3		3.1	
	Q86SQ4	Adhesion G-protein coupled receptor G6	structural, collagen binding	4.2					
	Q05397	Focal adhesion kinase 1 (FAK1)	catalytic activity, kinase	3.8	6.1	2.2	4.0	2.1	4.1
	Q14289	Focal adhesion kinase 2 (FAK2)	catalytic activity, kinase		4.1	3.1	5.7	3.7	5.4
	Q15759	p38	catalytic activity, kinase			6.4	8.1	6.1	9.0
	P31749	Serine/threonine-protein kinase AKT1	catalytic activity, kinase	3.2	4.5	3.3	6.1	3.3	5.4
	P31751	Serine/threonine-protein kinase AKT2	catalytic activity, kinase		4.2	3.6	4.3	3.4	4.0
	Q9H8T0	AKT-interacting protein (AktIP)	catalytic activity, kinase			2.4		2.3	
	P27986	Phosphoinositide 3-kinase (PI3K)	catalytic activity, kinase	4.1	4.0	3.1	5.4	3.7	5.1
	Q8IW41	MAP kinase-activated protein kinase 5 (MAPKAPK5)	catalytic activity, kinase			3.8		3.4	
	Q04206	NF-kappa-B-repressing factor (NKRF)	intracellular signaling, anti-inflammatory factor			4.3		3.9	
	P37231	Peroxisome proliferator-activated receptor γ (PPARγ)	intracellular signaling, antioxidant response			2.9	4.2	2.4	4.7
	Q03181	Peroxisome proliferator-activated receptor δ (PPARδ)	intracellular signaling, antioxidant response			3.4	5.1	3.7	5.2
	Q9UBK2	Peroxisome proliferator-activated receptor gamma coactivator 1A (PPARGC1A)	intracellular signaling, antioxidant response			2.6		4.9	
	D2KUA6	NR1C3 (PPARγ subunit)	intracellular signaling, antioxidant response			4.9		3.9	
	Q3C1U4	PPARγ-DNA-binding domain-interacting protein 1β (PDIP1β)	intracellular signaling, antioxidant response			3.3		3.1	
	P35669	γ-Glutamate cysteine ligase A1 (γ-GCS A1)	catalytic activity, antioxidant response	3.4		3.6			
	P22352	Glutathione peroxidase 3 (GSH -Px3)	catalytic activity, antioxidant response			6.2		3.7	2.0
	P07203	Glutathione peroxidase 1 (GSH -Px1)	catalytic activity, antioxidant response			5.1		4.8	2.2
	P08263	Glutathione S-transferase A1 (GSTA1)	catalytic activity, antioxidant response			3.2	2.1		
	P48637	Glutathione synthetase	catalytic activity, antioxidant response	3.2		3.8		4.4	
	V9HWJ1	Glutathione synthetase, HEL-S-64p	catalytic activity, antioxidant response			4.1		3.7	
	P48507	Glutamate-cysteine ligase	catalytic activity, antioxidant response	8.9					
	Q8IU80	Transmembrane protease serine 6	catalytic activity, protease	6.7					
	P04083	Annexin A1	structural, transport					4.2	
DECREASE	Q8NBJ5	Procollagen galactosyltransferase 1	catalytic activity, collagen metabolism	0.3	0.4	0.2		0.2	
	Q15582	Transforming growth factor-beta-induced protein ig-h3	structural, cell-collagen interactions	0.2					
	Q06828	Fibromodulin	structural	0.4					
	P16671	Platelet glycoprotein 4	structural, cell-collagen interactions	0.2	0.4	0.2	0.5	0.3	0.6
	O14495	Phospholipid phosphatase 3	catalytic activity, phosphatase	0.3		0.3		0.2	
	P45452	Metalloproteinase-13	catalytic activity, protein degradation	0.3	0.4				
	P14780	Metalloproteinase-9	catalytic activity, protein degradation	0.2	0.3	0.3	0.4	0.4	0.5
	Q12884	Prolyl endopeptidase FAP	catalytic activity, protein degradation	0.3		0.4		0.4	
	P25774	Cathepsin S	catalytic activity, protein degradation	0.2	0.2				
	O61565	I-kappa-B kinase (IKK)	catalytic activity, kinase, pro-inflammatory factor			0.2	0.5	0.2	0.5
	O14920	I-kappa-B kinase subunit B (IKKB)	catalytic activity, kinase, pro-inflammatory factor			0.4	0.6	0.3	
	O15111	I-kappa-B kinase subunit A (IKKA)	catalytic activity, kinase, pro-inflammatory factor			0.2	0.4	0.3	
	O88619	Nuclear factor NFκB (p65)	intracellular signaling, pro-inflammatory factor			0.3	0.5	0.2	0.4
	P19838	Nuclear factor NFκB (p105)	intracellular signaling, pro-inflammatory factor			0.3		0.3	0.4
	Q96P31	Nuclear factor NFκB1	intracellular signaling, pro-inflammatory factor			0.2		0.2	
	Q04206	NFκBID	intracellular signaling, pro-inflammatory factor			0.3		0.3	
	P27487	Dipeptidyl peptidase 4	catalytic activity, protein degradation	0.2	0.5	0.4	0.5	0.3	0.4
	P48736	Phosphatidylinositol 4,5-bisphosphate 3-kinase	catalytic activity, kinase					0.2	0.8
	Q15139	Serine/threonine-protein kinase	catalytic activity, kinase					0.3	0.6
	P55085	Proteinase-activated receptor 2	catalytic activity, protein degradation					0.4	
	P35354	Prostaglandin G/H synthase 2	catalytic activity, lipid metabolism					0.5	

## Supplementary Materials

The following are available online at https://www.mdpi.com/2073-4409/8/9/995/s1, Table S1: The list of proteins (ID and medium intensity) identified in each experimental group: control fibroblasts irradiated with UVA (20 J/cm^2^) and UVB (200 mJ/cm^2^) or/and treated with cannabidiol (CBD, 4 μM) in two-dimensional (2D) or three-dimensional (3D) culture model.
