# Supplementary material for: The Differences in the Proteome Profile of Cannabidiol-Treated Skin Fibroblasts following UVA or UVB Irradiation in 2D and 3D Cell Cultures
Source: Cells. 2019 Aug 28;8(9):995. doi: 10.3390/cells8090995 (PMC6770406; doi:10.3390/cells8090995)
Supplement: Supplementary file 1 [file cells-08-00995-s001.pdf]

Supplementary Table 1. The list of proteins (ID and medium intensity) identified in each experimental group: control fibroblasts irradiated with UVA (20 J/cm$^2$) and UVB (200 mJ/cm$^2$) or/and treated with cannabidiol (CBD, 4 μM) in two-dimensional (2D) or three-dimensional (3D) culture model.

| | 2D cultured fibroblasts | | | | | | 3D cultured fibroblasts | | | | | |
|---|---|---|---|---|---|---|---|---|---|---|---|---|
| ID | control | CBD | UVA | UVA+CBD | UVB | UVB+CBD | control | CBD | UVA | UVA+CBD | UVB | UVB+CBD |
| A0A024QZ64 | 2,64E+07 | 2,30E+07 | 5,54E+07 | 5,54E+07 | 3,43E+07 | 4,12E+07 | 2,40E+07 | 2,40E+07 | 2,40E+07 | 2,40E+07 | 2,40E+07 | 2,40E+07 |
| A0A024QZ98 | 2,09E+06 | 1,82E+06 | 4,39E+06 | 4,39E+06 | 2,72E+06 | 3,26E+06 | 1,90E+06 | 1,46E+06 | 2,92E+06 | 4,09E+06 | 2,63E+06 | 3,42E+06 |
| A0A024QZN9 | 1,39E+10 | 1,21E+10 | 2,91E+10 | 2,91E+10 | 1,80E+10 | 2,16E+10 | 1,26E+10 | 1,26E+10 | 1,26E+10 | 1,26E+10 | 1,26E+10 | 1,26E+10 |
| A0A024QZT0 | 1,39E+10 | 1,21E+10 | 2,91E+10 | 2,91E+10 | 1,80E+10 | 2,16E+10 | 1,26E+10 | 1,26E+10 | 1,26E+10 | 1,26E+10 | 1,26E+10 | 1,26E+10 |
| A0A024R2P0 | 2,09E+06 | 1,82E+06 | 4,39E+06 | 4,39E+06 | 2,72E+06 | 3,26E+06 | 1,90E+06 | 1,46E+06 | 2,92E+06 | 4,09E+06 | 2,63E+06 | 3,42E+06 |
| A0A024R454 | 4,84E+05 | 4,21E+05 | 1,02E+06 | 1,02E+06 | 6,29E+05 | 7,55E+05 | 4,40E+05 | 4,40E+05 | 4,40E+05 | 1,60E+06 | 2,60E+06 | 2,80E+06 |
| A0A024R5Z9 | 3,56E+08 | 3,10E+08 | 7,48E+08 | 7,48E+08 | 4,63E+08 | 5,56E+08 | 3,24E+08 | 3,24E+08 | 3,24E+08 | 3,24E+08 | 3,24E+08 | 3,24E+08 |
| A0A024R609 | 3,56E+08 | 3,10E+08 | 7,48E+08 | 7,48E+08 | 4,63E+08 | 5,56E+08 | 3,24E+08 | 3,24E+08 | 3,24E+08 | 3,24E+08 | 3,24E+08 | 3,24E+08 |
| A0A024R6I7 | 4,62E+06 | 4,02E+06 | 9,70E+06 | 9,70E+06 | 6,01E+06 | 7,21E+06 | 4,20E+06 | 3,23E+06 | 6,46E+06 | 9,05E+06 | 5,82E+06 | 7,56E+06 |
| A0A024R6W0 | 2,09E+06 | 1,82E+06 | 4,39E+06 | 4,39E+06 | 2,72E+06 | 3,26E+06 | 1,90E+06 | 1,46E+06 | 2,92E+06 | 4,09E+06 | 2,63E+06 | 3,42E+06 |
| A0A024R7H0 | 2,09E+06 | 1,82E+06 | 4,39E+06 | 4,39E+06 | 2,72E+06 | 3,26E+06 | 1,90E+06 | 1,90E+06 | 1,90E+06 | 1,90E+06 | 1,90E+06 | 1,90E+06 |
| A0A024R8S5 | 1,65E+06 | 1,43E+06 | 3,47E+06 | 3,47E+06 | 2,15E+06 | 2,57E+06 | 1,50E+06 | 1,50E+06 | 1,50E+06 | 1,50E+06 | 1,50E+06 | 1,50E+06 |
| A0A024R944 | 2,64E+04 | 2,30E+04 | 5,54E+04 | 5,54E+04 | 3,43E+04 | 4,12E+04 | 2,40E+04 | 2,40E+04 | 2,40E+04 | 2,40E+04 | 2,40E+04 | 2,40E+04 |
| A0A024RAZ7 | 2,09E+06 | 1,82E+06 | 4,39E+06 | 4,39E+06 | 2,72E+06 | 3,26E+06 | 1,90E+06 | 1,46E+06 | 2,92E+06 | 4,09E+06 | 2,63E+06 | 3,42E+06 |
| A0A024RB53 | 1,39E+10 | 1,21E+10 | 2,91E+10 | 2,91E+10 | 1,80E+10 | 2,16E+10 | 1,26E+10 | 9,69E+09 | 1,94E+10 | 2,71E+10 | 1,74E+10 | 2,27E+10 |
| A0A024RBS2 | 1,98E+06 | 1,72E+06 | 4,16E+06 | 4,16E+06 | 2,57E+06 | 3,09E+06 | 1,80E+06 | 1,38E+06 | 2,77E+06 | 3,88E+06 | 2,49E+06 | 3,24E+06 |
| A0A024RC61 | 1,65E+06 | 1,43E+06 | 3,47E+06 | 3,47E+06 | 2,15E+06 | 2,57E+06 | 1,50E+06 | 1,50E+06 | 1,50E+06 | 1,50E+06 | 1,50E+06 | 1,50E+06 |
| A0A087WUV8 | 4,95E+06 | 4,30E+06 | 1,04E+07 | 1,04E+07 | 6,44E+06 | 7,72E+06 | 4,50E+06 | 3,46E+06 | 6,92E+06 | 9,69E+06 | 6,23E+06 | 8,10E+06 |
| A0A087WV01 | 2,86E+06 | 2,49E+06 | 6,01E+06 | 6,01E+06 | 3,72E+06 | 4,46E+06 | 2,60E+06 | 2,60E+06 | 2,60E+06 | 6,80E+06 | 2,60E+06 | 2,60E+06 |
| A0A087WVQ9 | 2,86E+06 | 2,49E+06 | 6,01E+06 | 6,01E+06 | 3,72E+06 | 4,46E+06 | 2,60E+06 | 2,60E+06 | 2,60E+06 | 1,26E+10 | 2,60E+06 | 2,60E+06 |
| A0A087WZ27 | 2,64E+04 | 2,30E+04 | 5,54E+04 | 5,54E+04 | 3,43E+04 | 4,12E+04 | 2,40E+04 | 2,40E+04 | 2,40E+04 | 6,00E+06 | 2,40E+04 | 2,40E+04 |
| A0A087X1B9 | 2,64E+04 | 2,30E+04 | 5,54E+04 | 5,54E+04 | 3,43E+04 | 4,12E+04 | 2,40E+04 | 2,40E+04 | 2,40E+04 | 2,40E+04 | 2,40E+04 | 2,30E+06 |

| | | | | | | | | | | | | |
|---|---|---|---|---|---|---|---|---|---|---|---|---|
| A0A087X2B5 | 1,39E+10 | 1,21E+10 | 2,91E+10 | 2,91E+10 | 1,80E+10 | 2,16E+10 | 1,26E+10 | 9,69E+09 | 1,94E+10 | 2,71E+10 | 1,74E+10 | 2,27E+10 |
| A0A087X2E9 | 1,32E+06 | 1,15E+06 | 2,77E+06 | 2,77E+06 | 1,72E+06 | 2,06E+06 | 1,20E+06 | 1,20E+06 | 1,20E+06 | 5,90E+07 | 5,90E+07 | 5,90E+07 |
| A0A0A0MR02 | 1,39E+10 | 1,21E+10 | 2,91E+10 | 2,91E+10 | 1,80E+10 | 2,16E+10 | 1,26E+10 | 1,26E+10 | 1,26E+10 | 1,26E+10 | 1,26E+10 | 1,26E+10 |
| A0A0A0MSS8 | 2,09E+06 | 1,82E+06 | 4,39E+06 | 4,39E+06 | 2,72E+06 | 3,26E+06 | 1,90E+06 | 1,46E+06 | 2,92E+06 | 4,09E+06 | 2,63E+06 | 3,42E+06 |
| A0A0A0MT12 | 2,09E+06 | 1,82E+06 | 4,39E+06 | 4,39E+06 | 2,72E+06 | 3,26E+06 | 1,90E+06 | 1,90E+06 | 1,90E+06 | 1,90E+06 | 1,90E+06 | 1,90E+06 |
| A0A0A0MTS2 | 1,98E+06 | 1,72E+06 | 4,16E+06 | 4,16E+06 | 2,57E+06 | 3,09E+06 | 1,80E+06 | 1,38E+06 | 2,77E+06 | 3,88E+06 | 2,49E+06 | 3,24E+06 |
| A0A0C4DFU1 | 3,19E+07 | 2,77E+07 | 6,70E+07 | 6,70E+07 | 4,15E+07 | 4,98E+07 | 2,90E+07 | 2,90E+07 | 2,90E+07 | 2,90E+07 | 2,90E+07 | 2,90E+07 |
| A0A0C4DG17 | 3,63E+06 | 3,16E+06 | 7,62E+06 | 7,62E+06 | 4,72E+06 | 5,66E+06 | 3,30E+06 | 2,54E+06 | 5,08E+06 | 7,11E+06 | 4,57E+06 | 5,94E+06 |
| A0A0C4DG56 | 2,97E+07 | 2,58E+07 | 6,24E+07 | 6,24E+07 | 3,86E+07 | 4,63E+07 | 2,70E+07 | 2,70E+07 | 2,70E+07 | 2,70E+07 | 2,70E+07 | 2,70E+07 |
| A0A0C4DGC5 | 2,64E+07 | 2,30E+07 | 5,54E+07 | 5,54E+07 | 3,43E+07 | 4,12E+07 | 2,40E+07 | 2,40E+07 | 2,40E+07 | 2,40E+07 | 2,40E+07 | 2,40E+07 |
| A0A0G2JJL7 | 2,09E+06 | 1,82E+06 | 4,39E+06 | 4,39E+06 | 2,72E+06 | 3,26E+06 | 1,90E+06 | 1,90E+06 | 1,90E+06 | 1,90E+06 | 1,90E+06 | 1,90E+06 |
| A0A0G2JNQ3 | 1,98E+07 | 1,72E+07 | 4,16E+07 | 4,16E+07 | 2,57E+07 | 3,09E+07 | 1,80E+07 | 1,80E+07 | 1,80E+07 | 1,80E+07 | 1,80E+07 | 1,80E+07 |
| A0A0G2JPF8 | 1,98E+07 | 1,72E+07 | 4,16E+07 | 4,16E+07 | 2,57E+07 | 3,09E+07 | 1,80E+07 | 1,80E+07 | 1,80E+07 | 1,80E+07 | 1,80E+07 | 1,80E+07 |
| A0A0G2JRN3 | 4,62E+06 | 4,02E+06 | 9,70E+06 | 9,70E+06 | 6,01E+06 | 7,21E+06 | 4,20E+06 | 3,23E+06 | 6,46E+06 | 9,05E+06 | 5,82E+06 | 7,56E+06 |
| A0A0J9YX90 | 2,09E+06 | 1,82E+06 | 4,39E+06 | 4,39E+06 | 2,72E+06 | 3,26E+06 | 1,90E+06 | 1,46E+06 | 2,92E+06 | 4,09E+06 | 2,63E+06 | 3,42E+06 |
| A0A0J9YXP8 | 2,09E+06 | 1,82E+06 | 4,39E+06 | 4,39E+06 | 2,72E+06 | 3,26E+06 | 1,90E+06 | 1,46E+06 | 2,92E+06 | 4,09E+06 | 2,63E+06 | 3,42E+06 |
| A0A0J9YXZ5 | 2,86E+06 | 2,49E+06 | 6,01E+06 | 6,01E+06 | 3,72E+06 | 4,46E+06 | 2,60E+06 | 2,60E+06 | 2,60E+06 | 2,60E+06 | 2,60E+06 | 6,40E+06 |
| A0A0J9YYH3 | 2,09E+06 | 1,82E+06 | 4,39E+06 | 4,39E+06 | 2,72E+06 | 3,26E+06 | 1,90E+06 | 1,46E+06 | 2,92E+06 | 4,09E+06 | 2,63E+06 | 3,42E+06 |
| A0A0K0Q2Z1 | 2,64E+04 | 2,30E+04 | 5,54E+04 | 5,54E+04 | 3,43E+04 | 4,12E+04 | 2,40E+04 | 2,40E+04 | 2,40E+04 | 2,40E+04 | 2,40E+04 | 2,40E+04 |
| A0A0K2BMD8 | 6,49E+06 | 5,64E+06 | 1,36E+07 | 1,36E+07 | 8,44E+06 | 1,01E+07 | 5,90E+06 | 4,54E+06 | 9,08E+06 | 1,27E+07 | 8,17E+06 | 1,06E+07 |
| A0A0S2Z359 | 8,47E+06 | 7,37E+06 | 1,78E+07 | 1,78E+07 | 1,10E+07 | 1,32E+07 | 7,70E+06 | 7,70E+06 | 7,70E+06 | 7,70E+06 | 7,70E+06 | 7,70E+06 |
| A0A0S2Z3H3 | 8,47E+06 | 7,37E+06 | 1,78E+07 | 1,78E+07 | 1,10E+07 | 1,32E+07 | 7,70E+06 | 7,70E+06 | 7,70E+06 | 7,70E+06 | 7,70E+06 | 7,70E+06 |
| A0AUL6 | 3,63E+05 | 3,16E+05 | 7,62E+05 | 7,62E+05 | 4,72E+05 | 5,66E+05 | 3,30E+05 | 3,30E+05 | 3,30E+05 | 3,80E+07 | 3,80E+07 | 3,80E+07 |
| A1XP52 | 2,86E+06 | 2,49E+06 | 6,01E+06 | 6,01E+06 | 3,72E+06 | 4,46E+06 | 2,60E+06 | 2,60E+06 | 3,30E+06 | 3,30E+06 | 2,50E+06 | 2,50E+06 |
| A4D111 | 1,54E+07 | 1,34E+07 | 3,23E+07 | 3,23E+07 | 2,00E+07 | 2,40E+07 | 1,40E+07 | 1,40E+07 | 1,40E+07 | 1,40E+07 | 1,40E+07 | 1,40E+07 |
| A4QMW8 | 2,09E+07 | 1,82E+07 | 4,39E+07 | 4,39E+07 | 2,72E+07 | 3,26E+07 | 1,90E+07 | 1,60E+06 | 5,50E+06 | 1,90E+07 | 1,60E+06 | 5,50E+06 |
| A4QPB0 | 2,86E+06 | 2,49E+06 | 6,01E+06 | 6,01E+06 | 3,72E+06 | 4,46E+06 | 2,60E+06 | 2,60E+06 | 2,60E+06 | 2,60E+06 | 2,60E+06 | 6,40E+06 |
| A4UCS8 | 6,86E+07 | 5,97E+07 | 1,44E+08 | 1,44E+08 | 8,92E+07 | 1,07E+08 | 6,24E+07 | 6,24E+07 | 6,24E+07 | 6,24E+07 | 6,24E+07 | 6,24E+07 |
| A4UCT0 | 4,18E+07 | 3,63E+07 | 8,78E+07 | 8,78E+07 | 5,43E+07 | 6,52E+07 | 3,80E+07 | 3,80E+07 | 3,80E+07 | 3,80E+07 | 3,80E+07 | 3,80E+07 |

| | | | | | | | | | | | | |
|---|---|---|---|---|---|---|---|---|---|---|---|---|
| A4UJ43 | 4,62E+06 | 4,02E+06 | 9,70E+06 | 9,70E+06 | 6,01E+06 | 7,21E+06 | 4,20E+06 | 3,23E+06 | 6,46E+06 | 9,05E+06 | 5,82E+06 | 7,56E+06 |
| A6NFX8 | 2,86E+06 | 2,49E+06 | 6,01E+06 | 6,01E+06 | 3,72E+06 | 4,46E+06 | 2,60E+06 | 2,60E+06 | 2,60E+06 | 3,70E+06 | 2,60E+06 | 2,60E+06 |
| A7L8C5 | 4,95E+06 | 4,30E+06 | 1,04E+07 | 1,04E+07 | 6,44E+06 | 7,72E+06 | 4,50E+06 | 3,46E+06 | 6,92E+06 | 9,69E+06 | 6,23E+06 | 8,10E+06 |
| A7L8C6 | 2,86E+07 | 2,49E+07 | 6,01E+07 | 6,01E+07 | 3,72E+07 | 4,46E+07 | 2,60E+07 | 2,00E+07 | 4,00E+07 | 5,60E+07 | 3,60E+07 | 4,68E+07 |
| A8K088 | 4,62E+06 | 4,02E+06 | 9,70E+06 | 9,70E+06 | 6,01E+06 | 7,21E+06 | 4,20E+06 | 4,20E+06 | 4,20E+06 | 4,20E+06 | 4,20E+06 | 4,20E+06 |
| A8K2H9 | 1,65E+06 | 1,43E+06 | 3,47E+06 | 3,47E+06 | 2,15E+06 | 2,57E+06 | 1,50E+06 | 1,50E+06 | 1,50E+06 | 1,50E+06 | 1,50E+06 | 1,50E+06 |
| A8K482 | 2,09E+06 | 1,82E+06 | 4,39E+06 | 4,39E+06 | 2,72E+06 | 3,26E+06 | 1,90E+06 | 1,46E+06 | 2,92E+06 | 4,09E+06 | 2,63E+06 | 3,42E+06 |
| A8K486 | 1,03E+08 | 8,99E+07 | 2,17E+08 | 2,17E+08 | 1,34E+08 | 1,61E+08 | 9,40E+07 | 9,40E+07 | 9,40E+07 | 9,40E+07 | 9,40E+07 | 9,40E+07 |
| A8K4Y7 | 6,86E+07 | 5,97E+07 | 1,44E+08 | 1,44E+08 | 8,92E+07 | 1,07E+08 | 6,24E+07 | 6,24E+07 | 6,24E+07 | 6,24E+07 | 6,24E+07 | 6,24E+07 |
| A8K4Z4 | 2,09E+06 | 1,82E+06 | 4,39E+06 | 4,39E+06 | 2,72E+06 | 3,26E+06 | 1,90E+06 | 1,46E+06 | 2,92E+06 | 4,09E+06 | 2,63E+06 | 3,42E+06 |
| A8K674 | 2,09E+07 | 1,82E+07 | 4,39E+07 | 4,39E+07 | 2,72E+07 | 3,26E+07 | 1,90E+07 | 1,90E+07 | 1,90E+07 | 1,90E+07 | 1,90E+07 | 1,90E+07 |
| A8K787 | 3,19E+07 | 2,77E+07 | 6,70E+07 | 6,70E+07 | 4,15E+07 | 4,98E+07 | 2,90E+07 | 2,90E+07 | 2,90E+07 | 2,90E+07 | 2,90E+07 | 2,90E+07 |
| A8K7F6 | 7,92E+05 | 6,89E+05 | 1,66E+06 | 1,66E+06 | 1,03E+06 | 1,24E+06 | 7,20E+05 | 7,20E+05 | 7,20E+05 | 5,30E+06 | 5,30E+06 | 5,30E+06 |
| A8K7H3 | 5,76E+07 | 5,01E+07 | 1,21E+08 | 1,21E+08 | 7,49E+07 | 8,99E+07 | 5,24E+07 | 5,24E+07 | 5,24E+07 | 5,24E+07 | 5,24E+07 | 5,24E+07 |
| A8K9A4 | 3,85E+07 | 3,35E+07 | 8,09E+07 | 8,09E+07 | 5,01E+07 | 6,01E+07 | 3,50E+07 | 3,50E+07 | 3,50E+07 | 3,50E+07 | 3,50E+07 | 3,50E+07 |
| A8K9C4 | 2,86E+06 | 2,49E+06 | 6,01E+06 | 6,01E+06 | 3,72E+06 | 4,46E+06 | 2,60E+06 | 2,60E+06 | 2,60E+06 | 1,26E+10 | 2,60E+06 | 2,60E+06 |
| A8MVZ9 | 2,64E+07 | 2,30E+07 | 5,54E+07 | 5,54E+07 | 3,43E+07 | 4,12E+07 | 2,40E+07 | 2,40E+07 | 2,40E+07 | 2,40E+07 | 2,40E+07 | 2,40E+07 |
| A8MX94 | 2,09E+06 | 1,82E+06 | 4,39E+06 | 4,39E+06 | 2,72E+06 | 3,26E+06 | 1,90E+06 | 1,90E+06 | 1,90E+06 | 3,00E+07 | 3,00E+07 | 3,00E+07 |
| A8MYB8 | 1,98E+06 | 1,72E+06 | 4,16E+06 | 4,16E+06 | 2,57E+06 | 3,09E+06 | 1,80E+06 | 1,38E+06 | 2,77E+06 | 3,88E+06 | 2,49E+06 | 3,24E+06 |
| A9X7H1 | 2,86E+06 | 2,49E+06 | 6,01E+06 | 6,01E+06 | 3,72E+06 | 4,46E+06 | 2,60E+06 | 2,60E+06 | 2,60E+06 | 6,80E+06 | 2,60E+06 | 2,60E+06 |
| A9Z1X1 | 2,09E+06 | 1,82E+06 | 4,39E+06 | 4,39E+06 | 2,72E+06 | 3,26E+06 | 1,90E+06 | 1,46E+06 | 2,92E+06 | 4,09E+06 | 2,63E+06 | 3,42E+06 |
| B0AZS6 | 4,62E+06 | 4,02E+06 | 9,70E+06 | 9,70E+06 | 6,01E+06 | 7,21E+06 | 4,20E+06 | 4,20E+06 | 4,20E+06 | 1,40E+07 | 3,20E+06 | 2,60E+06 |
| B0YJC4 | 3,19E+07 | 2,77E+07 | 6,70E+07 | 6,70E+07 | 4,15E+07 | 4,98E+07 | 2,90E+07 | 2,90E+07 | 2,90E+07 | 2,90E+07 | 2,90E+07 | 2,90E+07 |
| B0YJC5 | 1,65E+06 | 1,43E+06 | 3,47E+06 | 3,47E+06 | 2,15E+06 | 2,57E+06 | 1,50E+06 | 1,50E+06 | 1,50E+06 | 1,50E+06 | 1,50E+06 | 1,50E+06 |
| B1Q3B3 | 2,46E+07 | 2,14E+07 | 5,17E+07 | 5,17E+07 | 3,20E+07 | 3,84E+07 | 2,24E+07 | 2,24E+07 | 2,24E+07 | 2,24E+07 | 2,24E+07 | 2,24E+07 |
| B2D098 | 1,39E+10 | 1,21E+10 | 2,91E+10 | 2,91E+10 | 1,80E+10 | 2,16E+10 | 1,26E+10 | 9,69E+09 | 1,94E+10 | 2,71E+10 | 1,74E+10 | 2,27E+10 |
| B2R4K7 | 9,46E+06 | 8,23E+06 | 1,99E+07 | 1,99E+07 | 1,23E+07 | 1,48E+07 | 8,60E+06 | 8,60E+06 | 8,60E+06 | 8,60E+06 | 8,60E+06 | 8,60E+06 |
| B2R4R0 | 1,43E+06 | 1,24E+06 | 3,00E+06 | 3,00E+06 | 1,86E+06 | 2,23E+06 | 1,30E+06 | 1,30E+06 | 1,30E+06 | 4,20E+08 | 5,20E+07 | 8,80E+06 |
| B2R5W2 | 3,85E+07 | 3,35E+07 | 8,09E+07 | 8,09E+07 | 5,01E+07 | 6,01E+07 | 3,50E+07 | 3,50E+07 | 3,50E+07 | 3,50E+07 | 3,50E+07 | 3,50E+07 |

| | | | | | | | | | | | | |
|---|---|---|---|---|---|---|---|---|---|---|---|---|
| B2R603 | 3,85E+07 | 3,35E+07 | 8,09E+07 | 8,09E+07 | 5,01E+07 | 6,01E+07 | 3,50E+07 | 3,50E+07 | 3,50E+07 | 3,50E+07 | 3,50E+07 | 3,50E+07 |
| B2R6Y1 | 2,09E+07 | 1,82E+07 | 4,39E+07 | 4,39E+07 | 2,72E+07 | 3,26E+07 | 1,90E+07 | 1,90E+07 | 1,90E+07 | 1,90E+07 | 1,90E+07 | 1,90E+07 |
| B2R983 | 2,42E+05 | 2,10E+05 | 5,08E+05 | 5,08E+05 | 3,15E+05 | 3,78E+05 | 2,20E+05 | 2,20E+05 | 2,20E+05 | 1,20E+07 | 1,80E+06 | 2,40E+04 |
| B2RD36 | 3,19E+07 | 2,77E+07 | 6,70E+07 | 6,70E+07 | 4,15E+07 | 4,98E+07 | 2,90E+07 | 2,90E+07 | 2,90E+07 | 2,90E+07 | 2,90E+07 | 2,90E+07 |
| B2RE56 | 1,03E+08 | 8,99E+07 | 2,17E+08 | 2,17E+08 | 1,34E+08 | 1,61E+08 | 9,40E+07 | 9,40E+07 | 9,40E+07 | 9,40E+07 | 9,40E+07 | 9,40E+07 |
| B2RXH8 | 1,98E+07 | 1,72E+07 | 4,16E+07 | 4,16E+07 | 2,57E+07 | 3,09E+07 | 1,80E+07 | 1,80E+07 | 1,80E+07 | 1,80E+07 | 1,80E+07 | 1,80E+07 |
| B3KM80 | 2,09E+07 | 1,82E+07 | 4,39E+07 | 4,39E+07 | 2,72E+07 | 3,26E+07 | 1,90E+07 | 1,90E+07 | 1,90E+07 | 1,90E+07 | 1,90E+07 | 1,90E+07 |
| B3KNB4 | 2,86E+06 | 2,49E+06 | 6,01E+06 | 6,01E+06 | 3,72E+06 | 4,46E+06 | 2,60E+06 | 2,60E+06 | 2,60E+06 | 1,80E+07 | 4,40E+06 | 2,60E+06 |
| B3KP88 | 2,86E+06 | 2,49E+06 | 6,01E+06 | 6,01E+06 | 3,72E+06 | 4,46E+06 | 2,60E+06 | 2,60E+06 | 2,60E+06 | 2,60E+06 | 2,60E+06 | 2,60E+06 |
| B3KPZ8 | 1,01E+07 | 8,80E+06 | 2,13E+07 | 2,13E+07 | 1,32E+07 | 1,58E+07 | 9,20E+06 | 9,20E+06 | 9,20E+06 | 1,70E+07 | 1,50E+06 | 3,30E+07 |
| B3KQT2 | 3,30E+06 | 2,87E+06 | 6,93E+06 | 6,93E+06 | 4,29E+06 | 5,15E+06 | 3,00E+06 | 3,00E+06 | 3,00E+06 | 6,60E+06 | 6,60E+06 | 6,60E+06 |
| B3KQT9 | 6,86E+07 | 5,97E+07 | 1,44E+08 | 1,44E+08 | 8,92E+07 | 1,07E+08 | 6,24E+07 | 6,24E+07 | 6,24E+07 | 6,24E+07 | 6,24E+07 | 6,24E+07 |
| B3KRK8 | 1,65E+06 | 1,43E+06 | 3,47E+06 | 3,47E+06 | 2,15E+06 | 2,57E+06 | 1,50E+06 | 1,50E+06 | 1,50E+06 | 1,50E+06 | 1,50E+06 | 1,50E+06 |
| B3KRY3 | 1,65E+06 | 1,43E+06 | 3,47E+06 | 3,47E+06 | 2,15E+06 | 2,57E+06 | 1,50E+06 | 1,50E+06 | 1,50E+06 | 1,50E+06 | 1,50E+06 | 1,50E+06 |
| B3KSJ6 | 3,63E+06 | 3,16E+06 | 7,62E+06 | 7,62E+06 | 4,72E+06 | 5,66E+06 | 3,30E+06 | 2,54E+06 | 5,08E+06 | 7,11E+06 | 4,57E+06 | 5,94E+06 |
| B3KSM6 | 3,08E+05 | 2,68E+05 | 6,47E+05 | 6,47E+05 | 4,00E+05 | 4,80E+05 | 2,80E+05 | 2,80E+05 | 2,80E+05 | 2,60E+06 | 4,60E+05 | 3,00E+06 |
| B3KT37 | 2,86E+06 | 2,49E+06 | 6,01E+06 | 6,01E+06 | 3,72E+06 | 4,46E+06 | 2,60E+06 | 2,60E+06 | 2,60E+06 | 1,26E+10 | 2,40E+06 | 2,26E+09 |
| B3KTM1 | 5,28E+05 | 4,59E+05 | 1,11E+06 | 1,11E+06 | 6,86E+05 | 8,24E+05 | 4,80E+05 | 4,80E+05 | 4,80E+05 | 2,90E+07 | 5,10E+06 | 1,20E+07 |
| B3KTM9 | 2,09E+06 | 1,82E+06 | 4,39E+06 | 4,39E+06 | 2,72E+06 | 3,26E+06 | 1,90E+06 | 1,46E+06 | 2,92E+06 | 4,09E+06 | 2,63E+06 | 3,42E+06 |
| B3KTN2 | 3,63E+06 | 3,16E+06 | 7,62E+06 | 7,62E+06 | 4,72E+06 | 5,66E+06 | 3,30E+06 | 2,54E+06 | 5,08E+06 | 7,11E+06 | 4,57E+06 | 5,94E+06 |
| B3KTP9 | 2,09E+07 | 1,82E+07 | 4,39E+07 | 4,39E+07 | 2,72E+07 | 3,26E+07 | 1,90E+07 | 1,90E+07 | 1,90E+07 | 1,90E+07 | 1,90E+07 | 1,90E+07 |
| B3KTS5 | 1,36E+09 | 1,19E+09 | 2,86E+09 | 2,86E+09 | 1,77E+09 | 2,13E+09 | 1,24E+09 | 1,24E+09 | 1,24E+09 | 1,24E+09 | 1,24E+09 | 1,24E+09 |
| B3KTT5 | 1,39E+10 | 1,21E+10 | 2,91E+10 | 2,91E+10 | 1,80E+10 | 2,16E+10 | 1,26E+10 | 9,69E+09 | 1,94E+10 | 2,71E+10 | 1,74E+10 | 2,27E+10 |
| B3KU93 | 2,09E+06 | 1,82E+06 | 4,39E+06 | 4,39E+06 | 2,72E+06 | 3,26E+06 | 1,90E+06 | 1,46E+06 | 2,92E+06 | 4,09E+06 | 2,63E+06 | 3,42E+06 |
| B3KUM3 | 2,09E+06 | 1,82E+06 | 4,39E+06 | 4,39E+06 | 2,72E+06 | 3,26E+06 | 1,90E+06 | 1,46E+06 | 2,92E+06 | 4,09E+06 | 2,63E+06 | 3,42E+06 |
| B3KUZ8 | 2,09E+06 | 1,82E+06 | 4,39E+06 | 4,39E+06 | 2,72E+06 | 3,26E+06 | 1,90E+06 | 1,46E+06 | 2,92E+06 | 4,09E+06 | 2,63E+06 | 3,42E+06 |
| B3KWQ3 | 7,92E+05 | 6,89E+05 | 1,66E+06 | 1,66E+06 | 1,03E+06 | 1,24E+06 | 7,20E+05 | 7,20E+05 | 7,20E+05 | 7,20E+05 | 7,20E+05 | 7,20E+05 |
| B3KX72 | 6,49E+06 | 5,64E+06 | 1,36E+07 | 1,36E+07 | 8,44E+06 | 1,01E+07 | 5,90E+06 | 4,54E+06 | 9,08E+06 | 1,27E+07 | 8,17E+06 | 1,06E+07 |
| B3KX96 | 3,19E+07 | 2,77E+07 | 6,70E+07 | 6,70E+07 | 4,15E+07 | 4,98E+07 | 2,90E+07 | 2,90E+07 | 2,90E+07 | 2,90E+07 | 2,90E+07 | 2,90E+07 |

| | | | | | | | | | | | | |
|---|---|---|---|---|---|---|---|---|---|---|---|---|
| B3KXC3 | 2,09E+06 | 1,82E+06 | 4,39E+06 | 4,39E+06 | 2,72E+06 | 3,26E+06 | 1,90E+06 | 1,46E+06 | 2,92E+06 | 4,09E+06 | 2,63E+06 | 3,42E+06 |
| B4DDF8 | 2,09E+06 | 1,82E+06 | 4,39E+06 | 4,39E+06 | 2,72E+06 | 3,26E+06 | 1,90E+06 | 1,46E+06 | 2,92E+06 | 4,09E+06 | 2,63E+06 | 3,42E+06 |
| B4DDM1 | 5,72E+06 | 4,97E+06 | 1,20E+07 | 1,20E+07 | 7,44E+06 | 8,92E+06 | 5,20E+06 | 5,20E+06 | 5,20E+06 | 5,20E+06 | 5,20E+06 | 5,20E+06 |
| B4DDM5 | 1,32E+06 | 1,15E+06 | 2,77E+06 | 2,77E+06 | 1,72E+06 | 2,06E+06 | 1,20E+06 | 1,20E+06 | 1,20E+06 | 1,20E+06 | 1,20E+06 | 2,40E+06 |
| B4DE36 | 2,09E+06 | 1,82E+06 | 4,39E+06 | 4,39E+06 | 2,72E+06 | 3,26E+06 | 1,90E+06 | 1,46E+06 | 2,92E+06 | 4,09E+06 | 2,63E+06 | 3,42E+06 |
| B4DEB1 | 2,09E+07 | 1,82E+07 | 4,39E+07 | 4,39E+07 | 2,72E+07 | 3,26E+07 | 1,90E+07 | 1,90E+07 | 1,90E+07 | 1,90E+07 | 1,90E+07 | 1,90E+07 |
| B4DEF7 | 1,65E+07 | 1,43E+07 | 3,47E+07 | 3,47E+07 | 2,15E+07 | 2,57E+07 | 1,50E+07 | 1,50E+07 | 1,50E+07 | 1,50E+07 | 1,50E+07 | 1,50E+07 |
| B4DEI3 | 1,36E+08 | 1,19E+08 | 2,86E+08 | 2,86E+08 | 1,77E+08 | 2,13E+08 | 1,24E+08 | 1,24E+08 | 1,24E+08 | 1,24E+08 | 1,24E+08 | 1,24E+08 |
| B4DFF1 | 1,32E+06 | 1,15E+06 | 2,77E+06 | 2,77E+06 | 1,72E+06 | 2,06E+06 | 1,20E+06 | 1,20E+06 | 1,20E+06 | 1,20E+06 | 1,20E+06 | 1,20E+06 |
| B4DFN9 | 3,08E+05 | 2,68E+05 | 6,47E+05 | 6,47E+05 | 4,00E+05 | 4,80E+05 | 2,80E+05 | 2,80E+05 | 2,80E+05 | 2,80E+05 | 4,60E+05 | 4,40E+06 |
| B4DFR3 | 1,36E+09 | 1,19E+09 | 2,86E+09 | 2,86E+09 | 1,77E+09 | 2,13E+09 | 1,24E+09 | 1,24E+09 | 1,24E+09 | 1,24E+09 | 1,24E+09 | 1,24E+09 |
| B4DGL0 | 1,65E+07 | 1,43E+07 | 3,47E+07 | 3,47E+07 | 2,15E+07 | 2,57E+07 | 1,50E+07 | 1,50E+07 | 1,50E+07 | 1,50E+07 | 1,50E+07 | 1,50E+07 |
| B4DGW3 | 6,16E+06 | 5,36E+06 | 1,29E+07 | 1,29E+07 | 8,01E+06 | 9,61E+06 | 5,60E+06 | 5,60E+06 | 5,60E+06 | 5,60E+06 | 5,60E+06 | 5,60E+06 |
| B4DH02 | 6,49E+06 | 5,64E+06 | 1,36E+07 | 1,36E+07 | 8,44E+06 | 1,01E+07 | 5,90E+06 | 4,54E+06 | 9,08E+06 | 1,27E+07 | 8,17E+06 | 1,06E+07 |
| B4DHB3 | 4,29E+07 | 3,73E+07 | 9,01E+07 | 9,01E+07 | 5,58E+07 | 6,69E+07 | 3,90E+07 | 3,90E+07 | 3,90E+07 | 3,90E+07 | 3,90E+07 | 3,90E+07 |
| B4DHC4 | 3,63E+06 | 3,16E+06 | 7,62E+06 | 7,62E+06 | 4,72E+06 | 5,66E+06 | 3,30E+06 | 2,54E+06 | 5,08E+06 | 7,11E+06 | 4,57E+06 | 5,94E+06 |
| B4DHP5 | 3,63E+06 | 3,16E+06 | 7,62E+06 | 7,62E+06 | 4,72E+06 | 5,66E+06 | 3,30E+06 | 2,54E+06 | 5,08E+06 | 7,11E+06 | 4,57E+06 | 5,94E+06 |
| B4DHR1 | 3,85E+06 | 3,35E+06 | 8,09E+06 | 8,09E+06 | 5,01E+06 | 6,01E+06 | 3,50E+06 | 3,50E+06 | 3,50E+06 | 3,50E+06 | 3,50E+06 | 3,50E+06 |
| B4DHT9 | 1,32E+07 | 1,15E+07 | 2,77E+07 | 2,77E+07 | 1,72E+07 | 2,06E+07 | 1,20E+07 | 1,20E+07 | 1,20E+07 | 1,20E+07 | 1,20E+07 | 1,20E+07 |
| B4DI39 | 3,08E+05 | 2,68E+05 | 6,47E+05 | 6,47E+05 | 4,00E+05 | 4,80E+05 | 2,80E+05 | 2,80E+05 | 2,80E+05 | 2,80E+05 | 4,60E+05 | 4,40E+06 |
| B4DI70 | 1,32E+06 | 1,15E+06 | 2,77E+06 | 2,77E+06 | 1,72E+06 | 2,06E+06 | 1,20E+06 | 1,20E+06 | 1,20E+06 | 1,20E+06 | 1,20E+06 | 1,20E+06 |
| B4DIZ3 | 4,62E+06 | 4,02E+06 | 9,70E+06 | 9,70E+06 | 6,01E+06 | 7,21E+06 | 4,20E+06 | 3,23E+06 | 6,46E+06 | 9,05E+06 | 5,82E+06 | 7,56E+06 |
| B4DJ98 | 9,06E+07 | 7,88E+07 | 1,90E+08 | 1,90E+08 | 1,18E+08 | 1,41E+08 | 8,24E+07 | 8,24E+07 | 8,24E+07 | 8,24E+07 | 8,24E+07 | 8,24E+07 |
| B4DJA4 | 4,62E+06 | 4,02E+06 | 9,70E+06 | 9,70E+06 | 6,01E+06 | 7,21E+06 | 4,20E+06 | 3,23E+06 | 6,46E+06 | 9,05E+06 | 5,82E+06 | 7,56E+06 |
| B4DJC8 | 2,09E+06 | 1,82E+06 | 4,39E+06 | 4,39E+06 | 2,72E+06 | 3,26E+06 | 1,90E+06 | 1,46E+06 | 2,92E+06 | 4,09E+06 | 2,63E+06 | 3,42E+06 |
| B4DJS0 | 2,20E+06 | 1,91E+06 | 4,62E+06 | 4,62E+06 | 2,86E+06 | 3,43E+06 | 2,00E+06 | 2,00E+06 | 2,00E+06 | 1,20E+06 | 1,20E+06 | 1,20E+06 |
| B4DKL5 | 2,86E+07 | 2,49E+07 | 6,01E+07 | 6,01E+07 | 3,72E+07 | 4,46E+07 | 2,60E+07 | 2,00E+07 | 4,00E+07 | 5,60E+07 | 3,60E+07 | 4,68E+07 |
| B4DKM5 | 1,39E+10 | 1,21E+10 | 2,91E+10 | 2,91E+10 | 1,80E+10 | 2,16E+10 | 1,26E+10 | 1,26E+10 | 1,26E+10 | 1,26E+10 | 1,26E+10 | 1,26E+10 |
| B4DKP9 | 6,16E+06 | 5,36E+06 | 1,29E+07 | 1,29E+07 | 8,01E+06 | 9,61E+06 | 5,60E+06 | 5,60E+06 | 5,60E+06 | 5,60E+06 | 5,60E+06 | 5,60E+06 |

| | | | | | | | | | | | | |
|---|---|---|---|---|---|---|---|---|---|---|---|---|
| B4DL19 | 1,54E+07 | 1,34E+07 | 3,23E+07 | 3,23E+07 | 2,00E+07 | 2,40E+07 | 1,40E+07 | 1,40E+07 | 1,40E+07 | 1,40E+07 | 1,40E+07 | 1,40E+07 |
| B4DL32 | 2,64E+04 | 2,30E+04 | 5,54E+04 | 5,54E+04 | 3,43E+04 | 4,12E+04 | 2,40E+04 | 2,40E+04 | 2,40E+04 | 2,40E+04 | 2,40E+04 | 1,30E+06 |
| B4DL86 | 3,63E+06 | 3,16E+06 | 7,62E+06 | 7,62E+06 | 4,72E+06 | 5,66E+06 | 3,30E+06 | 2,54E+06 | 5,08E+06 | 7,11E+06 | 4,57E+06 | 5,94E+06 |
| B4DLN6 | 1,65E+06 | 1,43E+06 | 3,47E+06 | 3,47E+06 | 2,15E+06 | 2,57E+06 | 1,50E+06 | 1,50E+06 | 1,50E+06 | 1,50E+06 | 1,50E+06 | 1,50E+06 |
| B4DLR3 | 6,89E+09 | 5,99E+09 | 1,45E+10 | 1,45E+10 | 8,95E+09 | 1,07E+10 | 6,26E+09 | 6,26E+09 | 6,26E+09 | 3,60E+06 | 2,60E+06 | 1,90E+07 |
| B4DM82 | 4,73E+07 | 4,11E+07 | 9,93E+07 | 9,93E+07 | 6,15E+07 | 7,38E+07 | 4,30E+07 | 4,30E+07 | 4,30E+07 | 4,30E+07 | 4,30E+07 | 4,30E+07 |
| B4DMA2 | 1,65E+07 | 1,43E+07 | 3,47E+07 | 3,47E+07 | 2,15E+07 | 2,57E+07 | 1,50E+07 | 1,50E+07 | 1,50E+07 | 1,50E+07 | 1,50E+07 | 1,50E+07 |
| B4DMJ1 | 3,85E+07 | 3,35E+07 | 8,09E+07 | 8,09E+07 | 5,01E+07 | 6,01E+07 | 3,50E+07 | 3,50E+07 | 3,50E+07 | 3,50E+07 | 3,50E+07 | 3,50E+07 |
| B4DMT8 | 1,65E+06 | 1,43E+06 | 3,47E+06 | 3,47E+06 | 2,15E+06 | 2,57E+06 | 1,50E+06 | 1,50E+06 | 1,50E+06 | 1,50E+06 | 1,50E+06 | 1,50E+06 |
| B4DMW9 | 2,09E+06 | 1,82E+06 | 4,39E+06 | 4,39E+06 | 2,72E+06 | 3,26E+06 | 1,90E+06 | 1,46E+06 | 2,92E+06 | 4,09E+06 | 2,63E+06 | 3,42E+06 |
| B4DN70 | 1,39E+10 | 1,21E+10 | 2,91E+10 | 2,91E+10 | 1,80E+10 | 2,16E+10 | 1,26E+10 | 9,69E+09 | 1,94E+10 | 2,71E+10 | 1,74E+10 | 2,27E+10 |
| B4DNE0 | 2,86E+06 | 2,49E+06 | 6,01E+06 | 6,01E+06 | 3,72E+06 | 4,46E+06 | 2,60E+06 | 2,60E+06 | 2,60E+06 | 1,40E+07 | 2,60E+06 | 2,60E+06 |
| B4DNE1 | 4,62E+06 | 4,02E+06 | 9,70E+06 | 9,70E+06 | 6,01E+06 | 7,21E+06 | 4,20E+06 | 3,23E+06 | 6,46E+06 | 9,05E+06 | 5,82E+06 | 7,56E+06 |
| B4DNH2 | 7,92E+05 | 6,89E+05 | 1,66E+06 | 1,66E+06 | 1,03E+06 | 1,24E+06 | 7,20E+05 | 7,20E+05 | 7,20E+05 | 5,30E+06 | 5,30E+06 | 5,30E+06 |
| B4DNL5 | 3,19E+07 | 2,77E+07 | 6,70E+07 | 6,70E+07 | 4,15E+07 | 4,98E+07 | 2,90E+07 | 2,90E+07 | 2,90E+07 | 2,90E+07 | 2,90E+07 | 2,90E+07 |
| B4DNT8 | 6,89E+09 | 5,99E+09 | 1,45E+10 | 1,45E+10 | 8,95E+09 | 1,07E+10 | 6,26E+09 | 6,26E+09 | 6,26E+09 | 3,60E+06 | 2,60E+06 | 1,90E+07 |
| B4DNV4 | 2,86E+06 | 2,49E+06 | 6,01E+06 | 6,01E+06 | 3,72E+06 | 4,46E+06 | 2,60E+06 | 2,60E+06 | 2,60E+06 | 2,60E+06 | 2,60E+06 | 2,60E+06 |
| B4DNX1 | 1,32E+06 | 1,15E+06 | 2,77E+06 | 2,77E+06 | 1,72E+06 | 2,06E+06 | 1,20E+06 | 1,20E+06 | 1,20E+06 | 1,20E+06 | 1,20E+06 | 1,20E+06 |
| B4DP35 | 1,39E+10 | 1,21E+10 | 2,91E+10 | 2,91E+10 | 1,80E+10 | 2,16E+10 | 1,26E+10 | 9,69E+09 | 1,94E+10 | 2,71E+10 | 1,74E+10 | 2,27E+10 |
| B4DPM0 | 4,29E+07 | 3,73E+07 | 9,01E+07 | 9,01E+07 | 5,58E+07 | 6,69E+07 | 3,90E+07 | 3,90E+07 | 3,90E+07 | 3,90E+07 | 3,90E+07 | 3,90E+07 |
| B4DRT3 | 3,56E+08 | 3,10E+08 | 7,48E+08 | 7,48E+08 | 4,63E+08 | 5,56E+08 | 3,24E+08 | 3,24E+08 | 3,24E+08 | 3,24E+08 | 3,24E+08 | 3,24E+08 |
| B4DRV9 | 2,46E+07 | 2,14E+07 | 5,17E+07 | 5,17E+07 | 3,20E+07 | 3,84E+07 | 2,24E+07 | 2,24E+07 | 2,24E+07 | 2,24E+07 | 2,24E+07 | 2,24E+07 |
| B4DRX3 | 2,64E+04 | 2,30E+04 | 5,54E+04 | 5,54E+04 | 3,43E+04 | 4,12E+04 | 2,40E+04 | 2,40E+04 | 2,40E+04 | 2,40E+04 | 2,40E+04 | 2,40E+04 |
| B4DSU6 | 3,85E+07 | 3,35E+07 | 8,09E+07 | 8,09E+07 | 5,01E+07 | 6,01E+07 | 3,50E+07 | 3,50E+07 | 3,50E+07 | 3,50E+07 | 3,50E+07 | 3,50E+07 |
| B4DTC3 | 3,63E+06 | 3,16E+06 | 7,62E+06 | 7,62E+06 | 4,72E+06 | 5,66E+06 | 3,30E+06 | 3,30E+06 | 3,30E+06 | 2,60E+07 | 6,30E+06 | 2,60E+06 |
| B4DTX2 | 1,54E+07 | 1,34E+07 | 3,23E+07 | 3,23E+07 | 2,00E+07 | 2,40E+07 | 1,40E+07 | 1,40E+07 | 1,40E+07 | 1,40E+07 | 1,40E+07 | 1,40E+07 |
| B4DU71 | 1,32E+07 | 1,15E+07 | 2,77E+07 | 2,77E+07 | 1,72E+07 | 2,06E+07 | 1,20E+07 | 1,20E+07 | 1,20E+07 | 1,20E+07 | 1,20E+07 | 1,20E+07 |
| B4DUA5 | 1,65E+06 | 1,43E+06 | 3,47E+06 | 3,47E+06 | 2,15E+06 | 2,57E+06 | 1,50E+06 | 1,50E+06 | 1,50E+06 | 1,50E+06 | 1,50E+06 | 1,50E+06 |
| B4DUK1 | 2,09E+06 | 1,82E+06 | 4,39E+06 | 4,39E+06 | 2,72E+06 | 3,26E+06 | 1,90E+06 | 1,46E+06 | 2,92E+06 | 4,09E+06 | 2,63E+06 | 3,42E+06 |

| | | | | | | | | | | | | |
|---|---|---|---|---|---|---|---|---|---|---|---|---|
| B4DUK7 | 1,43E+07 | 1,24E+07 | 3,00E+07 | 3,00E+07 | 1,86E+07 | 2,23E+07 | 1,30E+07 | 1,30E+07 | 1,30E+07 | 1,30E+07 | 1,30E+07 | 1,30E+07 |
| B4DUP0 | 2,46E+07 | 2,14E+07 | 5,17E+07 | 5,17E+07 | 3,20E+07 | 3,84E+07 | 2,24E+07 | 2,24E+07 | 2,24E+07 | 2,24E+07 | 2,24E+07 | 2,24E+07 |
| B4DUS3 | 1,98E+06 | 1,72E+06 | 4,16E+06 | 4,16E+06 | 2,57E+06 | 3,09E+06 | 1,80E+06 | 1,38E+06 | 2,77E+06 | 3,88E+06 | 2,49E+06 | 3,24E+06 |
| B4DV12 | 2,09E+07 | 1,82E+07 | 4,39E+07 | 4,39E+07 | 2,72E+07 | 3,26E+07 | 1,90E+07 | 1,90E+07 | 1,90E+07 | 1,90E+07 | 1,90E+07 | 1,90E+07 |
| B4DV28 | 6,89E+09 | 5,99E+09 | 1,45E+10 | 1,45E+10 | 8,95E+09 | 1,07E+10 | 6,26E+09 | 6,26E+09 | 6,26E+09 | 3,60E+06 | 2,60E+06 | 1,90E+07 |
| B4DV42 | 2,86E+06 | 2,49E+06 | 6,01E+06 | 6,01E+06 | 3,72E+06 | 4,46E+06 | 2,60E+06 | 2,60E+06 | 2,60E+06 | 1,26E+10 | 2,60E+06 | 2,60E+06 |
| B4DV68 | 4,95E+06 | 4,30E+06 | 1,04E+07 | 1,04E+07 | 6,44E+06 | 7,72E+06 | 4,50E+06 | 3,46E+06 | 6,92E+06 | 9,69E+06 | 6,23E+06 | 8,10E+06 |
| B4DVE1 | 1,32E+06 | 1,15E+06 | 2,77E+06 | 2,77E+06 | 1,72E+06 | 2,06E+06 | 1,20E+06 | 1,20E+06 | 1,20E+06 | 1,20E+06 | 1,20E+06 | 1,20E+06 |
| B4DVJ0 | 2,09E+06 | 1,82E+06 | 4,39E+06 | 4,39E+06 | 2,72E+06 | 3,26E+06 | 1,90E+06 | 1,46E+06 | 2,92E+06 | 4,09E+06 | 2,63E+06 | 3,42E+06 |
| B4DVU1 | 8,25E+06 | 7,17E+06 | 1,73E+07 | 1,73E+07 | 1,07E+07 | 1,29E+07 | 7,50E+06 | 7,50E+06 | 7,50E+06 | 1,70E+07 | 1,50E+06 | 3,30E+07 |
| B4DVU9 | 3,08E+05 | 2,68E+05 | 6,47E+05 | 6,47E+05 | 4,00E+05 | 4,80E+05 | 2,80E+05 | 2,80E+05 | 2,80E+05 | 2,80E+05 | 4,60E+05 | 4,40E+06 |
| B4DW05 | 6,86E+07 | 5,97E+07 | 1,44E+08 | 1,44E+08 | 8,92E+07 | 1,07E+08 | 6,24E+07 | 6,24E+07 | 6,24E+07 | 6,24E+07 | 6,24E+07 | 6,24E+07 |
| B4DW52 | 2,97E+06 | 2,58E+06 | 6,24E+06 | 6,24E+06 | 3,86E+06 | 4,63E+06 | 2,70E+06 | 2,70E+06 | 2,70E+06 | 4,90E+07 | 4,90E+07 | 4,90E+07 |
| B4DWA8 | 1,32E+06 | 1,15E+06 | 2,77E+06 | 2,77E+06 | 1,72E+06 | 2,06E+06 | 1,20E+06 | 1,20E+06 | 1,20E+06 | 1,20E+06 | 1,20E+06 | 1,20E+06 |
| B4DX78 | 3,63E+06 | 3,16E+06 | 7,62E+06 | 7,62E+06 | 4,72E+06 | 5,66E+06 | 3,30E+06 | 3,30E+06 | 3,30E+06 | 3,30E+06 | 3,30E+06 | 3,30E+06 |
| B4DXX3 | 2,09E+06 | 1,82E+06 | 4,39E+06 | 4,39E+06 | 2,72E+06 | 3,26E+06 | 1,90E+06 | 1,46E+06 | 2,92E+06 | 4,09E+06 | 2,63E+06 | 3,42E+06 |
| B4DY08 | 3,85E+07 | 3,35E+07 | 8,09E+07 | 8,09E+07 | 5,01E+07 | 6,01E+07 | 3,50E+07 | 3,50E+07 | 3,50E+07 | 3,50E+07 | 3,50E+07 | 3,50E+07 |
| B4E0B5 | 3,63E+06 | 3,16E+06 | 7,62E+06 | 7,62E+06 | 4,72E+06 | 5,66E+06 | 3,30E+06 | 2,54E+06 | 5,08E+06 | 7,11E+06 | 4,57E+06 | 5,94E+06 |
| B4E1S9 | 3,08E+05 | 2,68E+05 | 6,47E+05 | 6,47E+05 | 4,00E+05 | 4,80E+05 | 2,80E+05 | 2,80E+05 | 2,80E+05 | 2,80E+05 | 4,60E+05 | 4,40E+06 |
| B4E1T1 | 2,64E+04 | 2,30E+04 | 5,54E+04 | 5,54E+04 | 3,43E+04 | 4,12E+04 | 2,40E+04 | 2,40E+04 | 2,40E+04 | 2,40E+04 | 2,40E+04 | 1,30E+06 |
| B4E1T6 | 3,63E+06 | 3,16E+06 | 7,62E+06 | 7,62E+06 | 4,72E+06 | 5,66E+06 | 3,30E+06 | 3,30E+06 | 3,30E+06 | 2,60E+07 | 6,30E+06 | 2,60E+06 |
| B4E2J2 | 2,09E+06 | 1,82E+06 | 4,39E+06 | 4,39E+06 | 2,72E+06 | 3,26E+06 | 1,90E+06 | 1,46E+06 | 2,92E+06 | 4,09E+06 | 2,63E+06 | 3,42E+06 |
| B4E2S7 | 1,65E+06 | 1,43E+06 | 3,47E+06 | 3,47E+06 | 2,15E+06 | 2,57E+06 | 1,50E+06 | 1,50E+06 | 1,50E+06 | 1,50E+06 | 1,50E+06 | 1,50E+06 |
| B4E2U0 | 3,63E+06 | 3,16E+06 | 7,62E+06 | 7,62E+06 | 4,72E+06 | 5,66E+06 | 3,30E+06 | 2,54E+06 | 5,08E+06 | 7,11E+06 | 4,57E+06 | 5,94E+06 |
| B4E380 | 2,09E+07 | 1,82E+07 | 4,39E+07 | 4,39E+07 | 2,72E+07 | 3,26E+07 | 1,90E+07 | 1,90E+07 | 1,90E+07 | 1,90E+07 | 1,90E+07 | 1,90E+07 |
| B4E3A8 | 3,63E+06 | 3,16E+06 | 7,62E+06 | 7,62E+06 | 4,72E+06 | 5,66E+06 | 3,30E+06 | 3,30E+06 | 3,30E+06 | 2,60E+07 | 6,30E+06 | 2,60E+06 |
| B4E3D5 | 2,09E+06 | 1,82E+06 | 4,39E+06 | 4,39E+06 | 2,72E+06 | 3,26E+06 | 1,90E+06 | 1,46E+06 | 2,92E+06 | 4,09E+06 | 2,63E+06 | 3,42E+06 |
| B5BU38 | 1,54E+06 | 1,34E+06 | 3,23E+06 | 3,23E+06 | 2,00E+06 | 2,40E+06 | 1,40E+06 | 1,40E+06 | 1,40E+06 | 1,50E+07 | 9,60E+05 | 2,20E+06 |
| B5MC07 | 1,98E+06 | 1,72E+06 | 4,16E+06 | 4,16E+06 | 2,57E+06 | 3,09E+06 | 1,80E+06 | 1,38E+06 | 2,77E+06 | 3,88E+06 | 2,49E+06 | 3,24E+06 |

| | | | | | | | | | | | | |
|---|---|---|---|---|---|---|---|---|---|---|---|---|
| B5MDF5 | 2,42E+07 | 2,10E+07 | 5,08E+07 | 5,08E+07 | 3,15E+07 | 3,78E+07 | 2,20E+07 | 2,30E+06 | 2,00E+06 | 2,20E+07 | 2,30E+06 | 2,00E+06 |
| B7Z1I2 | 3,63E+06 | 3,16E+06 | 7,62E+06 | 7,62E+06 | 4,72E+06 | 5,66E+06 | 3,30E+06 | 2,54E+06 | 5,08E+06 | 7,11E+06 | 4,57E+06 | 5,94E+06 |
| B7Z1N6 | 2,64E+07 | 2,30E+07 | 5,54E+07 | 5,54E+07 | 3,43E+07 | 4,12E+07 | 2,40E+07 | 2,40E+07 | 2,40E+07 | 2,40E+07 | 2,40E+07 | 2,40E+07 |
| B7Z1V7 | 2,09E+06 | 1,82E+06 | 4,39E+06 | 4,39E+06 | 2,72E+06 | 3,26E+06 | 1,90E+06 | 1,46E+06 | 2,92E+06 | 4,09E+06 | 2,63E+06 | 3,42E+06 |
| B7Z1V9 | 1,32E+06 | 1,15E+06 | 2,77E+06 | 2,77E+06 | 1,72E+06 | 2,06E+06 | 1,20E+06 | 1,20E+06 | 1,20E+06 | 1,20E+06 | 1,20E+06 | 1,40E+06 |
| B7Z1Y2 | 2,64E+07 | 2,30E+07 | 5,54E+07 | 5,54E+07 | 3,43E+07 | 4,12E+07 | 2,40E+07 | 2,40E+07 | 2,40E+07 | 2,40E+07 | 2,40E+07 | 2,40E+07 |
| B7Z2E6 | 4,62E+06 | 4,02E+06 | 9,70E+06 | 9,70E+06 | 6,01E+06 | 7,21E+06 | 4,20E+06 | 3,23E+06 | 6,46E+06 | 9,05E+06 | 5,82E+06 | 7,56E+06 |
| B7Z2R9 | 3,19E+07 | 2,77E+07 | 6,70E+07 | 6,70E+07 | 4,15E+07 | 4,98E+07 | 2,90E+07 | 2,90E+07 | 2,90E+07 | 2,90E+07 | 2,90E+07 | 2,90E+07 |
| B7Z3K9 | 2,64E+07 | 2,30E+07 | 5,54E+07 | 5,54E+07 | 3,43E+07 | 4,12E+07 | 2,40E+07 | 2,40E+07 | 2,40E+07 | 2,40E+07 | 2,40E+07 | 2,40E+07 |
| B7Z4T3 | 1,39E+10 | 1,21E+10 | 2,91E+10 | 2,91E+10 | 1,80E+10 | 2,16E+10 | 1,26E+10 | 9,69E+09 | 1,94E+10 | 2,71E+10 | 1,74E+10 | 2,27E+10 |
| B7Z4V2 | 1,39E+10 | 1,21E+10 | 2,91E+10 | 2,91E+10 | 1,80E+10 | 2,16E+10 | 1,26E+10 | 9,69E+09 | 1,94E+10 | 2,71E+10 | 1,74E+10 | 2,27E+10 |
| B7Z6P1 | 1,32E+06 | 1,15E+06 | 2,77E+06 | 2,77E+06 | 1,72E+06 | 2,06E+06 | 1,20E+06 | 1,20E+06 | 1,20E+06 | 1,20E+06 | 1,20E+06 | 1,20E+06 |
| B7ZA86 | 2,09E+07 | 1,82E+07 | 4,39E+07 | 4,39E+07 | 2,72E+07 | 3,26E+07 | 1,90E+07 | 1,90E+07 | 1,90E+07 | 1,90E+07 | 1,90E+07 | 1,90E+07 |
| B7ZAP6 | 6,16E+06 | 5,36E+06 | 1,29E+07 | 1,29E+07 | 8,01E+06 | 9,61E+06 | 5,60E+06 | 5,60E+06 | 5,60E+06 | 5,60E+06 | 5,60E+06 | 5,60E+06 |
| B8ZZ54 | 3,63E+06 | 3,16E+06 | 7,62E+06 | 7,62E+06 | 4,72E+06 | 5,66E+06 | 3,30E+06 | 2,54E+06 | 5,08E+06 | 7,11E+06 | 4,57E+06 | 5,94E+06 |
| B8ZZL8 | 1,39E+10 | 1,21E+10 | 2,91E+10 | 2,91E+10 | 1,80E+10 | 2,16E+10 | 1,26E+10 | 9,69E+09 | 1,94E+10 | 2,71E+10 | 1,74E+10 | 2,27E+10 |
| B9VP19 | 4,62E+06 | 4,02E+06 | 9,70E+06 | 9,70E+06 | 6,01E+06 | 7,21E+06 | 4,20E+06 | 4,20E+06 | 4,20E+06 | 4,20E+06 | 4,20E+06 | 4,20E+06 |
| B9VPB4 | 7,92E+05 | 6,89E+05 | 1,66E+06 | 1,66E+06 | 1,03E+06 | 1,24E+06 | 7,20E+05 | 7,20E+05 | 7,20E+05 | 7,20E+05 | 7,20E+05 | 7,20E+05 |
| C1KH65 | 4,62E+06 | 4,02E+06 | 9,70E+06 | 9,70E+06 | 6,01E+06 | 7,21E+06 | 4,20E+06 | 3,23E+06 | 6,46E+06 | 9,05E+06 | 5,82E+06 | 7,56E+06 |
| C7DJS1 | 1,54E+07 | 1,34E+07 | 3,23E+07 | 3,23E+07 | 2,00E+07 | 2,40E+07 | 1,40E+07 | 1,40E+07 | 1,40E+07 | 1,40E+07 | 1,40E+07 | 1,40E+07 |
| C7DJS2 | 1,54E+07 | 1,34E+07 | 3,23E+07 | 3,23E+07 | 2,00E+07 | 2,40E+07 | 1,40E+07 | 1,40E+07 | 1,40E+07 | 1,40E+07 | 1,40E+07 | 1,40E+07 |
| C9J3E2 | 6,49E+06 | 5,64E+06 | 1,36E+07 | 1,36E+07 | 8,44E+06 | 1,01E+07 | 5,90E+06 | 4,54E+06 | 9,08E+06 | 1,27E+07 | 8,17E+06 | 1,06E+07 |
| C9J5S7 | 9,90E+07 | 8,61E+07 | 2,08E+08 | 2,08E+08 | 1,29E+08 | 1,54E+08 | 9,00E+07 | 9,00E+07 | 9,00E+07 | 9,00E+07 | 9,00E+07 | 9,00E+07 |
| C9J8F3 | 2,64E+07 | 2,30E+07 | 5,54E+07 | 5,54E+07 | 3,43E+07 | 4,12E+07 | 2,40E+07 | 2,40E+07 | 2,40E+07 | 2,40E+07 | 2,40E+07 | 2,40E+07 |
| C9J9K3 | 2,09E+06 | 1,82E+06 | 4,39E+06 | 4,39E+06 | 2,72E+06 | 3,26E+06 | 1,90E+06 | 1,46E+06 | 2,92E+06 | 4,09E+06 | 2,63E+06 | 3,42E+06 |
| C9JH19 | 2,86E+06 | 2,49E+06 | 6,01E+06 | 6,01E+06 | 3,72E+06 | 4,46E+06 | 2,60E+06 | 2,60E+06 | 6,00E+06 | 6,00E+06 | 1,60E+06 | 1,60E+06 |
| C9JKT2 | 6,49E+06 | 5,64E+06 | 1,36E+07 | 1,36E+07 | 8,44E+06 | 1,01E+07 | 5,90E+06 | 4,54E+06 | 9,08E+06 | 1,27E+07 | 8,17E+06 | 1,06E+07 |
| C9JMC5 | 1,39E+10 | 1,21E+10 | 2,91E+10 | 2,91E+10 | 1,80E+10 | 2,16E+10 | 1,26E+10 | 9,69E+09 | 1,94E+10 | 2,71E+10 | 1,74E+10 | 2,27E+10 |
| C9JRZ8 | 2,09E+06 | 1,82E+06 | 4,39E+06 | 4,39E+06 | 2,72E+06 | 3,26E+06 | 1,90E+06 | 1,46E+06 | 2,92E+06 | 4,09E+06 | 2,63E+06 | 3,42E+06 |

| | | | | | | | | | | | | |
|---|---|---|---|---|---|---|---|---|---|---|---|---|
| C9JV77 | 2,86E+06 | 2,49E+06 | 6,01E+06 | 6,01E+06 | 3,72E+06 | 4,46E+06 | 2,60E+06 | 2,60E+06 | 2,60E+06 | 2,60E+06 | 7,30E+05 | 2,60E+06 |
| C9K028 | 6,86E+07 | 5,97E+07 | 1,44E+08 | 1,44E+08 | 8,92E+07 | 1,07E+08 | 6,24E+07 | 6,24E+07 | 6,24E+07 | 6,24E+07 | 6,24E+07 | 6,24E+07 |
| D1MGQ2 | 2,86E+06 | 2,49E+06 | 6,01E+06 | 6,01E+06 | 3,72E+06 | 4,46E+06 | 2,60E+06 | 2,60E+06 | 2,60E+06 | 2,60E+06 | 2,60E+06 | 2,60E+06 |
| D2KUA6 | 6,10E+07 | 6,10E+07 | 8,30E+06 | 2,49E+07 | 3,80E+06 | 1,14E+07 | 1,40E+07 | 1,40E+07 | 1,40E+07 | 1,40E+07 | 1,40E+07 | 1,40E+07 |
| D6RBL5 | 2,86E+07 | 2,49E+07 | 6,01E+07 | 6,01E+07 | 3,72E+07 | 4,46E+07 | 2,60E+07 | 2,60E+07 | 2,60E+07 | 2,60E+07 | 2,60E+07 | 2,60E+07 |
| E5RHP0 | 6,86E+07 | 5,97E+07 | 1,44E+08 | 1,44E+08 | 8,92E+07 | 1,07E+08 | 6,24E+07 | 6,24E+07 | 6,24E+07 | 6,24E+07 | 6,24E+07 | 6,24E+07 |
| E7EQG2 | 7,92E+05 | 6,89E+05 | 1,66E+06 | 1,66E+06 | 1,03E+06 | 1,24E+06 | 7,20E+05 | 7,20E+05 | 7,20E+05 | 5,30E+06 | 5,30E+06 | 5,30E+06 |
| E7ERL0 | 2,09E+07 | 1,82E+07 | 4,39E+07 | 4,39E+07 | 2,72E+07 | 3,26E+07 | 1,90E+07 | 1,90E+07 | 1,90E+07 | 1,90E+07 | 1,90E+07 | 1,90E+07 |
| E7ESK7 | 4,62E+06 | 4,02E+06 | 9,70E+06 | 9,70E+06 | 6,01E+06 | 7,21E+06 | 4,20E+06 | 4,20E+06 | 4,20E+06 | 1,26E+10 | 2,60E+06 | 9,70E+05 |
| E7EX29 | 4,62E+06 | 4,02E+06 | 9,70E+06 | 9,70E+06 | 6,01E+06 | 7,21E+06 | 4,20E+06 | 4,20E+06 | 4,20E+06 | 1,26E+10 | 2,60E+06 | 9,70E+05 |
| E9PCX2 | 7,70E+06 | 6,70E+06 | 1,62E+07 | 1,62E+07 | 1,00E+07 | 1,20E+07 | 7,00E+06 | 7,00E+06 | 7,00E+06 | 7,00E+06 | 7,00E+06 | 7,00E+06 |
| E9PG15 | 1,65E+06 | 1,43E+06 | 3,47E+06 | 3,47E+06 | 2,15E+06 | 2,57E+06 | 1,50E+06 | 1,50E+06 | 1,50E+06 | 1,50E+06 | 1,50E+06 | 1,50E+06 |
| E9PI65 | 1,54E+07 | 1,34E+07 | 3,23E+07 | 3,23E+07 | 2,00E+07 | 2,40E+07 | 1,40E+07 | 1,40E+07 | 1,40E+07 | 1,40E+07 | 1,40E+07 | 1,40E+07 |
| E9PK25 | 3,96E+07 | 3,44E+07 | 8,32E+07 | 8,32E+07 | 5,15E+07 | 6,18E+07 | 3,60E+07 | 3,60E+07 | 3,60E+07 | 3,60E+07 | 3,60E+07 | 3,60E+07 |
| E9PK54 | 1,54E+07 | 1,34E+07 | 3,23E+07 | 3,23E+07 | 2,00E+07 | 2,40E+07 | 1,40E+07 | 1,40E+07 | 1,40E+07 | 1,40E+07 | 1,40E+07 | 1,40E+07 |
| E9PLF4 | 1,54E+07 | 1,34E+07 | 3,23E+07 | 3,23E+07 | 2,00E+07 | 2,40E+07 | 1,40E+07 | 1,40E+07 | 1,40E+07 | 1,40E+07 | 1,40E+07 | 1,40E+07 |
| E9PNN6 | 6,49E+06 | 5,64E+06 | 1,36E+07 | 1,36E+07 | 8,44E+06 | 1,01E+07 | 5,90E+06 | 4,54E+06 | 9,08E+06 | 1,27E+07 | 8,17E+06 | 1,06E+07 |
| E9PP50 | 2,64E+08 | 2,30E+08 | 5,54E+08 | 5,54E+08 | 3,43E+08 | 4,12E+08 | 2,40E+08 | 2,40E+08 | 2,40E+08 | 2,40E+08 | 2,40E+08 | 2,40E+08 |
| E9PPQ4 | 6,49E+06 | 5,64E+06 | 1,36E+07 | 1,36E+07 | 8,44E+06 | 1,01E+07 | 5,90E+06 | 4,54E+06 | 9,08E+06 | 1,27E+07 | 8,17E+06 | 1,06E+07 |
| E9PQB7 | 3,19E+07 | 2,77E+07 | 6,70E+07 | 6,70E+07 | 4,15E+07 | 4,98E+07 | 2,90E+07 | 2,90E+07 | 2,90E+07 | 2,90E+07 | 2,90E+07 | 2,90E+07 |
| E9PQK7 | 1,54E+07 | 1,34E+07 | 3,23E+07 | 3,23E+07 | 2,00E+07 | 2,40E+07 | 1,40E+07 | 1,40E+07 | 1,40E+07 | 1,40E+07 | 1,40E+07 | 1,40E+07 |
| E9PQQ4 | 1,54E+07 | 1,34E+07 | 3,23E+07 | 3,23E+07 | 2,00E+07 | 2,40E+07 | 1,40E+07 | 1,40E+07 | 1,40E+07 | 1,40E+07 | 1,40E+07 | 1,40E+07 |
| E9PRK8 | 2,09E+06 | 1,82E+06 | 4,39E+06 | 4,39E+06 | 2,72E+06 | 3,26E+06 | 1,90E+06 | 1,46E+06 | 2,92E+06 | 4,09E+06 | 2,63E+06 | 3,42E+06 |
| F2Z393 | 1,76E+07 | 1,53E+07 | 3,70E+07 | 3,70E+07 | 2,29E+07 | 2,75E+07 | 1,60E+07 | 1,60E+07 | 1,60E+07 | 1,60E+07 | 1,60E+07 | 1,60E+07 |
| F5GWA7 | 3,19E+06 | 2,77E+06 | 6,70E+06 | 6,70E+06 | 4,15E+06 | 4,98E+06 | 2,90E+06 | 2,90E+06 | 2,90E+06 | 2,90E+06 | 2,90E+06 | 2,90E+06 |
| F5GXH2 | 5,28E+07 | 4,59E+07 | 1,11E+08 | 1,11E+08 | 6,86E+07 | 8,24E+07 | 4,80E+07 | 4,80E+07 | 4,80E+07 | 4,80E+07 | 4,80E+07 | 4,80E+07 |
| F5GXK7 | 6,86E+07 | 5,97E+07 | 1,44E+08 | 1,44E+08 | 8,92E+07 | 1,07E+08 | 6,24E+07 | 6,24E+07 | 6,24E+07 | 6,24E+07 | 6,24E+07 | 6,24E+07 |
| F5GXZ9 | 2,64E+04 | 2,30E+04 | 5,54E+04 | 5,54E+04 | 3,43E+04 | 4,12E+04 | 2,40E+04 | 2,40E+04 | 2,40E+04 | 2,40E+04 | 1,50E+06 | 2,40E+04 |
| F5GY37 | 6,86E+07 | 5,97E+07 | 1,44E+08 | 1,44E+08 | 8,92E+07 | 1,07E+08 | 6,24E+07 | 6,24E+07 | 6,24E+07 | 6,24E+07 | 6,24E+07 | 6,24E+07 |

| | | | | | | | | | | | | |
|---|---|---|---|---|---|---|---|---|---|---|---|---|
| F5GYU3 | 6,86E+07 | 5,97E+07 | 1,44E+08 | 1,44E+08 | 8,92E+07 | 1,07E+08 | 6,24E+07 | 6,24E+07 | 6,24E+07 | 6,24E+07 | 6,24E+07 | 6,24E+07 |
| F5GZ39 | 7,99E+10 | 6,94E+10 | 1,68E+11 | 1,68E+11 | 1,04E+11 | 1,25E+11 | 7,26E+10 | 7,26E+10 | 7,26E+10 | 7,26E+10 | 7,26E+10 | 7,26E+10 |
| F5GZQ4 | 6,49E+06 | 5,64E+06 | 1,36E+07 | 1,36E+07 | 8,44E+06 | 1,01E+07 | 5,90E+06 | 5,90E+06 | 5,90E+06 | 5,90E+06 | 5,90E+06 | 5,90E+06 |
| F5H265 | 2,09E+07 | 1,82E+07 | 4,39E+07 | 4,39E+07 | 2,72E+07 | 3,26E+07 | 1,90E+07 | 1,90E+07 | 1,90E+07 | 1,90E+07 | 1,90E+07 | 1,90E+07 |
| F5H2Z3 | 6,86E+07 | 5,97E+07 | 1,44E+08 | 1,44E+08 | 8,92E+07 | 1,07E+08 | 6,24E+07 | 6,24E+07 | 6,24E+07 | 6,24E+07 | 6,24E+07 | 6,24E+07 |
| F5H388 | 2,09E+07 | 1,82E+07 | 4,39E+07 | 4,39E+07 | 2,72E+07 | 3,26E+07 | 1,90E+07 | 1,90E+07 | 1,90E+07 | 1,90E+07 | 1,90E+07 | 1,90E+07 |
| F5H3X6 | 6,86E+07 | 5,97E+07 | 1,44E+08 | 1,44E+08 | 8,92E+07 | 1,07E+08 | 6,24E+07 | 6,24E+07 | 6,24E+07 | 6,24E+07 | 6,24E+07 | 6,24E+07 |
| F5H5J4 | 6,49E+06 | 5,64E+06 | 1,36E+07 | 1,36E+07 | 8,44E+06 | 1,01E+07 | 5,90E+06 | 5,90E+06 | 5,90E+06 | 5,90E+06 | 5,90E+06 | 5,90E+06 |
| F5H6Q2 | 6,86E+07 | 5,97E+07 | 1,44E+08 | 1,44E+08 | 8,92E+07 | 1,07E+08 | 6,24E+07 | 6,24E+07 | 6,24E+07 | 6,24E+07 | 6,24E+07 | 6,24E+07 |
| F5H6W8 | 6,49E+06 | 5,64E+06 | 1,36E+07 | 1,36E+07 | 8,44E+06 | 1,01E+07 | 5,90E+06 | 5,90E+06 | 5,90E+06 | 5,90E+06 | 5,90E+06 | 5,90E+06 |
| F5H747 | 1,36E+09 | 1,19E+09 | 2,86E+09 | 2,86E+09 | 1,77E+09 | 2,13E+09 | 1,24E+09 | 1,24E+09 | 1,24E+09 | 1,24E+09 | 1,24E+09 | 1,24E+09 |
| F6QYI9 | 2,09E+06 | 1,82E+06 | 4,39E+06 | 4,39E+06 | 2,72E+06 | 3,26E+06 | 1,90E+06 | 1,90E+06 | 1,90E+06 | 1,90E+06 | 1,90E+06 | 1,90E+06 |
| F6R6M7 | 2,09E+06 | 1,82E+06 | 4,39E+06 | 4,39E+06 | 2,72E+06 | 3,26E+06 | 1,90E+06 | 1,90E+06 | 1,90E+06 | 1,90E+06 | 1,90E+06 | 1,90E+06 |
| F6S4E6 | 2,09E+06 | 1,82E+06 | 4,39E+06 | 4,39E+06 | 2,72E+06 | 3,26E+06 | 1,90E+06 | 1,90E+06 | 1,90E+06 | 1,90E+06 | 1,90E+06 | 1,90E+06 |
| F6TRA5 | 2,09E+06 | 1,82E+06 | 4,39E+06 | 4,39E+06 | 2,72E+06 | 3,26E+06 | 1,90E+06 | 1,90E+06 | 1,90E+06 | 1,90E+06 | 1,90E+06 | 1,90E+06 |
| F6WLT2 | 2,09E+06 | 1,82E+06 | 4,39E+06 | 4,39E+06 | 2,72E+06 | 3,26E+06 | 1,90E+06 | 1,90E+06 | 1,90E+06 | 1,90E+06 | 1,90E+06 | 1,90E+06 |
| F8VPE8 | 6,49E+06 | 5,64E+06 | 1,36E+07 | 1,36E+07 | 8,44E+06 | 1,01E+07 | 5,90E+06 | 4,54E+06 | 9,08E+06 | 1,27E+07 | 8,17E+06 | 1,06E+07 |
| F8VTQ5 | 4,62E+06 | 4,02E+06 | 9,70E+06 | 9,70E+06 | 6,01E+06 | 7,21E+06 | 4,20E+06 | 3,23E+06 | 6,46E+06 | 9,05E+06 | 5,82E+06 | 7,56E+06 |
| F8VU65 | 2,09E+06 | 1,82E+06 | 4,39E+06 | 4,39E+06 | 2,72E+06 | 3,26E+06 | 1,90E+06 | 1,46E+06 | 2,92E+06 | 4,09E+06 | 2,63E+06 | 3,42E+06 |
| F8VW21 | 6,49E+06 | 5,64E+06 | 1,36E+07 | 1,36E+07 | 8,44E+06 | 1,01E+07 | 5,90E+06 | 4,54E+06 | 9,08E+06 | 1,27E+07 | 8,17E+06 | 1,06E+07 |
| F8VZ49 | 4,95E+06 | 4,30E+06 | 1,04E+07 | 1,04E+07 | 6,44E+06 | 7,72E+06 | 4,50E+06 | 3,46E+06 | 6,92E+06 | 9,69E+06 | 6,23E+06 | 8,10E+06 |
| F8W079 | 2,09E+07 | 1,82E+07 | 4,39E+07 | 4,39E+07 | 2,72E+07 | 3,26E+07 | 1,90E+07 | 1,90E+07 | 1,90E+07 | 1,90E+07 | 1,90E+07 | 1,90E+07 |
| F8W0P7 | 2,97E+06 | 2,58E+06 | 6,24E+06 | 6,24E+06 | 3,86E+06 | 4,63E+06 | 2,70E+06 | 2,70E+06 | 2,70E+06 | 2,70E+06 | 2,70E+06 | 2,70E+06 |
| F8W646 | 4,62E+06 | 4,02E+06 | 9,70E+06 | 9,70E+06 | 6,01E+06 | 7,21E+06 | 4,20E+06 | 3,23E+06 | 6,46E+06 | 9,05E+06 | 5,82E+06 | 7,56E+06 |
| F8W6I7 | 4,62E+06 | 4,02E+06 | 9,70E+06 | 9,70E+06 | 6,01E+06 | 7,21E+06 | 4,20E+06 | 3,23E+06 | 6,46E+06 | 9,05E+06 | 5,82E+06 | 7,56E+06 |
| F8WCF6 | 2,86E+07 | 2,49E+07 | 6,01E+07 | 6,01E+07 | 3,72E+07 | 4,46E+07 | 2,60E+07 | 2,00E+07 | 4,00E+07 | 5,60E+07 | 3,60E+07 | 4,68E+07 |
| F8WD59 | 4,62E+06 | 4,02E+06 | 9,70E+06 | 9,70E+06 | 6,01E+06 | 7,21E+06 | 4,20E+06 | 3,23E+06 | 6,46E+06 | 9,05E+06 | 5,82E+06 | 7,56E+06 |
| F8WD96 | 2,86E+06 | 2,49E+06 | 6,01E+06 | 6,01E+06 | 3,72E+06 | 4,46E+06 | 2,60E+06 | 2,60E+06 | 6,00E+06 | 6,00E+06 | 1,60E+06 | 1,60E+06 |
| F8WDD7 | 1,39E+10 | 1,21E+10 | 2,91E+10 | 2,91E+10 | 1,80E+10 | 2,16E+10 | 1,26E+10 | 9,69E+09 | 1,94E+10 | 2,71E+10 | 1,74E+10 | 2,27E+10 |

| | | | | | | | | | | | | |
|---|---|---|---|---|---|---|---|---|---|---|---|---|
| F8WE65 | 9,90E+07 | 8,61E+07 | 2,08E+08 | 2,08E+08 | 1,29E+08 | 1,54E+08 | 9,00E+07 | 9,00E+07 | 9,00E+07 | 9,00E+07 | 9,00E+07 | 9,00E+07 |
| G3V192 | 2,09E+06 | 1,82E+06 | 4,39E+06 | 4,39E+06 | 2,72E+06 | 3,26E+06 | 1,90E+06 | 1,46E+06 | 2,92E+06 | 4,09E+06 | 2,63E+06 | 3,42E+06 |
| G3V1A4 | 3,96E+07 | 3,44E+07 | 8,32E+07 | 8,32E+07 | 5,15E+07 | 6,18E+07 | 3,60E+07 | 3,60E+07 | 3,60E+07 | 3,60E+07 | 3,60E+07 | 3,60E+07 |
| G3V1D1 | 2,09E+06 | 1,82E+06 | 4,39E+06 | 4,39E+06 | 2,72E+06 | 3,26E+06 | 1,90E+06 | 1,46E+06 | 2,92E+06 | 4,09E+06 | 2,63E+06 | 3,42E+06 |
| G3V251 | 3,85E+07 | 3,35E+07 | 8,09E+07 | 8,09E+07 | 5,01E+07 | 6,01E+07 | 3,50E+07 | 3,50E+07 | 3,50E+07 | 3,50E+07 | 3,50E+07 | 3,50E+07 |
| G3V2H6 | 1,98E+07 | 1,72E+07 | 4,16E+07 | 4,16E+07 | 2,57E+07 | 3,09E+07 | 1,80E+07 | 1,80E+07 | 1,80E+07 | 1,80E+07 | 1,80E+07 | 1,80E+07 |
| G3V2Q1 | 3,85E+07 | 3,35E+07 | 8,09E+07 | 8,09E+07 | 5,01E+07 | 6,01E+07 | 3,50E+07 | 3,50E+07 | 3,50E+07 | 3,50E+07 | 3,50E+07 | 3,50E+07 |
| G3V3K6 | 3,85E+07 | 3,35E+07 | 8,09E+07 | 8,09E+07 | 5,01E+07 | 6,01E+07 | 3,50E+07 | 3,50E+07 | 3,50E+07 | 3,50E+07 | 3,50E+07 | 3,50E+07 |
| G3V4C1 | 3,85E+07 | 3,35E+07 | 8,09E+07 | 8,09E+07 | 5,01E+07 | 6,01E+07 | 3,50E+07 | 3,50E+07 | 3,50E+07 | 3,50E+07 | 3,50E+07 | 3,50E+07 |
| G3V4M8 | 2,75E+07 | 2,39E+07 | 5,78E+07 | 5,78E+07 | 3,58E+07 | 4,29E+07 | 2,50E+07 | 2,50E+07 | 2,50E+07 | 2,50E+07 | 2,50E+07 | 2,50E+07 |
| G3V4W0 | 3,85E+07 | 3,35E+07 | 8,09E+07 | 8,09E+07 | 5,01E+07 | 6,01E+07 | 3,50E+07 | 3,50E+07 | 3,50E+07 | 3,50E+07 | 3,50E+07 | 3,50E+07 |
| G3V555 | 3,85E+07 | 3,35E+07 | 8,09E+07 | 8,09E+07 | 5,01E+07 | 6,01E+07 | 3,50E+07 | 3,50E+07 | 3,50E+07 | 3,50E+07 | 3,50E+07 | 3,50E+07 |
| G3V575 | 3,85E+07 | 3,35E+07 | 8,09E+07 | 8,09E+07 | 5,01E+07 | 6,01E+07 | 3,50E+07 | 3,50E+07 | 3,50E+07 | 3,50E+07 | 3,50E+07 | 3,50E+07 |
| G3V576 | 3,85E+07 | 3,35E+07 | 8,09E+07 | 8,09E+07 | 5,01E+07 | 6,01E+07 | 3,50E+07 | 3,50E+07 | 3,50E+07 | 3,50E+07 | 3,50E+07 | 3,50E+07 |
| G3V5X6 | 3,85E+07 | 3,35E+07 | 8,09E+07 | 8,09E+07 | 5,01E+07 | 6,01E+07 | 3,50E+07 | 3,50E+07 | 3,50E+07 | 3,50E+07 | 3,50E+07 | 3,50E+07 |
| G3XAL0 | 5,28E+05 | 4,59E+05 | 1,11E+06 | 1,11E+06 | 6,86E+05 | 8,24E+05 | 4,80E+05 | 4,80E+05 | 4,80E+05 | 2,90E+07 | 5,10E+06 | 1,20E+07 |
| G5E9P6 | 2,64E+04 | 2,30E+04 | 5,54E+04 | 5,54E+04 | 3,43E+04 | 4,12E+04 | 2,40E+04 | 2,40E+04 | 2,40E+04 | 2,40E+04 | 1,50E+06 | 2,40E+04 |
| G9K388 | 6,86E+07 | 5,97E+07 | 1,44E+08 | 1,44E+08 | 8,92E+07 | 1,07E+08 | 6,24E+07 | 6,24E+07 | 6,24E+07 | 6,24E+07 | 6,24E+07 | 6,24E+07 |
| G9K389 | 5,50E+06 | 4,78E+06 | 1,16E+07 | 1,16E+07 | 7,15E+06 | 8,58E+06 | 5,00E+06 | 5,00E+06 | 5,00E+06 | 1,24E+08 | 1,24E+08 | 1,24E+08 |
| H0Y3Z3 | 1,65E+06 | 1,43E+06 | 3,47E+06 | 3,47E+06 | 2,15E+06 | 2,57E+06 | 1,50E+06 | 1,50E+06 | 1,50E+06 | 1,50E+06 | 1,50E+06 | 1,50E+06 |
| H0YB80 | 4,62E+06 | 4,02E+06 | 9,70E+06 | 9,70E+06 | 6,01E+06 | 7,21E+06 | 4,20E+06 | 3,23E+06 | 6,46E+06 | 9,05E+06 | 5,82E+06 | 7,56E+06 |
| H0YFC6 | 2,20E+07 | 1,91E+07 | 4,62E+07 | 4,62E+07 | 2,86E+07 | 3,43E+07 | 2,00E+07 | 2,00E+07 | 2,00E+07 | 2,00E+07 | 2,00E+07 | 2,00E+07 |
| H0YH81 | 1,43E+07 | 1,24E+07 | 3,00E+07 | 3,00E+07 | 1,86E+07 | 2,23E+07 | 1,30E+07 | 1,30E+07 | 1,30E+07 | 1,30E+07 | 1,30E+07 | 1,30E+07 |
| H0YKV8 | 3,19E+07 | 2,77E+07 | 6,70E+07 | 6,70E+07 | 4,15E+07 | 4,98E+07 | 2,90E+07 | 2,90E+07 | 2,90E+07 | 2,90E+07 | 2,90E+07 | 2,90E+07 |
| H0YMD9 | 3,19E+07 | 2,77E+07 | 6,70E+07 | 6,70E+07 | 4,15E+07 | 4,98E+07 | 2,90E+07 | 2,90E+07 | 2,90E+07 | 2,90E+07 | 2,90E+07 | 2,90E+07 |
| H0YN52 | 6,86E+07 | 5,97E+07 | 1,44E+08 | 1,44E+08 | 8,92E+07 | 1,07E+08 | 6,24E+07 | 6,24E+07 | 6,24E+07 | 6,24E+07 | 6,24E+07 | 6,24E+07 |
| H0YNA0 | 6,86E+07 | 5,97E+07 | 1,44E+08 | 1,44E+08 | 8,92E+07 | 1,07E+08 | 6,24E+07 | 6,24E+07 | 6,24E+07 | 6,24E+07 | 6,24E+07 | 6,24E+07 |
| H3BMQ8 | 2,09E+07 | 1,82E+07 | 4,39E+07 | 4,39E+07 | 2,72E+07 | 3,26E+07 | 1,90E+07 | 1,90E+07 | 1,90E+07 | 1,90E+07 | 1,90E+07 | 1,90E+07 |
| H3BPS8 | 3,63E+07 | 3,16E+07 | 7,62E+07 | 7,62E+07 | 4,72E+07 | 5,66E+07 | 3,30E+07 | 3,30E+07 | 3,30E+07 | 3,30E+07 | 3,30E+07 | 3,30E+07 |

| | | | | | | | | | | | | |
|---|---|---|---|---|---|---|---|---|---|---|---|---|
| H3BQ34 | 2,42E+07 | 2,10E+07 | 5,08E+07 | 5,08E+07 | 3,15E+07 | 3,78E+07 | 2,20E+07 | 2,20E+07 | 2,20E+07 | 2,20E+07 | 2,20E+07 | 2,20E+07 |
| H3BR04 | 3,19E+07 | 2,77E+07 | 6,70E+07 | 6,70E+07 | 4,15E+07 | 4,98E+07 | 2,90E+07 | 2,90E+07 | 2,90E+07 | 2,90E+07 | 2,90E+07 | 2,90E+07 |
| H3BR70 | 2,46E+07 | 2,14E+07 | 5,17E+07 | 5,17E+07 | 3,20E+07 | 3,84E+07 | 2,24E+07 | 2,24E+07 | 2,24E+07 | 2,80E+07 | 2,80E+07 | 2,80E+07 |
| H3BTN5 | 3,56E+08 | 3,10E+08 | 7,48E+08 | 7,48E+08 | 4,63E+08 | 5,56E+08 | 3,24E+08 | 3,24E+08 | 3,24E+08 | 3,24E+08 | 3,24E+08 | 3,24E+08 |
| H3BUH7 | 3,56E+08 | 3,10E+08 | 7,48E+08 | 7,48E+08 | 4,63E+08 | 5,56E+08 | 3,24E+08 | 3,24E+08 | 3,24E+08 | 3,24E+08 | 3,24E+08 | 3,24E+08 |
| H7BY16 | 1,76E+07 | 1,53E+07 | 3,70E+07 | 3,70E+07 | 2,29E+07 | 2,75E+07 | 1,60E+07 | 1,60E+07 | 1,60E+07 | 1,60E+07 | 1,60E+07 | 1,60E+07 |
| H7BZ94 | 1,65E+06 | 1,43E+06 | 3,47E+06 | 3,47E+06 | 2,15E+06 | 2,57E+06 | 1,50E+06 | 1,50E+06 | 1,50E+06 | 1,50E+06 | 1,50E+06 | 1,50E+06 |
| H7C1V0 | 1,43E+07 | 1,24E+07 | 3,00E+07 | 3,00E+07 | 1,86E+07 | 2,23E+07 | 1,30E+07 | 1,30E+07 | 1,30E+07 | 1,30E+07 | 1,30E+07 | 1,30E+07 |
| H7C3Z9 | 6,49E+06 | 5,64E+06 | 1,36E+07 | 1,36E+07 | 8,44E+06 | 1,01E+07 | 5,90E+06 | 4,54E+06 | 9,08E+06 | 1,27E+07 | 8,17E+06 | 1,06E+07 |
| H7C4K3 | 3,00E+07 | 6,00E+07 | 1,50E+07 | 4,50E+07 | 2,00E+07 | 6,00E+07 | 2,90E+07 | 2,90E+07 | 2,90E+07 | 2,90E+07 | 2,90E+07 | 2,90E+07 |
| I3L1H6 | 2,09E+06 | 1,82E+06 | 4,39E+06 | 4,39E+06 | 2,72E+06 | 3,26E+06 | 1,90E+06 | 1,46E+06 | 2,92E+06 | 4,09E+06 | 2,63E+06 | 3,42E+06 |
| I3L246 | 8,58E+06 | 7,46E+06 | 1,80E+07 | 1,80E+07 | 1,12E+07 | 1,34E+07 | 7,80E+06 | 7,80E+06 | 7,80E+06 | 7,80E+06 | 7,80E+06 | 7,80E+06 |
| I3L312 | 2,20E+06 | 1,91E+06 | 4,62E+06 | 4,62E+06 | 2,86E+06 | 3,43E+06 | 2,00E+06 | 2,00E+06 | 2,00E+06 | 2,40E+04 | 2,40E+04 | 2,40E+04 |
| I3L397 | 2,86E+06 | 2,49E+06 | 6,01E+06 | 6,01E+06 | 3,72E+06 | 4,46E+06 | 2,60E+06 | 2,60E+06 | 2,60E+06 | 5,90E+06 | 4,40E+06 | 3,40E+06 |
| I3L398 | 3,19E+07 | 2,77E+07 | 6,70E+07 | 6,70E+07 | 4,15E+07 | 4,98E+07 | 2,90E+07 | 2,90E+07 | 2,90E+07 | 2,90E+07 | 2,90E+07 | 2,90E+07 |
| I3L3I9 | 2,09E+06 | 1,82E+06 | 4,39E+06 | 4,39E+06 | 2,72E+06 | 3,26E+06 | 1,90E+06 | 1,46E+06 | 2,92E+06 | 4,09E+06 | 2,63E+06 | 3,42E+06 |
| I3L3P7 | 7,37E+06 | 6,41E+06 | 1,55E+07 | 1,55E+07 | 9,58E+06 | 1,15E+07 | 6,70E+06 | 6,70E+06 | 6,70E+06 | 6,70E+06 | 6,70E+06 | 6,70E+06 |
| I3L3W9 | 2,09E+06 | 1,82E+06 | 4,39E+06 | 4,39E+06 | 2,72E+06 | 3,26E+06 | 1,90E+06 | 1,46E+06 | 2,92E+06 | 4,09E+06 | 2,63E+06 | 3,42E+06 |
| I3L4E5 | 6,49E+06 | 5,64E+06 | 1,36E+07 | 1,36E+07 | 8,44E+06 | 1,01E+07 | 5,90E+06 | 4,54E+06 | 9,08E+06 | 1,27E+07 | 8,17E+06 | 1,06E+07 |
| I3L4M2 | 1,65E+06 | 1,43E+06 | 3,47E+06 | 3,47E+06 | 2,15E+06 | 2,57E+06 | 1,50E+06 | 1,50E+06 | 1,50E+06 | 1,50E+06 | 1,50E+06 | 1,50E+06 |
| I3L504 | 2,86E+06 | 2,49E+06 | 6,01E+06 | 6,01E+06 | 3,72E+06 | 4,46E+06 | 2,60E+06 | 2,60E+06 | 2,60E+06 | 5,90E+06 | 4,40E+06 | 3,40E+06 |
| I3NI03 | 1,32E+06 | 1,15E+06 | 2,77E+06 | 2,77E+06 | 1,72E+06 | 2,06E+06 | 1,20E+06 | 1,20E+06 | 1,20E+06 | 1,20E+06 | 1,20E+06 | 1,20E+06 |
| J3KMX3 | 3,63E+06 | 3,16E+06 | 7,62E+06 | 7,62E+06 | 4,72E+06 | 5,66E+06 | 3,30E+06 | 2,54E+06 | 5,08E+06 | 7,11E+06 | 4,57E+06 | 5,94E+06 |
| J3KPD9 | 4,40E+07 | 3,83E+07 | 9,24E+07 | 9,24E+07 | 5,72E+07 | 6,86E+07 | 4,00E+07 | 4,00E+07 | 4,00E+07 | 4,00E+07 | 4,00E+07 | 4,00E+07 |
| J3KPX7 | 6,86E+07 | 5,97E+07 | 1,44E+08 | 1,44E+08 | 8,92E+07 | 1,07E+08 | 6,24E+07 | 6,24E+07 | 6,24E+07 | 6,24E+07 | 6,24E+07 | 6,24E+07 |
| J3KQE5 | 2,42E+07 | 2,10E+07 | 5,08E+07 | 5,08E+07 | 3,15E+07 | 3,78E+07 | 2,20E+07 | 2,30E+06 | 2,00E+06 | 2,20E+07 | 2,30E+06 | 2,00E+06 |
| J3KS25 | 2,64E+04 | 2,30E+04 | 5,54E+04 | 5,54E+04 | 3,43E+04 | 4,12E+04 | 2,40E+04 | 2,40E+04 | 2,40E+04 | 2,40E+07 | 2,40E+04 | 2,40E+04 |
| J3KSV6 | 2,64E+07 | 2,30E+07 | 5,54E+07 | 5,54E+07 | 3,43E+07 | 4,12E+07 | 2,40E+07 | 2,40E+07 | 2,40E+07 | 2,40E+07 | 2,40E+07 | 2,40E+07 |
| J3KSZ0 | 2,64E+04 | 2,30E+04 | 5,54E+04 | 5,54E+04 | 3,43E+04 | 4,12E+04 | 2,40E+04 | 2,40E+04 | 2,40E+04 | 2,40E+07 | 2,40E+04 | 2,40E+04 |

| | | | | | | | | | | | | |
|---|---|---|---|---|---|---|---|---|---|---|---|---|
| J3KT12 | 2,64E+04 | 2,30E+04 | 5,54E+04 | 5,54E+04 | 3,43E+04 | 4,12E+04 | 2,40E+04 | 2,40E+04 | 2,40E+04 | 2,40E+07 | 2,40E+04 | 2,40E+04 |
| J3KTB5 | 2,64E+04 | 2,30E+04 | 5,54E+04 | 5,54E+04 | 3,43E+04 | 4,12E+04 | 2,40E+04 | 2,40E+04 | 2,40E+04 | 2,40E+07 | 2,40E+04 | 2,40E+04 |
| J3KTN0 | 2,64E+04 | 2,30E+04 | 5,54E+04 | 5,54E+04 | 3,43E+04 | 4,12E+04 | 2,40E+04 | 2,40E+04 | 2,40E+04 | 2,40E+07 | 2,40E+04 | 2,40E+04 |
| J3QKN0 | 6,86E+07 | 5,97E+07 | 1,44E+08 | 1,44E+08 | 8,92E+07 | 1,07E+08 | 6,24E+07 | 6,24E+07 | 6,24E+07 | 6,24E+07 | 6,24E+07 | 6,24E+07 |
| J3QKP5 | 2,64E+07 | 2,30E+07 | 5,54E+07 | 5,54E+07 | 3,43E+07 | 4,12E+07 | 2,40E+07 | 2,40E+07 | 2,40E+07 | 2,40E+07 | 2,40E+07 | 2,40E+07 |
| J3QL43 | 2,64E+04 | 2,30E+04 | 5,54E+04 | 5,54E+04 | 3,43E+04 | 4,12E+04 | 2,40E+04 | 2,40E+04 | 2,40E+04 | 2,40E+07 | 2,40E+04 | 2,40E+04 |
| J3QLI9 | 2,42E+06 | 2,10E+06 | 5,08E+06 | 5,08E+06 | 3,15E+06 | 3,78E+06 | 2,20E+06 | 2,20E+06 | 2,20E+06 | 2,60E+06 | 2,60E+10 | 6,26E+09 |
| J3QLN6 | 2,64E+04 | 2,30E+04 | 5,54E+04 | 5,54E+04 | 3,43E+04 | 4,12E+04 | 2,40E+04 | 2,40E+04 | 2,40E+04 | 2,40E+07 | 2,40E+04 | 2,40E+04 |
| J3QR64 | 2,64E+04 | 2,30E+04 | 5,54E+04 | 5,54E+04 | 3,43E+04 | 4,12E+04 | 2,40E+04 | 2,40E+04 | 2,40E+04 | 2,40E+07 | 2,40E+04 | 2,40E+04 |
| J3QS39 | 6,86E+07 | 5,97E+07 | 1,44E+08 | 1,44E+08 | 8,92E+07 | 1,07E+08 | 6,24E+07 | 6,24E+07 | 6,24E+07 | 6,24E+07 | 6,24E+07 | 6,24E+07 |
| J3QS69 | 2,64E+04 | 2,30E+04 | 5,54E+04 | 5,54E+04 | 3,43E+04 | 4,12E+04 | 2,40E+04 | 2,40E+04 | 2,40E+04 | 2,40E+07 | 2,40E+04 | 2,40E+04 |
| J3QSA3 | 7,99E+10 | 6,94E+10 | 1,68E+11 | 1,68E+11 | 1,04E+11 | 1,25E+11 | 7,26E+10 | 7,26E+10 | 7,26E+10 | 7,26E+10 | 7,26E+10 | 7,26E+10 |
| J3QTR3 | 1,36E+09 | 1,19E+09 | 2,86E+09 | 2,86E+09 | 1,77E+09 | 2,13E+09 | 1,24E+09 | 1,24E+09 | 1,24E+09 | 1,24E+09 | 1,24E+09 | 1,24E+09 |
| K7EIL4 | 1,39E+10 | 1,21E+10 | 2,91E+10 | 2,91E+10 | 1,80E+10 | 2,16E+10 | 1,26E+10 | 9,69E+09 | 1,94E+10 | 2,71E+10 | 1,74E+10 | 2,27E+10 |
| K7EIT4 | 2,86E+07 | 2,49E+07 | 6,01E+07 | 6,01E+07 | 3,72E+07 | 4,46E+07 | 2,60E+07 | 2,00E+07 | 4,00E+07 | 5,60E+07 | 3,60E+07 | 4,68E+07 |
| K7EJ44 | 1,65E+07 | 1,43E+07 | 3,47E+07 | 3,47E+07 | 2,15E+07 | 2,57E+07 | 1,50E+07 | 1,50E+07 | 1,50E+07 | 1,50E+07 | 1,50E+07 | 1,50E+07 |
| K7EK07 | 2,09E+07 | 1,82E+07 | 4,39E+07 | 4,39E+07 | 2,72E+07 | 3,26E+07 | 1,90E+07 | 1,90E+07 | 1,90E+07 | 1,90E+07 | 1,90E+07 | 1,90E+07 |
| K7EK77 | 6,16E+06 | 5,36E+06 | 1,29E+07 | 1,29E+07 | 8,01E+06 | 9,61E+06 | 5,60E+06 | 5,60E+06 | 5,60E+06 | 5,60E+06 | 5,60E+06 | 5,60E+06 |
| K7EKH5 | 2,64E+07 | 2,30E+07 | 5,54E+07 | 5,54E+07 | 3,43E+07 | 4,12E+07 | 2,40E+07 | 2,40E+07 | 2,40E+07 | 2,40E+07 | 2,40E+07 | 2,40E+07 |
| K7ELN9 | 2,09E+06 | 1,82E+06 | 4,39E+06 | 4,39E+06 | 2,72E+06 | 3,26E+06 | 1,90E+06 | 1,46E+06 | 2,92E+06 | 4,09E+06 | 2,63E+06 | 3,42E+06 |
| K7ELR7 | 3,63E+06 | 3,16E+06 | 7,62E+06 | 7,62E+06 | 4,72E+06 | 5,66E+06 | 3,30E+06 | 2,54E+06 | 5,08E+06 | 7,11E+06 | 4,57E+06 | 5,94E+06 |
| K7EM20 | 1,39E+10 | 1,21E+10 | 2,91E+10 | 2,91E+10 | 1,80E+10 | 2,16E+10 | 1,26E+10 | 9,69E+09 | 1,94E+10 | 2,71E+10 | 1,74E+10 | 2,27E+10 |
| K7EM49 | 2,09E+06 | 1,82E+06 | 4,39E+06 | 4,39E+06 | 2,72E+06 | 3,26E+06 | 1,90E+06 | 1,46E+06 | 2,92E+06 | 4,09E+06 | 2,63E+06 | 3,42E+06 |
| K7EM90 | 2,09E+07 | 1,82E+07 | 4,39E+07 | 4,39E+07 | 2,72E+07 | 3,26E+07 | 1,90E+07 | 1,90E+07 | 1,90E+07 | 1,90E+07 | 1,90E+07 | 1,90E+07 |
| K7EMN2 | 2,09E+06 | 1,82E+06 | 4,39E+06 | 4,39E+06 | 2,72E+06 | 3,26E+06 | 1,90E+06 | 1,46E+06 | 2,92E+06 | 4,09E+06 | 2,63E+06 | 3,42E+06 |
| K7EMV3 | 6,86E+07 | 5,97E+07 | 1,44E+08 | 1,44E+08 | 8,92E+07 | 1,07E+08 | 6,24E+07 | 6,24E+07 | 6,24E+07 | 6,24E+07 | 6,24E+07 | 6,24E+07 |
| K7EN69 | 2,09E+06 | 1,82E+06 | 4,39E+06 | 4,39E+06 | 2,72E+06 | 3,26E+06 | 1,90E+06 | 1,90E+06 | 1,90E+06 | 1,90E+06 | 1,90E+06 | 1,90E+06 |
| K7ENA0 | 1,39E+10 | 1,21E+10 | 2,91E+10 | 2,91E+10 | 1,80E+10 | 2,16E+10 | 1,26E+10 | 9,69E+09 | 1,94E+10 | 2,71E+10 | 1,74E+10 | 2,27E+10 |
| K7ENJ4 | 6,16E+06 | 5,36E+06 | 1,29E+07 | 1,29E+07 | 8,01E+06 | 9,61E+06 | 5,60E+06 | 5,60E+06 | 5,60E+06 | 5,60E+06 | 5,60E+06 | 5,60E+06 |

| | | | | | | | | | | | | |
|---|---|---|---|---|---|---|---|---|---|---|---|---|
| K7EP41 | 2,09E+06 | 1,82E+06 | 4,39E+06 | 4,39E+06 | 2,72E+06 | 3,26E+06 | 1,90E+06 | 1,46E+06 | 2,92E+06 | 4,09E+06 | 2,63E+06 | 3,42E+06 |
| K7EPF6 | 2,09E+06 | 1,82E+06 | 4,39E+06 | 4,39E+06 | 2,72E+06 | 3,26E+06 | 1,90E+06 | 1,46E+06 | 2,92E+06 | 4,09E+06 | 2,63E+06 | 3,42E+06 |
| K7EPJ3 | 2,09E+06 | 1,82E+06 | 4,39E+06 | 4,39E+06 | 2,72E+06 | 3,26E+06 | 1,90E+06 | 1,90E+06 | 1,90E+06 | 1,90E+06 | 1,90E+06 | 1,90E+06 |
| K7EPY4 | 1,39E+10 | 1,21E+10 | 2,91E+10 | 2,91E+10 | 1,80E+10 | 2,16E+10 | 1,26E+10 | 9,69E+09 | 1,94E+10 | 2,71E+10 | 1,74E+10 | 2,27E+10 |
| K7EQ48 | 2,09E+06 | 1,82E+06 | 4,39E+06 | 4,39E+06 | 2,72E+06 | 3,26E+06 | 1,90E+06 | 1,46E+06 | 2,92E+06 | 4,09E+06 | 2,63E+06 | 3,42E+06 |
| K7EQN7 | 2,09E+06 | 1,82E+06 | 4,39E+06 | 4,39E+06 | 2,72E+06 | 3,26E+06 | 1,90E+06 | 1,90E+06 | 1,90E+06 | 1,90E+06 | 1,90E+06 | 1,90E+06 |
| K7ERC6 | 2,86E+07 | 2,49E+07 | 6,01E+07 | 6,01E+07 | 3,72E+07 | 4,46E+07 | 2,60E+07 | 2,00E+07 | 4,00E+07 | 5,60E+07 | 3,60E+07 | 4,68E+07 |
| K7ERK8 | 3,63E+06 | 3,16E+06 | 7,62E+06 | 7,62E+06 | 4,72E+06 | 5,66E+06 | 3,30E+06 | 2,54E+06 | 5,08E+06 | 7,11E+06 | 4,57E+06 | 5,94E+06 |
| K7ERX7 | 1,39E+10 | 1,21E+10 | 2,91E+10 | 2,91E+10 | 1,80E+10 | 2,16E+10 | 1,26E+10 | 9,69E+09 | 1,94E+10 | 2,71E+10 | 1,74E+10 | 2,27E+10 |
| K7ES00 | 2,09E+07 | 1,82E+07 | 4,39E+07 | 4,39E+07 | 2,72E+07 | 3,26E+07 | 1,90E+07 | 1,90E+07 | 1,90E+07 | 1,90E+07 | 1,90E+07 | 1,90E+07 |
| K9MS24 | 4,62E+06 | 4,02E+06 | 9,70E+06 | 9,70E+06 | 6,01E+06 | 7,21E+06 | 4,20E+06 | 3,23E+06 | 6,46E+06 | 9,05E+06 | 5,82E+06 | 7,56E+06 |
| L0R849 | 2,09E+06 | 1,82E+06 | 4,39E+06 | 4,39E+06 | 2,72E+06 | 3,26E+06 | 1,90E+06 | 1,46E+06 | 2,92E+06 | 4,09E+06 | 2,63E+06 | 3,42E+06 |
| L8B196 | 1,36E+09 | 1,19E+09 | 2,86E+09 | 2,86E+09 | 1,77E+09 | 2,13E+09 | 1,24E+09 | 1,24E+09 | 1,24E+09 | 1,24E+09 | 1,24E+09 | 1,24E+09 |
| L8B4I8 | 1,36E+09 | 1,19E+09 | 2,86E+09 | 2,86E+09 | 1,77E+09 | 2,13E+09 | 1,24E+09 | 1,24E+09 | 1,24E+09 | 1,24E+09 | 1,24E+09 | 1,24E+09 |
| L8B4J3 | 1,36E+09 | 1,19E+09 | 2,86E+09 | 2,86E+09 | 1,77E+09 | 2,13E+09 | 1,24E+09 | 1,24E+09 | 1,24E+09 | 1,24E+09 | 1,24E+09 | 1,24E+09 |
| L8B4M0 | 1,36E+09 | 1,19E+09 | 2,86E+09 | 2,86E+09 | 1,77E+09 | 2,13E+09 | 1,24E+09 | 1,24E+09 | 1,24E+09 | 1,24E+09 | 1,24E+09 | 1,24E+09 |
| L8B4R0 | 1,36E+09 | 1,19E+09 | 2,86E+09 | 2,86E+09 | 1,77E+09 | 2,13E+09 | 1,24E+09 | 1,24E+09 | 1,24E+09 | 1,24E+09 | 1,24E+09 | 1,24E+09 |
| L8B4Z6 | 1,36E+09 | 1,19E+09 | 2,86E+09 | 2,86E+09 | 1,77E+09 | 2,13E+09 | 1,24E+09 | 1,24E+09 | 1,24E+09 | 1,24E+09 | 1,24E+09 | 1,24E+09 |
| M0QX76 | 2,64E+04 | 2,30E+04 | 5,54E+04 | 5,54E+04 | 3,43E+04 | 4,12E+04 | 2,40E+04 | 2,40E+04 | 2,40E+04 | 2,40E+04 | 2,40E+04 | 2,40E+04 |
| M0R1M5 | 2,64E+04 | 2,30E+04 | 5,54E+04 | 5,54E+04 | 3,43E+04 | 4,12E+04 | 2,40E+04 | 2,40E+04 | 2,40E+04 | 2,40E+04 | 2,40E+04 | 2,40E+04 |
| M0R1M6 | 7,59E+07 | 6,60E+07 | 1,59E+08 | 1,59E+08 | 9,87E+07 | 1,18E+08 | 6,90E+07 | 6,90E+07 | 6,90E+07 | 6,90E+07 | 6,90E+07 | 6,90E+07 |
| M0R210 | 6,60E+06 | 5,74E+06 | 1,39E+07 | 1,39E+07 | 8,58E+06 | 1,03E+07 | 6,00E+06 | 6,00E+06 | 6,00E+06 | 6,00E+06 | 6,00E+06 | 6,00E+06 |
| M0R2S1 | 7,59E+07 | 6,60E+07 | 1,59E+08 | 1,59E+08 | 9,87E+07 | 1,18E+08 | 6,90E+07 | 6,90E+07 | 6,90E+07 | 6,90E+07 | 6,90E+07 | 6,90E+07 |
| M0R3H0 | 2,64E+04 | 2,30E+04 | 5,54E+04 | 5,54E+04 | 3,43E+04 | 4,12E+04 | 2,40E+04 | 2,40E+04 | 2,40E+04 | 2,40E+04 | 2,40E+04 | 2,40E+04 |
| O00148 | 3,63E+06 | 3,16E+06 | 7,62E+06 | 7,62E+06 | 4,72E+06 | 5,66E+06 | 3,30E+06 | 3,30E+06 | 3,30E+06 | 3,30E+06 | 3,30E+06 | 3,30E+06 |
| O14495 | 1,00E+07 | 5,00E+06 | 3,80E+06 | 1,90E+06 | 5,00E+07 | 1,67E+07 | 5,00E+07 | 5,00E+07 | 5,00E+07 | 5,00E+07 | 5,00E+07 | 5,00E+07 |
| O14920 | 3,00E+07 | 3,00E+07 | 3,80E+06 | 1,90E+06 | 1,70E+07 | 5,67E+06 | 2,90E+07 | 2,90E+07 | 3,00E+07 | 1,50E+07 | 3,80E+06 | 3,80E+06 |
| O15111 | 1,20E+07 | 1,20E+07 | 3,80E+06 | 1,90E+06 | 2,40E+07 | 8,00E+06 | 2,90E+07 | 2,90E+07 | 1,20E+07 | 6,00E+06 | 3,80E+06 | 3,80E+06 |
| O43776 | 2,09E+06 | 1,82E+06 | 4,39E+06 | 4,39E+06 | 2,72E+06 | 3,26E+06 | 1,90E+06 | 1,46E+06 | 2,92E+06 | 4,09E+06 | 2,63E+06 | 3,42E+06 |

| | | | | | | | | | | | | |
|---|---|---|---|---|---|---|---|---|---|---|---|---|
| O60218 | 2,09E+06 | 1,82E+06 | 4,39E+06 | 4,39E+06 | 2,72E+06 | 3,26E+06 | 1,90E+06 | 1,46E+06 | 2,92E+06 | 4,09E+06 | 2,63E+06 | 3,42E+06 |
| O60361 | 2,64E+04 | 2,30E+04 | 5,54E+04 | 5,54E+04 | 3,43E+04 | 4,12E+04 | 2,40E+04 | 2,40E+04 | 2,40E+04 | 2,00E+07 | 2,24E+07 | 3,80E+06 |
| O60812 | 1,98E+07 | 1,72E+07 | 4,16E+07 | 4,16E+07 | 2,57E+07 | 3,09E+07 | 1,80E+07 | 1,80E+07 | 1,80E+07 | 1,80E+07 | 1,80E+07 | 1,80E+07 |
| O61565 | 2,30E+07 | 2,30E+07 | 3,80E+06 | 1,90E+06 | 2,50E+07 | 8,33E+06 | 3,80E+06 | 3,80E+06 | 2,25E+07 | 2,25E+07 | 3,45E+07 | 1,73E+07 |
| O75322 | 1,98E+07 | 1,72E+07 | 4,16E+07 | 4,16E+07 | 2,57E+07 | 3,09E+07 | 1,80E+07 | 1,00E+03 | 1,90E+06 | 1,80E+07 | 1,00E+03 | 1,90E+06 |
| O88619 | 2,90E+07 | 2,90E+07 | 3,80E+06 | 1,90E+06 | 3,60E+07 | 1,20E+07 | 2,90E+07 | 2,90E+07 | 2,90E+07 | 1,45E+07 | 4,50E+07 | 2,25E+07 |
| O95994 | 6,49E+06 | 5,64E+06 | 1,36E+07 | 1,36E+07 | 8,44E+06 | 1,01E+07 | 5,90E+06 | 4,54E+06 | 9,08E+06 | 1,27E+07 | 8,17E+06 | 1,06E+07 |
| P00352 | 6,49E+06 | 5,64E+06 | 1,36E+07 | 1,36E+07 | 8,44E+06 | 1,01E+07 | 5,90E+06 | 4,54E+06 | 9,08E+06 | 1,27E+07 | 8,17E+06 | 1,06E+07 |
| P00505 | 1,39E+10 | 1,21E+10 | 2,91E+10 | 2,91E+10 | 1,80E+10 | 2,16E+10 | 1,26E+10 | 9,69E+09 | 1,94E+10 | 2,71E+10 | 1,74E+10 | 2,27E+10 |
| P01023 | 1,65E+06 | 1,43E+06 | 3,47E+06 | 3,47E+06 | 2,15E+06 | 2,57E+06 | 1,50E+06 | 1,50E+06 | 1,50E+06 | 1,50E+06 | 1,50E+06 | 1,50E+06 |
| P02545 | 2,64E+04 | 2,30E+04 | 5,54E+04 | 5,54E+04 | 3,43E+04 | 4,12E+04 | 2,40E+04 | 2,40E+04 | 2,40E+04 | 1,50E+07 | 1,80E+06 | 3,50E+06 |
| P02765 | 2,09E+06 | 1,82E+06 | 4,39E+06 | 4,39E+06 | 2,72E+06 | 3,26E+06 | 1,90E+06 | 1,46E+06 | 2,92E+06 | 4,09E+06 | 2,63E+06 | 3,42E+06 |
| P02771 | 1,39E+10 | 1,21E+10 | 2,91E+10 | 2,91E+10 | 1,80E+10 | 2,16E+10 | 1,26E+10 | 9,69E+09 | 1,94E+10 | 2,71E+10 | 1,74E+10 | 2,27E+10 |
| P02792 | 2,46E+07 | 2,14E+07 | 5,17E+07 | 5,17E+07 | 3,20E+07 | 3,84E+07 | 2,24E+07 | 2,24E+07 | 2,24E+07 | 2,24E+07 | 2,24E+07 | 2,24E+07 |
| P02794 | 6,49E+06 | 5,64E+06 | 1,36E+07 | 1,36E+07 | 8,44E+06 | 1,01E+07 | 5,90E+06 | 4,54E+06 | 9,08E+06 | 1,27E+07 | 8,17E+06 | 1,06E+07 |
| P04083 | 2,00E+07 | 2,00E+07 | 2,00E+07 | 2,00E+07 | 3,10E+07 | 9,30E+07 | 5,00E+07 | 5,00E+07 | 5,00E+07 | 5,00E+07 | 5,00E+07 | 5,00E+07 |
| P04083 | 1,54E+06 | 1,34E+06 | 3,23E+06 | 3,23E+06 | 2,00E+06 | 2,40E+06 | 1,40E+06 | 1,40E+06 | 1,40E+06 | 1,50E+07 | 1,20E+06 | 2,20E+06 |
| P05091 | 2,09E+06 | 1,82E+06 | 4,39E+06 | 4,39E+06 | 2,72E+06 | 3,26E+06 | 1,90E+06 | 1,46E+06 | 2,92E+06 | 4,09E+06 | 2,63E+06 | 3,42E+06 |
| P05141 | 1,03E+07 | 8,99E+06 | 2,17E+07 | 2,17E+07 | 1,34E+07 | 1,61E+07 | 9,40E+06 | 9,40E+06 | 9,40E+06 | 9,40E+06 | 9,40E+06 | 9,40E+06 |
| P06744 | 2,09E+06 | 1,82E+06 | 4,39E+06 | 4,39E+06 | 2,72E+06 | 3,26E+06 | 1,90E+06 | 1,46E+06 | 2,92E+06 | 4,09E+06 | 2,63E+06 | 3,42E+06 |
| P07203 | 2,00E+07 | 2,00E+07 | 8,30E+06 | 2,49E+07 | 3,10E+07 | 9,30E+07 | 2,50E+07 | 2,50E+07 | 6,60E+07 | 6,60E+07 | 3,30E+07 | 6,60E+07 |
| P07900 | 2,09E+07 | 1,82E+07 | 4,39E+07 | 4,39E+07 | 2,72E+07 | 3,26E+07 | 1,90E+07 | 1,00E+03 | 1,90E+06 | 1,90E+07 | 1,00E+03 | 1,90E+06 |
| P07910 | 3,85E+07 | 3,35E+07 | 8,09E+07 | 8,09E+07 | 5,01E+07 | 6,01E+07 | 3,50E+07 | 3,50E+07 | 3,50E+07 | 3,50E+07 | 3,50E+07 | 3,50E+07 |
| P08238 | 1,65E+07 | 1,43E+07 | 3,47E+07 | 3,47E+07 | 2,15E+07 | 2,57E+07 | 1,50E+07 | 1,50E+07 | 1,50E+07 | 1,50E+07 | 1,50E+07 | 1,50E+07 |
| P08263 | 2,00E+07 | 2,00E+07 | 8,30E+06 | 2,49E+07 | 2,00E+07 | 2,00E+07 | 2,80E+07 | 2,80E+07 | 1,50E+07 | 3,00E+07 | 1,45E+08 | 1,45E+08 |
| P09382 | 2,09E+07 | 1,82E+07 | 4,39E+07 | 4,39E+07 | 2,72E+07 | 3,26E+07 | 1,90E+07 | 1,90E+07 | 1,90E+07 | 1,90E+07 | 1,90E+07 | 1,90E+07 |
| P0CG47 | 2,09E+07 | 1,82E+07 | 4,39E+07 | 4,39E+07 | 2,72E+07 | 3,26E+07 | 1,90E+07 | 1,90E+07 | 1,90E+07 | 1,90E+07 | 1,90E+07 | 1,90E+07 |
| P0CG48 | 1,36E+09 | 1,19E+09 | 2,86E+09 | 2,86E+09 | 1,77E+09 | 2,13E+09 | 1,24E+09 | 1,24E+09 | 1,24E+09 | 1,24E+09 | 1,24E+09 | 1,24E+09 |
| P0DMR1 | 1,98E+07 | 1,72E+07 | 4,16E+07 | 4,16E+07 | 2,57E+07 | 3,09E+07 | 1,80E+07 | 1,80E+07 | 1,80E+07 | 1,80E+07 | 1,80E+07 | 1,80E+07 |

| | | | | | | | | | | | | |
|---|---|---|---|---|---|---|---|---|---|---|---|---|
| P11279 | 1,65E+06 | 1,43E+06 | 3,47E+06 | 3,47E+06 | 2,15E+06 | 2,57E+06 | 1,50E+06 | 1,50E+06 | 1,50E+06 | 1,50E+06 | 1,50E+06 | 1,50E+06 |
| P12882 | 3,00E+07 | 6,00E+07 | 1,20E+07 | 3,60E+07 | 1,00E+07 | 3,00E+07 | 1,50E+07 | 1,50E+07 | 1,50E+07 | 1,50E+07 | 1,50E+07 | 1,50E+07 |
| P13473 | 1,65E+06 | 1,43E+06 | 3,47E+06 | 3,47E+06 | 2,15E+06 | 2,57E+06 | 1,50E+06 | 1,50E+06 | 1,50E+06 | 1,50E+06 | 1,50E+06 | 1,50E+06 |
| P14625 | 1,32E+07 | 1,15E+07 | 2,77E+07 | 2,77E+07 | 1,72E+07 | 2,06E+07 | 1,20E+07 | 1,20E+07 | 1,20E+07 | 1,20E+07 | 1,20E+07 | 1,20E+07 |
| P14780 | 4,40E+06 | 2,20E+06 | 3,80E+06 | 1,90E+06 | 5,00E+07 | 1,67E+07 | 2,30E+07 | 1,53E+07 | 1,50E+07 | 7,50E+06 | 2,25E+07 | 1,13E+07 |
| P15121 | 7,70E+06 | 6,70E+06 | 1,62E+07 | 1,62E+07 | 1,00E+07 | 1,20E+07 | 7,00E+06 | 7,00E+06 | 7,00E+06 | 7,00E+06 | 7,00E+06 | 7,00E+06 |
| P15531 | 2,09E+07 | 1,82E+07 | 4,39E+07 | 4,39E+07 | 2,72E+07 | 3,26E+07 | 1,90E+07 | 1,90E+07 | 1,90E+07 | 1,90E+07 | 1,90E+07 | 1,90E+07 |
| P16671 | 3,80E+06 | 1,90E+06 | 3,80E+06 | 1,90E+06 | 1,40E+07 | 4,67E+06 | 2,30E+07 | 1,53E+07 | 2,40E+07 | 1,20E+07 | 3,60E+07 | 1,80E+07 |
| P17174 | 3,63E+06 | 3,16E+06 | 7,62E+06 | 7,62E+06 | 4,72E+06 | 5,66E+06 | 3,30E+06 | 2,54E+06 | 5,08E+06 | 7,11E+06 | 4,57E+06 | 5,94E+06 |
| P17301 | 3,00E+07 | 6,00E+07 | 1,50E+07 | 4,50E+07 | 2,00E+07 | 6,00E+07 | 3,30E+07 | 3,30E+07 | 3,30E+07 | 3,30E+07 | 3,30E+07 | 3,30E+07 |
| P19338 | 2,09E+07 | 1,82E+07 | 4,39E+07 | 4,39E+07 | 2,72E+07 | 3,26E+07 | 1,90E+07 | 1,90E+07 | 1,90E+07 | 1,90E+07 | 1,90E+07 | 1,90E+07 |
| P19838 | 3,30E+07 | 3,30E+07 | 3,80E+06 | 1,90E+06 | 3,60E+07 | 1,20E+07 | 3,80E+06 | 3,80E+06 | 2,25E+07 | 2,25E+07 | 1,80E+07 | 9,00E+06 |
| P21796 | 4,40E+07 | 3,83E+07 | 9,24E+07 | 9,24E+07 | 5,72E+07 | 6,86E+07 | 4,00E+07 | 4,00E+07 | 4,00E+07 | 1,24E+08 | 1,24E+08 | 1,24E+08 |
| P22352 | 3,80E+06 | 3,80E+06 | 8,30E+06 | 2,49E+07 | 3,80E+06 | 1,14E+07 | 2,50E+08 | 2,50E+08 | 1,45E+08 | 1,45E+08 | 3,30E+07 | 6,60E+07 |
| P22392 | 4,40E+07 | 3,83E+07 | 9,24E+07 | 9,24E+07 | 5,72E+07 | 6,86E+07 | 4,00E+07 | 4,00E+07 | 4,00E+07 | 4,00E+07 | 4,00E+07 | 4,00E+07 |
| P23526 | 4,62E+06 | 4,02E+06 | 9,70E+06 | 9,70E+06 | 6,01E+06 | 7,21E+06 | 4,20E+06 | 3,23E+06 | 6,46E+06 | 9,05E+06 | 5,82E+06 | 7,56E+06 |
| P23528 | 3,96E+07 | 3,44E+07 | 8,32E+07 | 8,32E+07 | 5,15E+07 | 6,18E+07 | 3,60E+07 | 3,60E+07 | 3,60E+07 | 3,60E+07 | 3,60E+07 | 3,60E+07 |
| P25774 | 6,20E+07 | 3,10E+07 | 3,80E+06 | 3,80E+06 | 5,67E+06 | 5,67E+06 | 2,90E+07 | 1,93E+07 | 3,80E+06 | 3,80E+06 | 3,80E+06 | 3,80E+06 |
| P26641 | 2,64E+04 | 2,30E+04 | 5,54E+04 | 5,54E+04 | 3,43E+04 | 4,12E+04 | 2,40E+04 | 2,40E+04 | 2,40E+04 | 8,00E+06 | 2,40E+04 | 2,70E+06 |
| P27348 | 3,19E+07 | 2,77E+07 | 6,70E+07 | 6,70E+07 | 4,15E+07 | 4,98E+07 | 2,90E+07 | 2,90E+07 | 2,90E+07 | 2,90E+07 | 2,90E+07 | 2,90E+07 |
| P27487 | 6,00E+07 | 3,00E+07 | 3,80E+06 | 1,90E+06 | 3,50E+07 | 1,17E+07 | 3,00E+07 | 2,00E+07 | 2,30E+07 | 1,15E+07 | 3,45E+07 | 1,73E+07 |
| P27986 | 2,40E+07 | 4,80E+07 | 8,30E+06 | 2,49E+07 | 6,20E+07 | 1,86E+08 | 3,80E+06 | 7,60E+06 | 2,30E+07 | 9,20E+07 | 3,45E+07 | 1,38E+08 |
| P30101 | 6,86E+07 | 5,97E+07 | 1,44E+08 | 1,44E+08 | 8,92E+07 | 1,07E+08 | 6,24E+07 | 6,24E+07 | 6,24E+07 | 6,24E+07 | 6,24E+07 | 6,24E+07 |
| P30613 | 4,29E+07 | 3,73E+07 | 9,01E+07 | 9,01E+07 | 5,58E+07 | 6,69E+07 | 3,90E+07 | 3,90E+07 | 3,90E+07 | 3,90E+07 | 3,90E+07 | 3,90E+07 |
| P30838 | 2,09E+06 | 1,82E+06 | 4,39E+06 | 4,39E+06 | 2,72E+06 | 3,26E+06 | 1,90E+06 | 1,46E+06 | 2,92E+06 | 4,09E+06 | 2,63E+06 | 3,42E+06 |
| P31749 | 9,20E+06 | 1,84E+07 | 8,30E+06 | 2,49E+07 | 3,60E+05 | 1,08E+06 | 1,30E+07 | 7,80E+07 | 2,30E+07 | 4,60E+07 | 3,45E+07 | 6,90E+07 |
| P31751 | 1,00E+07 | 1,00E+07 | 3,10E+07 | 9,30E+07 | 3,80E+06 | 1,14E+07 | 6,00E+07 | 1,20E+08 | 1,20E+07 | 2,40E+07 | 1,80E+07 | 3,60E+07 |
| P35354 | 5,67E+06 | 5,67E+06 | 5,67E+06 | 5,67E+06 | 2,80E+07 | 9,33E+06 | 1,40E+07 | 1,40E+07 | 1,40E+07 | 1,40E+07 | 1,40E+07 | 1,40E+07 |
| P35613 | 4,62E+06 | 4,02E+06 | 9,70E+06 | 9,70E+06 | 6,01E+06 | 7,21E+06 | 4,20E+06 | 3,23E+06 | 6,46E+06 | 9,05E+06 | 5,82E+06 | 7,56E+06 |

| | | | | | | | | | | | | |
|---|---|---|---|---|---|---|---|---|---|---|---|---|
| P35669 | 2,40E+07 | 4,80E+07 | 8,30E+06 | 2,49E+07 | 2,00E+07 | 2,00E+07 | 3,80E+06 | 3,80E+06 | 3,80E+06 | 3,80E+06 | 3,80E+06 | 3,80E+06 |
| P37231 | 6,20E+07 | 6,20E+07 | 1,20E+07 | 3,60E+07 | 3,80E+06 | 1,14E+07 | 2,50E+07 | 2,50E+07 | 3,30E+07 | 6,60E+07 | 4,95E+07 | 9,90E+07 |
| P37837 | 2,42E+07 | 2,10E+07 | 5,08E+07 | 5,08E+07 | 3,15E+07 | 3,78E+07 | 2,20E+07 | 2,20E+07 | 2,20E+07 | 2,20E+07 | 2,20E+07 | 2,20E+07 |
| P40926 | 5,17E+06 | 4,50E+06 | 1,09E+07 | 1,09E+07 | 6,72E+06 | 8,07E+06 | 4,70E+06 | 4,70E+06 | 4,70E+06 | 2,90E+07 | 6,30E+06 | 1,20E+07 |
| P42330 | 3,63E+06 | 3,16E+06 | 7,62E+06 | 7,62E+06 | 4,72E+06 | 5,66E+06 | 3,30E+06 | 2,54E+06 | 5,08E+06 | 7,11E+06 | 4,57E+06 | 5,94E+06 |
| P45452 | 4,40E+06 | 2,20E+06 | 3,80E+06 | 3,80E+06 | 5,67E+06 | 5,67E+06 | 1,20E+07 | 8,00E+06 | 2,30E+07 | 1,15E+07 | 3,80E+06 | 3,80E+06 |
| P46940 | 2,86E+06 | 2,49E+06 | 6,01E+06 | 6,01E+06 | 3,72E+06 | 4,46E+06 | 2,60E+06 | 2,60E+06 | 2,60E+06 | 2,60E+06 | 2,60E+06 | 6,40E+06 |
| P48507 | 3,10E+07 | 6,20E+07 | 2,00E+07 | 2,00E+07 | 2,00E+07 | 2,00E+07 | 3,80E+06 | 3,80E+06 | 3,80E+06 | 3,80E+06 | 3,80E+06 | 3,80E+06 |
| P48637 | 3,10E+07 | 6,20E+07 | 8,30E+06 | 2,49E+07 | 6,00E+07 | 1,80E+08 | 3,80E+06 | 3,80E+06 | 3,80E+06 | 3,80E+06 | 3,80E+06 | 3,80E+06 |
| P48736 | 5,67E+06 | 5,67E+06 | 5,67E+06 | 5,67E+06 | 2,50E+08 | 8,33E+07 | 2,25E+07 | 2,25E+07 | 2,90E+07 | 2,90E+07 | 4,35E+07 | 2,18E+07 |
| P51148 | 3,19E+07 | 2,77E+07 | 6,70E+07 | 6,70E+07 | 4,15E+07 | 4,98E+07 | 2,90E+07 | 2,90E+07 | 2,90E+07 | 2,90E+07 | 2,90E+07 | 2,90E+07 |
| P52209 | 2,09E+06 | 1,82E+06 | 4,39E+06 | 4,39E+06 | 2,72E+06 | 3,26E+06 | 1,90E+06 | 1,46E+06 | 2,92E+06 | 4,09E+06 | 2,63E+06 | 3,42E+06 |
| P55085 | 5,67E+06 | 5,67E+06 | 5,67E+06 | 5,67E+06 | 2,30E+07 | 7,67E+06 | 5,00E+07 | 5,00E+07 | 5,00E+07 | 5,00E+07 | 5,00E+07 | 5,00E+07 |
| P59998 | 1,39E+10 | 1,21E+10 | 2,91E+10 | 2,91E+10 | 1,80E+10 | 2,16E+10 | 1,26E+10 | 9,69E+09 | 1,94E+10 | 2,71E+10 | 1,74E+10 | 2,27E+10 |
| P60709 | 3,00E+07 | 6,00E+07 | 1,50E+07 | 4,50E+07 | 2,00E+07 | 6,00E+07 | 3,30E+07 | 3,30E+07 | 3,30E+07 | 3,30E+07 | 3,30E+07 | 3,30E+07 |
| P60842 | 7,92E+05 | 6,89E+05 | 1,66E+06 | 1,66E+06 | 1,03E+06 | 1,24E+06 | 7,20E+05 | 7,20E+05 | 7,20E+05 | 5,30E+06 | 5,30E+06 | 5,30E+06 |
| P61204 | 2,86E+06 | 2,49E+06 | 6,01E+06 | 6,01E+06 | 3,72E+06 | 4,46E+06 | 2,60E+06 | 2,60E+06 | 2,60E+06 | 3,00E+06 | 3,60E+05 | 2,60E+06 |
| P61604 | 1,39E+10 | 1,21E+10 | 2,91E+10 | 2,91E+10 | 1,80E+10 | 2,16E+10 | 1,26E+10 | 9,69E+09 | 1,94E+10 | 2,71E+10 | 1,74E+10 | 2,27E+10 |
| P61981 | 4,95E+06 | 4,30E+06 | 1,04E+07 | 1,04E+07 | 6,44E+06 | 7,72E+06 | 4,50E+06 | 3,46E+06 | 6,92E+06 | 9,69E+06 | 6,23E+06 | 8,10E+06 |
| P62244 | 2,09E+07 | 1,82E+07 | 4,39E+07 | 4,39E+07 | 2,72E+07 | 3,26E+07 | 1,90E+07 | 1,90E+07 | 1,90E+07 | 1,90E+07 | 1,90E+07 | 1,90E+07 |
| P62249 | 6,60E+06 | 5,74E+06 | 1,39E+07 | 1,39E+07 | 8,58E+06 | 1,03E+07 | 6,00E+06 | 6,00E+06 | 6,00E+06 | 6,00E+06 | 6,00E+06 | 6,00E+06 |
| P62258 | 5,50E+06 | 4,78E+06 | 1,16E+07 | 1,16E+07 | 7,15E+06 | 8,58E+06 | 5,00E+06 | 5,00E+06 | 5,00E+06 | 1,24E+08 | 1,24E+08 | 1,24E+08 |
| P62314 | 2,42E+06 | 2,10E+06 | 5,08E+06 | 5,08E+06 | 3,15E+06 | 3,78E+06 | 2,20E+06 | 2,20E+06 | 2,20E+06 | 5,70E+06 | 2,60E+10 | 6,26E+09 |
| P62318 | 3,63E+06 | 3,16E+06 | 7,62E+06 | 7,62E+06 | 4,72E+06 | 5,66E+06 | 3,30E+06 | 2,54E+06 | 5,08E+06 | 7,11E+06 | 4,57E+06 | 5,94E+06 |
| P62826 | 2,42E+07 | 2,10E+07 | 5,08E+07 | 5,08E+07 | 3,15E+07 | 3,78E+07 | 2,20E+07 | 2,30E+06 | 2,00E+06 | 2,20E+07 | 2,30E+06 | 2,00E+06 |
| P62937 | 1,03E+08 | 8,99E+07 | 2,17E+08 | 2,17E+08 | 1,34E+08 | 1,61E+08 | 9,40E+07 | 9,40E+07 | 9,40E+07 | 9,40E+07 | 9,40E+07 | 9,40E+07 |
| P62979 | 1,36E+09 | 1,19E+09 | 2,86E+09 | 2,86E+09 | 1,77E+09 | 2,13E+09 | 1,24E+09 | 1,24E+09 | 1,24E+09 | 1,24E+09 | 1,24E+09 | 1,24E+09 |
| P63104 | 4,62E+06 | 4,02E+06 | 9,70E+06 | 9,70E+06 | 6,01E+06 | 7,21E+06 | 4,20E+06 | 4,20E+06 | 4,20E+06 | 1,40E+07 | 3,20E+06 | 9,70E+05 |
| P63241 | 2,86E+06 | 2,49E+06 | 6,01E+06 | 6,01E+06 | 3,72E+06 | 4,46E+06 | 2,60E+06 | 2,60E+06 | 2,60E+06 | 5,90E+06 | 4,40E+06 | 3,40E+06 |

| | | | | | | | | | | | | |
|---|---|---|---|---|---|---|---|---|---|---|---|---|
| P68104 | 2,86E+06 | 2,49E+06 | 6,01E+06 | 6,01E+06 | 3,72E+06 | 4,46E+06 | 2,60E+06 | 2,60E+06 | 2,60E+06 | 1,26E+10 | 2,60E+06 | 2,60E+06 |
| P68431 | 6,86E+07 | 5,97E+07 | 1,44E+08 | 1,44E+08 | 8,92E+07 | 1,07E+08 | 6,24E+07 | 6,24E+07 | 6,24E+07 | 6,24E+07 | 6,24E+07 | 6,24E+07 |
| P84077 | 4,62E+06 | 4,02E+06 | 9,70E+06 | 9,70E+06 | 6,01E+06 | 7,21E+06 | 4,20E+06 | 3,23E+06 | 6,46E+06 | 9,05E+06 | 5,82E+06 | 7,56E+06 |
| P84243 | 6,86E+07 | 5,97E+07 | 1,44E+08 | 1,44E+08 | 8,92E+07 | 1,07E+08 | 6,24E+07 | 6,24E+07 | 6,24E+07 | 6,24E+07 | 6,24E+07 | 6,24E+07 |
| Q02388 | 1,90E+06 | 3,80E+06 | 3,10E+07 | 9,30E+07 | 2,00E+07 | 6,00E+07 | 6,00E+07 | 1,20E+08 | 2,40E+07 | 4,80E+07 | 3,60E+07 | 7,20E+07 |
| Q02878 | 9,46E+06 | 8,23E+06 | 1,99E+07 | 1,99E+07 | 1,23E+07 | 1,48E+07 | 8,60E+06 | 8,60E+06 | 8,60E+06 | 8,60E+06 | 8,60E+06 | 8,60E+06 |
| Q03181 | 6,00E+07 | 6,00E+07 | 1,30E+07 | 3,90E+07 | 3,80E+06 | 1,14E+07 | 2,30E+07 | 2,30E+07 | 3,30E+07 | 6,60E+07 | 4,95E+07 | 9,90E+07 |
| Q04206 | 3,60E+05 | 3,60E+05 | 1,50E+07 | 4,50E+07 | 3,80E+06 | 1,14E+07 | 1,50E+08 | 1,50E+08 | 1,50E+08 | 1,50E+08 | 1,50E+08 | 1,50E+08 |
| Q04206 | 3,30E+07 | 3,30E+07 | 3,10E+07 | 1,55E+07 | 2,60E+08 | 8,67E+07 | 1,40E+07 | 1,40E+07 | 1,40E+07 | 1,40E+07 | 1,40E+07 | 1,40E+07 |
| Q05397 | 6,10E+07 | 1,22E+08 | 1,20E+07 | 3,60E+07 | 4,40E+06 | 1,32E+07 | 6,10E+07 | 2,44E+08 | 1,50E+07 | 3,00E+07 | 2,25E+07 | 4,50E+07 |
| Q06828 | 1,30E+07 | 6,50E+06 | 3,80E+06 | 3,80E+06 | 5,67E+06 | 5,67E+06 | 5,00E+07 | 5,00E+07 | 5,00E+07 | 5,00E+07 | 5,00E+07 | 5,00E+07 |
| Q0EFC9 | 2,20E+07 | 1,91E+07 | 4,62E+07 | 4,62E+07 | 2,86E+07 | 3,43E+07 | 2,00E+07 | 2,00E+07 | 2,00E+07 | 2,00E+07 | 2,00E+07 | 2,00E+07 |
| Q0QEN7 | 1,98E+07 | 1,72E+07 | 4,16E+07 | 4,16E+07 | 2,57E+07 | 3,09E+07 | 1,80E+07 | 1,80E+07 | 1,80E+07 | 1,80E+07 | 1,80E+07 | 1,80E+07 |
| Q0QET7 | 1,65E+08 | 1,43E+08 | 3,47E+08 | 3,47E+08 | 2,15E+08 | 2,57E+08 | 1,50E+08 | 1,50E+08 | 1,50E+08 | 1,50E+08 | 1,50E+08 | 1,50E+08 |
| Q0QF37 | 5,17E+06 | 4,50E+06 | 1,09E+07 | 1,09E+07 | 6,72E+06 | 8,07E+06 | 4,70E+06 | 4,70E+06 | 4,70E+06 | 2,90E+07 | 6,30E+06 | 1,20E+07 |
| Q0VAC0 | 3,63E+06 | 3,16E+06 | 7,62E+06 | 7,62E+06 | 4,72E+06 | 5,66E+06 | 3,30E+06 | 2,54E+06 | 5,08E+06 | 7,11E+06 | 4,57E+06 | 5,94E+06 |
| Q0VAS5 | 1,43E+06 | 1,24E+06 | 3,00E+06 | 3,00E+06 | 1,86E+06 | 2,23E+06 | 1,30E+06 | 1,30E+06 | 1,30E+06 | 4,20E+08 | 5,20E+07 | 8,80E+06 |
| Q12884 | 3,60E+05 | 1,80E+05 | 3,80E+06 | 1,90E+06 | 3,50E+07 | 1,17E+07 | 5,00E+07 | 5,00E+07 | 5,00E+07 | 5,00E+07 | 5,00E+07 | 5,00E+07 |
| Q13747 | 1,39E+10 | 1,21E+10 | 2,91E+10 | 2,91E+10 | 1,80E+10 | 2,16E+10 | 1,26E+10 | 9,69E+09 | 1,94E+10 | 2,71E+10 | 1,74E+10 | 2,27E+10 |
| Q13838 | 3,63E+06 | 3,16E+06 | 7,62E+06 | 7,62E+06 | 4,72E+06 | 5,66E+06 | 3,30E+06 | 3,30E+06 | 3,30E+06 | 3,30E+06 | 3,30E+06 | 3,30E+06 |
| Q14240 | 7,92E+05 | 6,89E+05 | 1,66E+06 | 1,66E+06 | 1,03E+06 | 1,24E+06 | 7,20E+05 | 7,20E+05 | 7,20E+05 | 5,30E+06 | 5,30E+06 | 5,30E+06 |
| Q14289 | 2,00E+07 | 2,00E+07 | 8,30E+06 | 2,49E+07 | 1,30E+07 | 3,90E+07 | 3,80E+06 | 1,52E+07 | 3,00E+07 | 6,00E+07 | 4,50E+07 | 9,00E+07 |
| Q14457 | 4,20E+07 | 8,40E+07 | 1,30E+07 | 3,90E+07 | 4,40E+06 | 1,32E+07 | 1,50E+07 | 1,50E+07 | 1,50E+07 | 1,50E+07 | 1,50E+07 | 1,50E+07 |
| Q15084 | 3,08E+06 | 2,68E+06 | 6,47E+06 | 6,47E+06 | 4,00E+06 | 4,80E+06 | 2,80E+06 | 2,80E+06 | 2,80E+06 | 2,00E+07 | 4,20E+05 | 3,40E+06 |
| Q15139 | 5,67E+06 | 5,67E+06 | 5,67E+06 | 5,67E+06 | 2,50E+07 | 8,33E+06 | 2,25E+07 | 2,25E+07 | 2,90E+07 | 2,90E+07 | 4,35E+07 | 2,18E+07 |
| Q15582 | 6,10E+07 | 3,05E+07 | 3,80E+06 | 3,80E+06 | 5,67E+06 | 5,67E+06 | 1,40E+07 | 1,40E+07 | 1,40E+07 | 1,40E+07 | 1,40E+07 | 1,40E+07 |
| Q15759 | 2,00E+07 | 2,00E+07 | 8,30E+06 | 2,49E+07 | 3,80E+06 | 1,14E+07 | 2,50E+08 | 2,50E+08 | 2,90E+07 | 1,45E+08 | 4,35E+07 | 2,18E+08 |
| Q16577 | 2,86E+06 | 2,49E+06 | 6,01E+06 | 6,01E+06 | 3,72E+06 | 4,46E+06 | 2,60E+06 | 2,60E+06 | 2,60E+06 | 6,80E+06 | 2,60E+06 | 2,60E+06 |
| Q16695 | 6,86E+07 | 5,97E+07 | 1,44E+08 | 1,44E+08 | 8,92E+07 | 1,07E+08 | 6,24E+07 | 6,24E+07 | 6,24E+07 | 6,24E+07 | 6,24E+07 | 6,24E+07 |

| | | | | | | | | | | | | |
|---|---|---|---|---|---|---|---|---|---|---|---|---|
| Q16715 | 4,29E+07 | 3,73E+07 | 9,01E+07 | 9,01E+07 | 5,58E+07 | 6,69E+07 | 3,90E+07 | 3,90E+07 | 3,90E+07 | 3,90E+07 | 3,90E+07 | 3,90E+07 |
| Q16716 | 4,29E+07 | 3,73E+07 | 9,01E+07 | 9,01E+07 | 5,58E+07 | 6,69E+07 | 3,90E+07 | 3,90E+07 | 3,90E+07 | 3,90E+07 | 3,90E+07 | 3,90E+07 |
| Q1RMG2 | 4,62E+06 | 4,02E+06 | 9,70E+06 | 9,70E+06 | 6,01E+06 | 7,21E+06 | 4,20E+06 | 3,23E+06 | 6,46E+06 | 9,05E+06 | 5,82E+06 | 7,56E+06 |
| Q20679 | 4,20E+07 | 8,40E+07 | 2,00E+07 | 2,00E+07 | 2,00E+07 | 2,00E+07 | 1,50E+07 | 1,50E+07 | 1,50E+07 | 1,50E+07 | 1,50E+07 | 1,50E+07 |
| Q2QD09 | 2,64E+08 | 2,30E+08 | 5,54E+08 | 5,54E+08 | 3,43E+08 | 4,12E+08 | 2,40E+08 | 2,40E+08 | 2,40E+08 | 2,40E+08 | 2,40E+08 | 2,40E+08 |
| Q2TU84 | 2,09E+06 | 1,82E+06 | 4,39E+06 | 4,39E+06 | 2,72E+06 | 3,26E+06 | 1,90E+06 | 1,46E+06 | 2,92E+06 | 4,09E+06 | 2,63E+06 | 3,42E+06 |
| Q2VPJ6 | 2,09E+07 | 1,82E+07 | 4,39E+07 | 4,39E+07 | 2,72E+07 | 3,26E+07 | 1,90E+07 | 1,00E+03 | 1,90E+06 | 1,90E+07 | 1,00E+03 | 1,90E+06 |
| Q32Q12 | 4,40E+07 | 3,83E+07 | 9,24E+07 | 9,24E+07 | 5,72E+07 | 6,86E+07 | 4,00E+07 | 4,00E+07 | 4,00E+07 | 4,00E+07 | 4,00E+07 | 4,00E+07 |
| Q3BDU5 | 2,86E+06 | 2,49E+06 | 6,01E+06 | 6,01E+06 | 3,72E+06 | 4,46E+06 | 2,60E+06 | 2,60E+06 | 2,60E+06 | 9,00E+06 | 9,60E+05 | 2,60E+06 |
| Q3C1U4 | 1,30E+07 | 1,30E+07 | 8,30E+06 | 2,49E+07 | 3,80E+06 | 1,14E+07 | 1,50E+08 | 1,50E+08 | 1,50E+08 | 1,50E+08 | 1,50E+08 | 1,50E+08 |
| Q3MIH3 | 1,36E+09 | 1,19E+09 | 2,86E+09 | 2,86E+09 | 1,77E+09 | 2,13E+09 | 1,24E+09 | 1,24E+09 | 1,24E+09 | 1,24E+09 | 1,24E+09 | 1,24E+09 |
| Q4JM47 | 4,73E+06 | 4,11E+06 | 9,93E+06 | 9,93E+06 | 6,15E+06 | 7,38E+06 | 4,30E+06 | 4,30E+06 | 4,30E+06 | 1,60E+07 | 2,50E+06 | 2,60E+06 |
| Q504U3 | 2,46E+07 | 2,14E+07 | 5,17E+07 | 5,17E+07 | 3,20E+07 | 3,84E+07 | 2,24E+07 | 2,24E+07 | 2,24E+07 | 2,80E+07 | 2,80E+07 | 2,80E+07 |
| Q53G64 | 2,09E+06 | 1,82E+06 | 4,39E+06 | 4,39E+06 | 2,72E+06 | 3,26E+06 | 1,90E+06 | 1,46E+06 | 2,92E+06 | 4,09E+06 | 2,63E+06 | 3,42E+06 |
| Q53G71 | 3,85E+06 | 3,35E+06 | 8,09E+06 | 8,09E+06 | 5,01E+06 | 6,01E+06 | 3,50E+06 | 3,50E+06 | 3,50E+06 | 3,50E+06 | 3,50E+06 | 3,50E+06 |
| Q53G85 | 2,86E+06 | 2,49E+06 | 6,01E+06 | 6,01E+06 | 3,72E+06 | 4,46E+06 | 2,60E+06 | 2,60E+06 | 2,60E+06 | 9,60E+06 | 2,60E+06 | 2,60E+06 |
| Q53GA1 | 2,86E+06 | 2,49E+06 | 6,01E+06 | 6,01E+06 | 3,72E+06 | 4,46E+06 | 2,60E+06 | 2,60E+06 | 2,60E+06 | 6,80E+06 | 2,60E+06 | 2,60E+06 |
| Q53GE9 | 2,86E+06 | 2,49E+06 | 6,01E+06 | 6,01E+06 | 3,72E+06 | 4,46E+06 | 2,60E+06 | 2,60E+06 | 2,60E+06 | 1,40E+07 | 2,60E+06 | 2,60E+06 |
| Q53HM9 | 2,86E+06 | 2,49E+06 | 6,01E+06 | 6,01E+06 | 3,72E+06 | 4,46E+06 | 2,60E+06 | 2,60E+06 | 2,60E+06 | 1,26E+10 | 2,60E+06 | 2,60E+06 |
| Q53HQ7 | 2,86E+06 | 2,49E+06 | 6,01E+06 | 6,01E+06 | 3,72E+06 | 4,46E+06 | 2,60E+06 | 2,60E+06 | 2,60E+06 | 1,26E+10 | 2,60E+06 | 2,60E+06 |
| Q53HR5 | 2,86E+06 | 2,49E+06 | 6,01E+06 | 6,01E+06 | 3,72E+06 | 4,46E+06 | 2,60E+06 | 2,60E+06 | 2,60E+06 | 1,26E+10 | 2,60E+06 | 2,60E+06 |
| Q53HU8 | 2,64E+04 | 2,30E+04 | 5,54E+04 | 5,54E+04 | 3,43E+04 | 4,12E+04 | 2,40E+04 | 2,40E+04 | 2,40E+04 | 2,40E+04 | 2,40E+04 | 2,40E+04 |
| Q53HW2 | 6,49E+06 | 5,64E+06 | 1,36E+07 | 1,36E+07 | 8,44E+06 | 1,01E+07 | 5,90E+06 | 4,54E+06 | 9,08E+06 | 1,27E+07 | 8,17E+06 | 1,06E+07 |
| Q549N0 | 4,07E+06 | 3,54E+06 | 8,55E+06 | 8,55E+06 | 5,29E+06 | 6,35E+06 | 3,70E+06 | 3,70E+06 | 3,70E+06 | 2,40E+04 | 4,00E+06 | 2,40E+04 |
| Q54A51 | 2,86E+07 | 2,49E+07 | 6,01E+07 | 6,01E+07 | 3,72E+07 | 4,46E+07 | 2,60E+07 | 2,00E+07 | 4,00E+07 | 5,60E+07 | 3,60E+07 | 4,68E+07 |
| Q569J8 | 1,98E+07 | 1,72E+07 | 4,16E+07 | 4,16E+07 | 2,57E+07 | 3,09E+07 | 1,80E+07 | 1,80E+07 | 1,80E+07 | 1,80E+07 | 1,80E+07 | 1,80E+07 |
| Q59E93 | 1,65E+06 | 1,43E+06 | 3,47E+06 | 3,47E+06 | 2,15E+06 | 2,57E+06 | 1,50E+06 | 1,50E+06 | 1,50E+06 | 1,50E+06 | 1,50E+06 | 1,50E+06 |
| Q59EI9 | 8,47E+06 | 7,37E+06 | 1,78E+07 | 1,78E+07 | 1,10E+07 | 1,32E+07 | 7,70E+06 | 7,70E+06 | 7,70E+06 | 7,70E+06 | 7,70E+06 | 7,70E+06 |
| Q59EJ0 | 2,09E+06 | 1,82E+06 | 4,39E+06 | 4,39E+06 | 2,72E+06 | 3,26E+06 | 1,90E+06 | 1,46E+06 | 2,92E+06 | 4,09E+06 | 2,63E+06 | 3,42E+06 |

| | | | | | | | | | | | | |
|---|---|---|---|---|---|---|---|---|---|---|---|---|
| Q59EL5 | 3,19E+07 | 2,77E+07 | 6,70E+07 | 6,70E+07 | 4,15E+07 | 4,98E+07 | 2,90E+07 | 2,90E+07 | 2,90E+07 | 2,90E+07 | 2,90E+07 | 2,90E+07 |
| Q59EM9 | 1,36E+09 | 1,19E+09 | 2,86E+09 | 2,86E+09 | 1,77E+09 | 2,13E+09 | 1,24E+09 | 1,24E+09 | 1,24E+09 | 1,24E+09 | 1,24E+09 | 1,24E+09 |
| Q59F68 | 6,16E+06 | 5,36E+06 | 1,29E+07 | 1,29E+07 | 8,01E+06 | 9,61E+06 | 5,60E+06 | 5,60E+06 | 5,60E+06 | 5,60E+06 | 5,60E+06 | 5,60E+06 |
| Q59F85 | 2,09E+06 | 1,82E+06 | 4,39E+06 | 4,39E+06 | 2,72E+06 | 3,26E+06 | 1,90E+06 | 1,46E+06 | 2,92E+06 | 4,09E+06 | 2,63E+06 | 3,42E+06 |
| Q59FC6 | 1,32E+07 | 1,15E+07 | 2,77E+07 | 2,77E+07 | 1,72E+07 | 2,06E+07 | 1,20E+07 | 1,20E+07 | 1,20E+07 | 1,20E+07 | 1,20E+07 | 1,20E+07 |
| Q59G24 | 4,95E+06 | 4,30E+06 | 1,04E+07 | 1,04E+07 | 6,44E+06 | 7,72E+06 | 4,50E+06 | 3,46E+06 | 6,92E+06 | 9,69E+06 | 6,23E+06 | 8,10E+06 |
| Q59G92 | 2,09E+06 | 1,82E+06 | 4,39E+06 | 4,39E+06 | 2,72E+06 | 3,26E+06 | 1,90E+06 | 1,90E+06 | 1,90E+06 | 1,90E+06 | 1,90E+06 | 1,90E+06 |
| Q5CAQ5 | 1,32E+07 | 1,15E+07 | 2,77E+07 | 2,77E+07 | 1,72E+07 | 2,06E+07 | 1,20E+07 | 1,20E+07 | 1,20E+07 | 1,20E+07 | 1,20E+07 | 1,20E+07 |
| Q5EFE5 | 2,09E+06 | 1,82E+06 | 4,39E+06 | 4,39E+06 | 2,72E+06 | 3,26E+06 | 1,90E+06 | 1,46E+06 | 2,92E+06 | 4,09E+06 | 2,63E+06 | 3,42E+06 |
| Q5EFE6 | 2,09E+06 | 1,82E+06 | 4,39E+06 | 4,39E+06 | 2,72E+06 | 3,26E+06 | 1,90E+06 | 1,46E+06 | 2,92E+06 | 4,09E+06 | 2,63E+06 | 3,42E+06 |
| Q5I6Y5 | 6,49E+06 | 5,64E+06 | 1,36E+07 | 1,36E+07 | 8,44E+06 | 1,01E+07 | 5,90E+06 | 4,54E+06 | 9,08E+06 | 1,27E+07 | 8,17E+06 | 1,06E+07 |
| Q5JVS8 | 2,86E+06 | 2,49E+06 | 6,01E+06 | 6,01E+06 | 3,72E+06 | 4,46E+06 | 2,60E+06 | 2,60E+06 | 2,60E+06 | 2,60E+06 | 2,60E+06 | 2,60E+06 |
| Q5PY61 | 1,36E+09 | 1,19E+09 | 2,86E+09 | 2,86E+09 | 1,77E+09 | 2,13E+09 | 1,24E+09 | 1,24E+09 | 1,24E+09 | 1,24E+09 | 1,24E+09 | 1,24E+09 |
| Q5R206 | 4,84E+05 | 4,21E+05 | 1,02E+06 | 1,02E+06 | 6,29E+05 | 7,55E+05 | 4,40E+05 | 4,40E+05 | 4,40E+05 | 1,60E+06 | 2,60E+06 | 2,80E+06 |
| Q5R207 | 2,86E+06 | 2,49E+06 | 6,01E+06 | 6,01E+06 | 3,72E+06 | 4,46E+06 | 2,60E+06 | 2,60E+06 | 9,80E+05 | 9,80E+05 | 2,80E+06 | 2,80E+06 |
| Q5R208 | 4,84E+05 | 4,21E+05 | 1,02E+06 | 1,02E+06 | 6,29E+05 | 7,55E+05 | 4,40E+05 | 4,40E+05 | 4,40E+05 | 1,60E+06 | 2,60E+06 | 2,80E+06 |
| Q5R209 | 4,84E+05 | 4,21E+05 | 1,02E+06 | 1,02E+06 | 6,29E+05 | 7,55E+05 | 4,40E+05 | 4,40E+05 | 4,40E+05 | 9,80E+05 | 2,60E+06 | 2,80E+06 |
| Q5R210 | 4,84E+05 | 4,21E+05 | 1,02E+06 | 1,02E+06 | 6,29E+05 | 7,55E+05 | 4,40E+05 | 4,40E+05 | 4,40E+05 | 1,60E+06 | 2,60E+06 | 2,80E+06 |
| Q5RKT7 | 1,36E+09 | 1,19E+09 | 2,86E+09 | 2,86E+09 | 1,77E+09 | 2,13E+09 | 1,24E+09 | 1,24E+09 | 1,24E+09 | 1,24E+09 | 1,24E+09 | 1,24E+09 |
| Q5STU3 | 3,63E+06 | 3,16E+06 | 7,62E+06 | 7,62E+06 | 4,72E+06 | 5,66E+06 | 3,30E+06 | 3,30E+06 | 3,30E+06 | 3,30E+06 | 3,30E+06 | 3,30E+06 |
| Q5TA01 | 6,86E+07 | 5,97E+07 | 1,44E+08 | 1,44E+08 | 8,92E+07 | 1,07E+08 | 6,24E+07 | 6,24E+07 | 6,24E+07 | 6,24E+07 | 6,24E+07 | 6,24E+07 |
| Q5TA02 | 2,42E+05 | 2,10E+05 | 5,08E+05 | 5,08E+05 | 3,15E+05 | 3,78E+05 | 2,20E+05 | 2,20E+05 | 2,20E+05 | 1,20E+07 | 1,80E+06 | 1,80E+06 |
| Q5TCI8 | 2,64E+04 | 2,30E+04 | 5,54E+04 | 5,54E+04 | 3,43E+04 | 4,12E+04 | 2,40E+04 | 2,40E+04 | 2,40E+04 | 1,50E+07 | 2,40E+06 | 3,50E+06 |
| Q5UGI3 | 1,36E+09 | 1,19E+09 | 2,86E+09 | 2,86E+09 | 1,77E+09 | 2,13E+09 | 1,24E+09 | 1,24E+09 | 1,24E+09 | 1,24E+09 | 1,24E+09 | 1,24E+09 |
| Q5VTE0 | 2,86E+06 | 2,49E+06 | 6,01E+06 | 6,01E+06 | 3,72E+06 | 4,46E+06 | 2,60E+06 | 2,60E+06 | 2,60E+06 | 1,26E+10 | 2,60E+06 | 2,60E+06 |
| Q6B823 | 2,42E+08 | 2,10E+08 | 5,08E+08 | 5,08E+08 | 3,15E+08 | 3,78E+08 | 2,20E+08 | 2,20E+08 | 2,20E+08 | 2,20E+08 | 2,20E+08 | 2,20E+08 |
| Q6FHZ0 | 5,17E+06 | 4,50E+06 | 1,09E+07 | 1,09E+07 | 6,72E+06 | 8,07E+06 | 4,70E+06 | 4,70E+06 | 4,70E+06 | 2,90E+07 | 6,30E+06 | 1,20E+07 |
| Q6I9V5 | 8,47E+06 | 7,37E+06 | 1,78E+07 | 1,78E+07 | 1,10E+07 | 1,32E+07 | 7,70E+06 | 7,70E+06 | 7,70E+06 | 7,70E+06 | 7,70E+06 | 7,70E+06 |
| Q6IPF2 | 2,09E+06 | 1,82E+06 | 4,39E+06 | 4,39E+06 | 2,72E+06 | 3,26E+06 | 1,90E+06 | 1,46E+06 | 2,92E+06 | 4,09E+06 | 2,63E+06 | 3,42E+06 |

| | | | | | | | | | | | | |
|---|---|---|---|---|---|---|---|---|---|---|---|---|
| Q6IPN6 | 2,86E+06 | 2,49E+06 | 6,01E+06 | 6,01E+06 | 3,72E+06 | 4,46E+06 | 2,60E+06 | 2,60E+06 | 2,60E+06 | 1,26E+10 | 2,60E+06 | 2,60E+06 |
| Q6IPT9 | 2,86E+06 | 2,49E+06 | 6,01E+06 | 6,01E+06 | 3,72E+06 | 4,46E+06 | 2,60E+06 | 2,60E+06 | 2,60E+06 | 1,26E+10 | 2,60E+06 | 2,60E+06 |
| Q6LE88 | 2,64E+04 | 2,30E+04 | 5,54E+04 | 5,54E+04 | 3,43E+04 | 4,12E+04 | 2,40E+04 | 2,40E+04 | 2,40E+04 | 2,40E+04 | 1,50E+06 | 2,40E+04 |
| Q6NS36 | 6,49E+06 | 5,64E+06 | 1,36E+07 | 1,36E+07 | 8,44E+06 | 1,01E+07 | 5,90E+06 | 4,54E+06 | 9,08E+06 | 1,27E+07 | 8,17E+06 | 1,06E+07 |
| Q6NVC0 | 1,03E+07 | 8,99E+06 | 2,17E+07 | 2,17E+07 | 1,34E+07 | 1,61E+07 | 9,40E+06 | 9,40E+06 | 9,40E+06 | 9,40E+06 | 9,40E+06 | 9,40E+06 |
| Q6NZ44 | 1,98E+06 | 1,72E+06 | 4,16E+06 | 4,16E+06 | 2,57E+06 | 3,09E+06 | 1,80E+06 | 1,38E+06 | 2,77E+06 | 3,88E+06 | 2,49E+06 | 3,24E+06 |
| Q6P1N4 | 2,86E+06 | 2,49E+06 | 6,01E+06 | 6,01E+06 | 3,72E+06 | 4,46E+06 | 2,60E+06 | 2,60E+06 | 2,60E+06 | 2,60E+06 | 2,60E+06 | 6,40E+06 |
| Q6PJ43 | 7,92E+05 | 6,89E+05 | 1,66E+06 | 1,66E+06 | 1,03E+06 | 1,24E+06 | 7,20E+05 | 7,20E+05 | 7,20E+05 | 7,20E+05 | 7,20E+05 | 7,20E+05 |
| Q6PK50 | 1,65E+07 | 1,43E+07 | 3,47E+07 | 3,47E+07 | 2,15E+07 | 2,57E+07 | 1,50E+07 | 1,50E+07 | 1,50E+07 | 1,50E+07 | 1,50E+07 | 1,50E+07 |
| Q6PKA6 | 2,09E+06 | 1,82E+06 | 4,39E+06 | 4,39E+06 | 2,72E+06 | 3,26E+06 | 1,90E+06 | 1,46E+06 | 2,92E+06 | 4,09E+06 | 2,63E+06 | 3,42E+06 |
| Q6PKD2 | 1,98E+07 | 1,72E+07 | 4,16E+07 | 4,16E+07 | 2,57E+07 | 3,09E+07 | 1,80E+07 | 1,80E+07 | 1,80E+07 | 1,80E+07 | 1,80E+07 | 1,80E+07 |
| Q6S4P3 | 2,64E+04 | 2,30E+04 | 5,54E+04 | 5,54E+04 | 3,43E+04 | 4,12E+04 | 2,40E+04 | 2,40E+04 | 2,40E+04 | 2,40E+04 | 2,40E+04 | 2,30E+06 |
| Q6TXQ4 | 6,86E+07 | 5,97E+07 | 1,44E+08 | 1,44E+08 | 8,92E+07 | 1,07E+08 | 6,24E+07 | 6,24E+07 | 6,24E+07 | 6,24E+07 | 6,24E+07 | 6,24E+07 |
| Q6ZR44 | 6,49E+06 | 5,64E+06 | 1,36E+07 | 1,36E+07 | 8,44E+06 | 1,01E+07 | 5,90E+06 | 4,54E+06 | 9,08E+06 | 1,27E+07 | 8,17E+06 | 1,06E+07 |
| Q6ZS99 | 2,09E+07 | 1,82E+07 | 4,39E+07 | 4,39E+07 | 2,72E+07 | 3,26E+07 | 1,90E+07 | 1,90E+07 | 1,90E+07 | 1,90E+07 | 1,90E+07 | 1,90E+07 |
| Q71DI3 | 2,09E+07 | 1,82E+07 | 4,39E+07 | 4,39E+07 | 2,72E+07 | 3,26E+07 | 1,90E+07 | 1,90E+07 | 1,90E+07 | 1,90E+07 | 1,90E+07 | 1,90E+07 |
| Q71V99 | 1,03E+08 | 8,99E+07 | 2,17E+08 | 2,17E+08 | 1,34E+08 | 1,61E+08 | 9,40E+07 | 9,40E+07 | 9,40E+07 | 9,40E+07 | 9,40E+07 | 9,40E+07 |
| Q75MT9 | 5,17E+06 | 4,50E+06 | 1,09E+07 | 1,09E+07 | 6,72E+06 | 8,07E+06 | 4,70E+06 | 4,70E+06 | 4,70E+06 | 2,90E+07 | 6,30E+06 | 1,20E+07 |
| Q7KYK3 | 2,09E+06 | 1,82E+06 | 4,39E+06 | 4,39E+06 | 2,72E+06 | 3,26E+06 | 1,90E+06 | 1,90E+06 | 1,90E+06 | 1,90E+06 | 1,90E+06 | 1,90E+06 |
| Q7Z5A3 | 2,42E+06 | 2,10E+06 | 5,08E+06 | 5,08E+06 | 3,15E+06 | 3,78E+06 | 2,20E+06 | 2,20E+06 | 2,20E+06 | 2,60E+06 | 2,60E+10 | 6,26E+09 |
| Q86SQ4 | 4,20E+07 | 8,40E+07 | 2,00E+07 | 2,00E+07 | 2,00E+07 | 2,00E+07 | 1,50E+07 | 1,50E+07 | 1,50E+07 | 1,50E+07 | 1,50E+07 | 1,50E+07 |
| Q86U12 | 1,98E+07 | 1,72E+07 | 4,16E+07 | 4,16E+07 | 2,57E+07 | 3,09E+07 | 1,80E+07 | 2,40E+04 | 1,90E+06 | 1,80E+07 | 2,40E+04 | 1,90E+06 |
| Q8IU80 | 4,20E+06 | 8,40E+06 | 2,00E+07 | 2,00E+07 | 2,00E+07 | 2,00E+07 | 3,10E+07 | 3,10E+07 | 3,10E+07 | 3,10E+07 | 3,10E+07 | 3,10E+07 |
| Q8IW41 | 4,40E+06 | 4,40E+06 | 1,50E+07 | 4,50E+07 | 3,80E+06 | 1,14E+07 | 1,40E+07 | 1,40E+07 | 1,40E+07 | 1,40E+07 | 1,40E+07 | 1,40E+07 |
| Q8N5Z7 | 9,46E+06 | 8,23E+06 | 1,99E+07 | 1,99E+07 | 1,23E+07 | 1,48E+07 | 8,60E+06 | 8,60E+06 | 8,60E+06 | 8,60E+06 | 8,60E+06 | 8,60E+06 |
| Q8N9T9 | 2,86E+06 | 2,49E+06 | 6,01E+06 | 6,01E+06 | 3,72E+06 | 4,46E+06 | 2,60E+06 | 2,60E+06 | 2,60E+06 | 2,60E+06 | 2,60E+06 | 2,00E+06 |
| Q8NBJ5 | 6,00E+07 | 3,00E+07 | 3,80E+06 | 1,90E+06 | 1,50E+08 | 5,00E+07 | 1,50E+07 | 1,00E+07 | 3,80E+06 | 3,80E+06 | 3,80E+06 | 3,80E+06 |
| Q8NHW5 | 2,09E+06 | 1,82E+06 | 4,39E+06 | 4,39E+06 | 2,72E+06 | 3,26E+06 | 1,90E+06 | 1,46E+06 | 2,92E+06 | 4,09E+06 | 2,63E+06 | 3,42E+06 |
| Q8TBA7 | 1,98E+07 | 1,72E+07 | 4,16E+07 | 4,16E+07 | 2,57E+07 | 3,09E+07 | 1,80E+07 | 1,00E+03 | 1,90E+06 | 1,80E+07 | 1,00E+03 | 1,90E+06 |

| | | | | | | | | | | | | |
|---|---|---|---|---|---|---|---|---|---|---|---|---|
| Q8TBK5 | 9,46E+06 | 8,23E+06 | 1,99E+07 | 1,99E+07 | 1,23E+07 | 1,48E+07 | 8,60E+06 | 8,60E+06 | 8,60E+06 | 8,60E+06 | 8,60E+06 | 8,60E+06 |
| Q8TD27 | 2,09E+06 | 1,82E+06 | 4,39E+06 | 4,39E+06 | 2,72E+06 | 3,26E+06 | 1,90E+06 | 1,46E+06 | 2,92E+06 | 4,09E+06 | 2,63E+06 | 3,42E+06 |
| Q8WVW5 | 2,97E+06 | 2,58E+06 | 6,24E+06 | 6,24E+06 | 3,86E+06 | 4,63E+06 | 2,70E+06 | 2,70E+06 | 2,70E+06 | 4,90E+07 | 4,90E+07 | 4,90E+07 |
| Q8WYN9 | 7,59E+07 | 6,60E+07 | 1,59E+08 | 1,59E+08 | 9,87E+07 | 1,18E+08 | 6,90E+07 | 6,90E+07 | 6,90E+07 | 6,90E+07 | 6,90E+07 | 6,90E+07 |
| Q96AM7 | 2,64E+04 | 2,30E+04 | 5,54E+04 | 5,54E+04 | 3,43E+04 | 4,12E+04 | 2,40E+04 | 2,40E+04 | 2,40E+04 | 2,40E+04 | 1,50E+06 | 2,40E+04 |
| Q96C32 | 1,36E+09 | 1,19E+09 | 2,86E+09 | 2,86E+09 | 1,77E+09 | 2,13E+09 | 1,24E+09 | 1,24E+09 | 1,24E+09 | 1,24E+09 | 1,24E+09 | 1,24E+09 |
| Q96C96 | 1,65E+06 | 1,43E+06 | 3,47E+06 | 3,47E+06 | 2,15E+06 | 2,57E+06 | 1,50E+06 | 1,50E+06 | 1,50E+06 | 1,50E+06 | 1,50E+06 | 1,50E+06 |
| Q96DG6 | 1,65E+06 | 1,43E+06 | 3,47E+06 | 3,47E+06 | 2,15E+06 | 2,57E+06 | 1,50E+06 | 1,50E+06 | 1,50E+06 | 1,50E+06 | 1,50E+06 | 1,50E+06 |
| Q96H31 | 1,36E+09 | 1,19E+09 | 2,86E+09 | 2,86E+09 | 1,77E+09 | 2,13E+09 | 1,24E+09 | 1,24E+09 | 1,24E+09 | 1,24E+09 | 1,24E+09 | 1,24E+09 |
| Q96HX7 | 1,98E+07 | 1,72E+07 | 4,16E+07 | 4,16E+07 | 2,57E+07 | 3,09E+07 | 1,80E+07 | 1,00E+03 | 1,90E+06 | 1,80E+07 | 1,00E+03 | 1,90E+06 |
| Q96MH4 | 2,09E+07 | 1,82E+07 | 4,39E+07 | 4,39E+07 | 2,72E+07 | 3,26E+07 | 1,90E+07 | 1,90E+07 | 1,90E+07 | 1,90E+07 | 1,90E+07 | 1,90E+07 |
| Q96QB7 | 2,20E+07 | 1,91E+07 | 4,62E+07 | 4,62E+07 | 2,86E+07 | 3,43E+07 | 2,00E+07 | 2,00E+07 | 2,00E+07 | 2,00E+07 | 2,00E+07 | 2,00E+07 |
| Q96RE1 | 1,39E+10 | 1,21E+10 | 2,91E+10 | 2,91E+10 | 1,80E+10 | 2,16E+10 | 1,26E+10 | 1,26E+10 | 1,26E+10 | 1,26E+10 | 1,26E+10 | 1,26E+10 |
| Q96RS2 | 1,39E+10 | 1,21E+10 | 2,91E+10 | 2,91E+10 | 1,80E+10 | 2,16E+10 | 1,26E+10 | 9,69E+09 | 1,94E+10 | 2,71E+10 | 1,74E+10 | 2,27E+10 |
| Q99623 | 6,86E+07 | 5,97E+07 | 1,44E+08 | 1,44E+08 | 8,92E+07 | 1,07E+08 | 6,24E+07 | 6,24E+07 | 6,24E+07 | 6,24E+07 | 6,24E+07 | 6,24E+07 |
| Q9BQ02 | 1,06E+07 | 9,18E+06 | 2,22E+07 | 2,22E+07 | 1,37E+07 | 1,65E+07 | 9,60E+06 | 9,60E+06 | 9,60E+06 | 9,60E+06 | 9,60E+06 | 9,60E+06 |
| Q9H2I7 | 2,86E+06 | 2,49E+06 | 6,01E+06 | 6,01E+06 | 3,72E+06 | 4,46E+06 | 2,60E+06 | 2,60E+06 | 2,60E+06 | 6,80E+06 | 2,60E+06 | 2,60E+06 |
| Q9H8T0 | 4,40E+06 | 4,40E+06 | 1,50E+07 | 4,50E+07 | 3,80E+06 | 1,14E+07 | 1,00E+07 | 1,00E+07 | 1,00E+07 | 1,00E+07 | 1,00E+07 | 1,00E+07 |
| Q9HBB3 | 9,46E+06 | 8,23E+06 | 1,99E+07 | 1,99E+07 | 1,23E+07 | 1,48E+07 | 8,60E+06 | 8,60E+06 | 8,60E+06 | 8,60E+06 | 8,60E+06 | 8,60E+06 |
| Q9NUV1 | 2,86E+06 | 2,49E+06 | 6,01E+06 | 6,01E+06 | 3,72E+06 | 4,46E+06 | 2,60E+06 | 2,60E+06 | 2,60E+06 | 2,60E+06 | 2,60E+06 | 2,60E+06 |
| Q9NW02 | 2,86E+06 | 2,49E+06 | 6,01E+06 | 6,01E+06 | 3,72E+06 | 4,46E+06 | 2,60E+06 | 2,60E+06 | 2,60E+06 | 2,60E+06 | 2,60E+06 | 2,60E+06 |
| Q9NZE6 | 7,92E+05 | 6,89E+05 | 1,66E+06 | 1,66E+06 | 1,03E+06 | 1,24E+06 | 7,20E+05 | 7,20E+05 | 7,20E+05 | 5,30E+06 | 5,30E+06 | 5,30E+06 |
| Q9NZS6 | 2,86E+06 | 2,49E+06 | 6,01E+06 | 6,01E+06 | 3,72E+06 | 4,46E+06 | 2,60E+06 | 2,60E+06 | 2,60E+06 | 1,40E+07 | 2,60E+06 | 2,60E+06 |
| Q9UBK2 | 6,00E+07 | 6,00E+07 | 1,20E+07 | 3,60E+07 | 3,80E+06 | 1,14E+07 | 1,00E+07 | 1,00E+07 | 1,00E+07 | 1,00E+07 | 1,00E+07 | 1,00E+07 |
| Q9UFQ0 | 1,36E+09 | 1,19E+09 | 2,86E+09 | 2,86E+09 | 1,77E+09 | 2,13E+09 | 1,24E+09 | 1,24E+09 | 1,24E+09 | 1,24E+09 | 1,24E+09 | 1,24E+09 |
| Q9UG59 | 2,64E+04 | 2,30E+04 | 5,54E+04 | 5,54E+04 | 3,43E+04 | 4,12E+04 | 2,40E+04 | 2,40E+04 | 2,40E+04 | 2,40E+04 | 1,50E+06 | 2,40E+04 |
| Q9UKK9 | 2,86E+06 | 2,49E+06 | 6,01E+06 | 6,01E+06 | 3,72E+06 | 4,46E+06 | 2,60E+06 | 2,60E+06 | 2,60E+06 | 3,70E+06 | 2,60E+06 | 2,60E+06 |
| Q9UL44 | 2,00E+07 | 2,00E+07 | 8,30E+06 | 2,49E+07 | 6,00E+07 | 1,80E+08 | 3,10E+07 | 3,10E+07 | 3,10E+07 | 3,10E+07 | 3,10E+07 | 3,10E+07 |
| Q9Y6D3 | 2,00E+07 | 2,00E+07 | 7,20E+06 | 2,16E+07 | 6,10E+07 | 1,83E+08 | 3,10E+07 | 3,10E+07 | 3,10E+07 | 3,10E+07 | 3,10E+07 | 3,10E+07 |

| | | | | | | | | | | | | |
|---|---|---|---|---|---|---|---|---|---|---|---|---|
| R4GN08 | 1,39E+10 | 1,21E+10 | 2,91E+10 | 2,91E+10 | 1,80E+10 | 2,16E+10 | 1,26E+10 | 9,69E+09 | 1,94E+10 | 2,71E+10 | 1,74E+10 | 2,27E+10 |
| S4R3Z2 | 3,63E+06 | 3,16E+06 | 7,62E+06 | 7,62E+06 | 4,72E+06 | 5,66E+06 | 3,30E+06 | 2,54E+06 | 5,08E+06 | 7,11E+06 | 4,57E+06 | 5,94E+06 |
| U3KPZ0 | 1,32E+07 | 1,15E+07 | 2,77E+07 | 2,77E+07 | 1,72E+07 | 2,06E+07 | 1,20E+07 | 1,20E+07 | 1,20E+07 | 1,20E+07 | 1,20E+07 | 1,20E+07 |
| U3KQF3 | 1,32E+07 | 1,15E+07 | 2,77E+07 | 2,77E+07 | 1,72E+07 | 2,06E+07 | 1,20E+07 | 1,20E+07 | 1,20E+07 | 1,20E+07 | 1,20E+07 | 1,20E+07 |
| U3PXP0 | 1,39E+10 | 1,21E+10 | 2,91E+10 | 2,91E+10 | 1,80E+10 | 2,16E+10 | 1,26E+10 | 9,69E+09 | 1,94E+10 | 2,71E+10 | 1,74E+10 | 2,27E+10 |
| V9GYG0 | 8,47E+06 | 7,37E+06 | 1,78E+07 | 1,78E+07 | 1,10E+07 | 1,32E+07 | 7,70E+06 | 7,70E+06 | 7,70E+06 | 7,70E+06 | 7,70E+06 | 7,70E+06 |
| V9HVX6 | 2,09E+06 | 1,82E+06 | 4,39E+06 | 4,39E+06 | 2,72E+06 | 3,26E+06 | 1,90E+06 | 1,46E+06 | 2,92E+06 | 4,09E+06 | 2,63E+06 | 3,42E+06 |
| V9HW26 | 4,40E+06 | 3,83E+06 | 9,24E+06 | 9,24E+06 | 5,72E+06 | 6,86E+06 | 4,00E+06 | 4,00E+06 | 4,00E+06 | 4,00E+06 | 4,00E+06 | 4,00E+06 |
| V9HW31 | 1,98E+07 | 1,72E+07 | 4,16E+07 | 4,16E+07 | 2,57E+07 | 3,09E+07 | 1,80E+07 | 1,80E+07 | 1,80E+07 | 1,80E+07 | 1,80E+07 | 1,80E+07 |
| V9HW88 | 3,85E+06 | 3,35E+06 | 8,09E+06 | 8,09E+06 | 5,01E+06 | 6,01E+06 | 3,50E+06 | 3,50E+06 | 3,50E+06 | 3,50E+06 | 3,50E+06 | 3,50E+06 |
| V9HWB4 | 1,36E+08 | 1,19E+08 | 2,86E+08 | 2,86E+08 | 1,77E+08 | 2,13E+08 | 1,24E+08 | 1,24E+08 | 1,24E+08 | 1,24E+08 | 1,24E+08 | 1,24E+08 |
| V9HWE0 | 4,95E+06 | 4,30E+06 | 1,04E+07 | 1,04E+07 | 6,44E+06 | 7,72E+06 | 4,50E+06 | 4,50E+06 | 4,50E+06 | 2,60E+07 | 1,60E+06 | 3,40E+06 |
| V9HWE1 | 1,65E+06 | 1,43E+06 | 3,47E+06 | 3,47E+06 | 2,15E+06 | 2,57E+06 | 1,50E+06 | 1,50E+06 | 1,50E+06 | 1,50E+06 | 1,50E+06 | 1,50E+06 |
| V9HWE9 | 6,86E+07 | 5,97E+07 | 1,44E+08 | 1,44E+08 | 8,92E+07 | 1,07E+08 | 6,24E+07 | 6,24E+07 | 6,24E+07 | 6,24E+07 | 6,24E+07 | 6,24E+07 |
| V9HWG9 | 2,42E+05 | 2,10E+05 | 5,08E+05 | 5,08E+05 | 3,15E+05 | 3,78E+05 | 2,20E+05 | 2,20E+05 | 2,20E+05 | 1,20E+07 | 1,80E+06 | 2,40E+04 |
| V9HWH9 | 1,98E+06 | 1,72E+06 | 4,16E+06 | 4,16E+06 | 2,57E+06 | 3,09E+06 | 1,80E+06 | 1,80E+06 | 1,80E+06 | 1,80E+06 | 1,80E+06 | 1,80E+06 |
| V9HWI3 | 1,54E+07 | 1,34E+07 | 3,23E+07 | 3,23E+07 | 2,00E+07 | 2,40E+07 | 1,40E+07 | 1,40E+07 | 1,40E+07 | 1,40E+07 | 1,40E+07 | 1,40E+07 |
| V9HWJ1 | 2,00E+07 | 2,00E+07 | 7,20E+06 | 2,16E+07 | 3,10E+07 | 9,30E+07 | 1,40E+07 | 1,40E+07 | 1,40E+07 | 1,40E+07 | 1,40E+07 | 1,40E+07 |
| W8QEH3 | 1,43E+07 | 1,24E+07 | 3,00E+07 | 3,00E+07 | 1,86E+07 | 2,23E+07 | 1,30E+07 | 1,30E+07 | 1,30E+07 | 1,30E+07 | 1,30E+07 | 1,30E+07 |
